# Novel [(*N*-alkyl-3-indolylmethylene)hydrazono]oxindoles arrest cell cycle and induce cell apoptosis by inhibiting CDK2 and Bcl-2: synthesis, biological evaluation and *in silico* studies

**DOI:** 10.1080/14756366.2020.1773814

**Published:** 2020-06-11

**Authors:** Tarfah Al-Warhi, Mahmoud F. Abo-Ashour, Hadia Almahli, Ohoud J. Alotaibi, Mohammad M. Al-Sanea, Ghada H. Al-Ansary, Hanaa Y. Ahmed, Mahmoud M. Elaasser, Wagdy M. Eldehna, Hatem A. Abdel-Aziz

**Affiliations:** a Department of Chemistry, College of Science, Princess Nourah bint Abdulrahman University, Riyadh, Saudi Arabia; b Department of Pharmaceutical Chemistry, Faculty of Pharmacy, Egyptian Russian University, Badr City, Cairo, Egypt; c Department of Chemistry, Chemistry Research Laboratory, University of Oxford, Oxford, UK; d Department of Pharmaceutical Chemistry, College of Pharmacy, Jouf University, Sakaka, Aljouf, Saudi Arabia; e Department of Pharmaceutical Chemistry, Pharmacy Program, Batterejee Medical College, Jeddah, Saudi Arabia; f Department of Pharmaceutical Chemistry, Faculty of Pharmacy, Ain Shams University, Cairo, Egypt; g The Regional Center for Mycology and Biotechnology, Al-Azhar University, Cairo, Egypt; h Department of Pharmaceutical Chemistry, Faculty of Pharmacy, Kafrelsheikh University, Kafrelsheikh, Egypt; i Department of Applied Organic Chemistry, National Research Center, Dokki, Giza, Egypt

**Keywords:** Isatin, N-alkylindole, hybridisation, anticancer, Bcl-2 inhibitor, CDK2 inhibitor

## Abstract

As a continuation for our previous work, a novel set of *N*-alkylindole-isatin conjugates (**7**, **8a–c**, **9** and **10a–e**) is here designed and synthesised with the prime aim to develop more efficient isatin-based antitumor candidates. Utilising the SAR outputs from the previous study, our design here is based on appending four alkyl groups with different length (ethyl and n-propyl), bulkiness (iso-propyl) and unsaturation (allyl) on *N*-1 of indole motif, with subsequent conjugation with different *N*-unsubstituted isatin moieties to furnish the target conjugates. As planned, the adopted strategy achieved a substantial improvement in the growth inhibitory profile for the target conjugates in comparison to the reported lead **VI**. The best results were obtained with *N*-propylindole –5-methylisatin hybrid **8a** which displayed broad spectrum anti-proliferative action with efficient sub-panel GI_50_ (MG-MID) range from 1.33 to 4.23 µM, and promising full-panel GI_50_ (MG-MID) equals 3.10 µM, at the NCI five-dose assay. Also, hybrid **8a** was able to provoke cell cycle disturbance and apoptosis in breast T-47D cells as evidenced by the DNA flow cytometry and Annexin V-FITC/PI assays. Furthermore, hybrid **8a** exhibited good inhibitory action against cell cycle regulator CDK2 protein kinase and the anti-apoptotic Bcl-2 protein (IC_50_= 0.85 ± 0.03 and 0.46 ± 0.02 µM, respectively). Interestingly, molecular docking for hybrid **8a** in CDK2 and Bcl-2 active sites unveiled that *N*-propyl group is involved in significant hydrophobic interactions. Taken together, the results suggested conjugate **8a** as a promising lead for further development and optimisation as an efficient antitumor drug.

## 1. Introduction

In the current medical era, cancer is considered as a major public health problem worldwide and one of the most leading causes of death throughout the world. So, development of more effective new drugs for management of different human malignancies is a major requirement. The expanded knowledge for the functional relationship between the different molecules which constitute the cell cycle and checkpoint pathways furnished novel promising strategies for the management of tumours.

Cyclin-dependent kinases (CDKs) are considered as crucial factors that affect diverse key transitions in cell cycle for the cancer cell, in addition to regulation of apoptosis, transcription and exocytosis, therefore therapeutic strategies based on inhibition of CDKs stand out as a promising opportunity for anticancer drug discovery and an efficient approach for management of different human malignancies. On the other hand, apoptosis, an automatic cancer cell death, was found as an important consequence of CDKs inhibition and can be assessed by cell cycle arrest at low concentration or even mitochondrial damage at high concentration^1^. This fact was discovered from previous studies on CDKs inhibitors as Roscovitine and Purvalanol and their effect on three different prostate cancer cell lines^2^. In addition, the link between CDKs and apoptosis was revealed in the investigational research of the effect of Ibulocydine, a prodrug CDK inhibitor, on hepatocellular carcinoma^3^.

**Figure 1.**
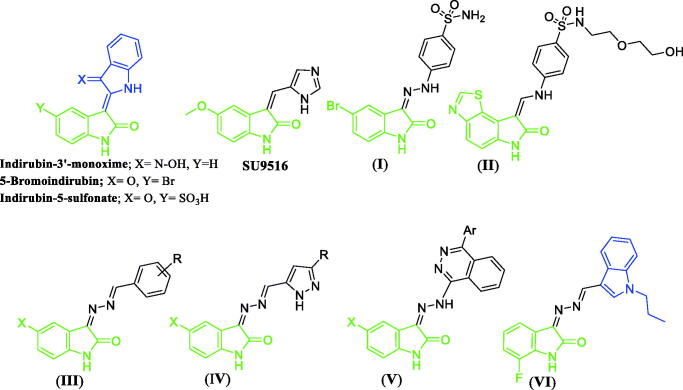
Chemical structures for certain reported isatin-based anticancer agents (**I–V**), and lead compound **VI**.

Isatin moiety served as a contributor in various CDKs inhibitors having an apoptotic effect. For example, isatin dimers, such as Indirubin-3′-oxime (Figure 1), revealed a potent inhibition against CDK1, CDK2, and CDK5 with IC_50_s = 180 nM, 500 nM and 250 nM, respectively^4^. In addition to its effect against CDKs, it possessed an apoptotic effect by disruption of cell cycle phases through arresting G2/M phase^5^. Indirubin-3′-oxime is regarded as a lead compound. It is especially known in Chinese traditional medicine, and its other derivatives as 5-Bromoindirun and Indirubin-5-sulfonate proved to be potent CKD inhibitors against CDK2 with IC_50_s = 1 and 0.5 µM, respectively. Moreover, they were co-crystallized with CDK2 (2BHE and 1E9H, respectively) (Figure 1)^6^^,^^7^. This dimeric scaffold is initiative for the design of other derivatives with a significant activity against CDKs as reported^8^^,^^9^. Other isatin derivatives decorated with 3-substitution expressed potent inhibition for CDK2 as shown in SU9516 which inhibits CDK2 with IC_50_ = 25 nM (Figure 1)^10^. Moreover, compound (**I**) is another isatin derivative with hydrazino linker at position 3 (CDK2; IC_50_ = 60 nM) that was co-crystallized with its target and submitted to protein data bank (1VFT). Several modifications on compound (**I**) were performed to get compound (**II**) which inhibits CDK2 in sub-nanomolar range (IC_50_ = 0.54 nM) (Figure 1)^11^.

In last few years, our research team has developed diverse small molecules based on the isatin scaffold as efficient anticancer agents (structures **III**–**V**^12–14^, Figure 1) with diverse cellular and enzymatic targets; for example triggering of apoptosis in several tumour cell lines^15^^,^^16^, inhibition of tumour-linked human carbonic anhydrase isoform IX^17^^,^^18^, and inhibition of VEGFR-2 kinase^19^. In 2018, we have reported the conjugation between the isatin and indole moieties *via* methylenehydrazono spacer (HC=N–N=) to develop new three different series of [(3-indolylmethylene)hydrazono]indolinone derivatives as anticancer agents with promising pro-apoptotic activity^20^. As concluded from SAR analysis for this study^20^, hybridisation of *N*-propyl indole moiety with *N*-unsubstituted isatin moiety (compound **VI**, Figure 1) achieved the most effective anticancer activity.

Inspired by these findings and in connection with our previous research work, it is thought advantageous to broaden our investigations to probe new *N*-alkylindole-isatin conjugates **7**, **8a–c**, **9** and **10a–e** exerting promising anticancer and pro-apoptotic actions. Utilising the SAR outputs from the previous study^20^, our design here is based on appending four alkyl groups with different length (ethyl **7**, n-propyl **8a–c**), bulkiness (isopropyl **9**) and unsaturation (allyl **10a–e**) on *N*-1 of indole motif, with subsequent conjugation with different *N*-unsubstituted isatin moieties to furnish the target conjugates.

All the synthesised conjugates were examined for their cytotoxicity towards a list of three different human cancer cell lines HCT-116 (Colon), A-549 (NSCLC), and MDA-MB-231 (Breast) utilising SRB assay. Furthermore, seven hybrids (**8a–c**, **9**, **10a**, **10c** and **10d**) were screened for their possible *in vitro* anticancer action in accordance with US-NCI protocol, then, **8a** was furthermore chosen for testing at the five-dose assay. Subsequently, we examined the growth inhibition mechanism of hybrid **8a** in relation to cell cycle regulation as well as apoptosis induction in breast T-47D cancer cells through the DNA flow cytometry and Annexin V-FITC/PI assays. Moreover, inhibitory actions of hybrid **8a** against the cell cycle regulator protein CDK2 kinase and the anti-apoptotic protein Bcl-2 were explored. Finally, docking simulations were conducted in order to explore the behaviour of **8a** within the active site of both CDK2 and Bcl-2, and to justify its activity.

## 2. Results and discussion

### 2.1. Chemistry

**Scheme 1.**
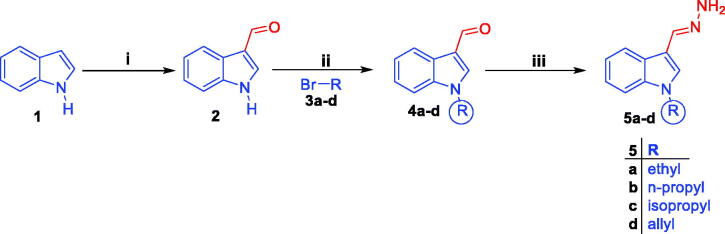
Preparation of the key intermediates **5a–d**; (**i**) DMF, POCl_3_, reflux 8 h.; (**ii**) DMF, NaH, stirring at R.T for 24 h.; (**iii**) Ethyl alcohol, NH_2_NH_2_·H_2_O, reflux 2 h.

**Scheme 2.**
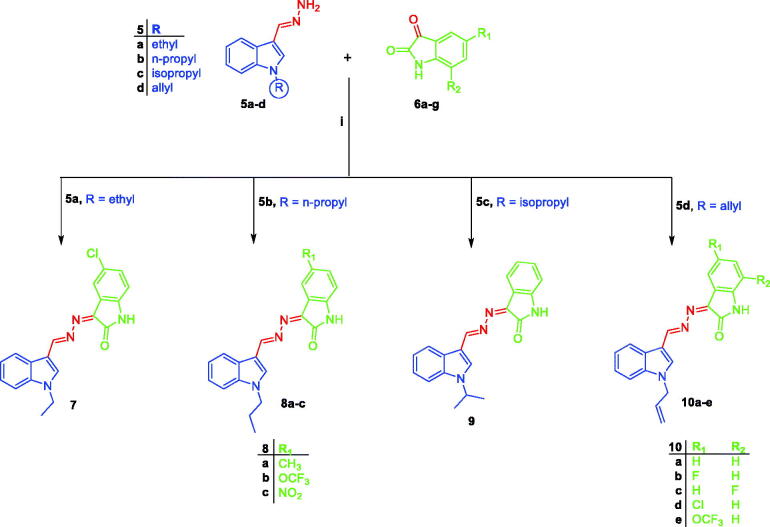
Synthesis of target conjugates **7, 8a–c, 9** and **10a-e**; (**i**) Ethyl alcohol, acetic acid, reflux 3 h.

Synthetic routes herein proposed in order to get the targeted conjugates (**7**, **8a–c**, **9** and **10a–e**) have been illustrated in Schemes 1 and 2. With regard to Scheme 1, preparation of 1*H*-indole-3-carbaldehyde **2** was achieved through Vilsmeier formylation of indole **1** by the use of *N*, *N*-dimethylformamide (DMF) and phosphorus oxychloride (POCl_3_), then aldehyde **2** was undergone to *N*-alkylation using different alkyl halides **3a–d** in DMF with the aid of sodium hydride base to get *N*-substituted indole-3-carbaldehyde intermediates **4a–d**. The different *N*-substituted indole-3-carbaldehyde derivatives **4a–d** were condensed with hydrazine hydrate *via* heating under reflux temperature in ethyl alcohol in order to produce the key intermediates *N*-substituted-3-(hydrazonomethyl)-1*H*-indoles **5a–d**. In Scheme 2, the key intermediates **5a–d** were reacted with different isatin derivatives **6a–g** in absolute ethyl alcohol with the aid of catalytic amount of glacial acetic acid to furnish targeted [(3-indolylmethylene)hydrazono]indolinones **7**, **8a–c**, **9** and **10a–e**.

Postulated structures for the newly prepared key intermediates and target compounds **7**, **8a–c**, **9** and **10a–e** have been in full consistent with the data of both spectral and elemental analyses.

### 2.2. Biological evaluation

#### 2.2.1. Antiproliferative activities towards A-549, MDA-MB-231 and HCT-116 cancer cell lines

All the newly prepared target conjugates (**7**, **8a–c**, **9** and **10a–e**) were investigated for the prospective growth inhibitory actions against three different cancer cell lines viz.; A-549, MDA-MB-231, and HCT-116, using the sulforhodamine B colorimetric (SRB) assay^21^. Staurosporine, a clinically used antitumor drug, was co-assayed as a reference agent for the experiment. The results have been conveyed as IC_50_ values, and listed in Table 1.

**Table 1.** Anti-proliferative activity of compounds **7, 8a–c, 9 and 10a–e** against human cancer A-549, MDA-MB-231 and HCT-116 cell lines. 
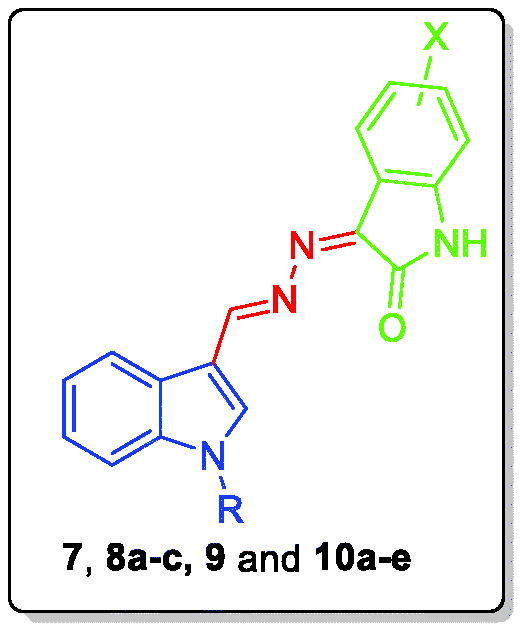

Comp.	R	X	IC_50_ (µM)^a^		
			A-549	MDA-MB-231	HCT-116
**7**	CH_2_CH_3_	5-Cl	50.0 ± 3.37	27.6 ± 1.76	19.7 ± 1.28
**8a**	CH_2_CH_2_CH_3_	5-CH_3_	7.3 ± 0.42	4.7 ± 0.28	2.6 ± 0.17
**8b**	CH_2_CH_2_CH_3_	5-OCF_3_	9.2 ± 0.78	8.9 ± 0.61	19.1 ± 1.34
**8c**	CH_2_CH_2_CH_3_	5-NO_2_	12.3 ± 1.05	15.6 ± 0.98	6.4 ± 0.50
**9**	CH(CH_3_)_2_	H	31.7 ± 2.27	10.4 ± 0.80	28.2 ± 1.73
**10a**	CH_2_CH=CH_2_	H	17.3 ± 1.19	11.7 ± 1.04	7.7 ± 0.68
**10b**	CH_2_CH=CH_2_	5-F	59.9 ± 3.58	54.0 ± 4.17	36.3 ± 2.35
**10c**	CH_2_CH=CH_2_	7-F	43.8 ± 2.96	35.4 ± 1.92	7.3 ± 0.47
**10d**	CH_2_CH=CH_2_	5-Cl	53.1 ± 4.13	42.9 ± 2.51	28.5 ± 1.59
**10e**	CH_2_CH=CH_2_	5-OCF_3_	40.2 ± 1.88	51.8 ± 3.77	37.4 ± 2.06
**Dox.**	**-**	**-**	2.3 ± 0.17	4.5 ± 0.29	3.7 ± 0.24

**^a^**IC_50_ values are the mean ± SE of 3 discrete experiments.

Investigation of the obtained IC_50_ values (Table 1) hinted out that the tested *N*-alkylindole-isatin derivatives (**7**, **8a–c**, **9** and **10a–e**) were more effective against colon HCT-116 cells than NSCLC A-549 and TNBC MDA-MB-231 cancer cells, except compound **8b** that displayed enhanced anti-proliferative potency against A-549 and MDA-MB-231 cell lines (IC_50_ = 9.2 ± 0.78 and 8.9 ± 0.61 µM, correspondingly) than HCT-116 cells (IC_50_ = 19.1 ± 1.34 µM), and compound **9** that exhibited slightly enhanced antiproliferative action against breast MDA-MB-231 cells (IC_50_ = 10.4 ± 0.80 µM) than colon HCT-116 cells (IC_50_ = 28.2 ± 1.73 µM).

With respect to the cytotoxic activity against colon HCT-116 cell line, hybrid **8a** stood out as the most potent analogue herein reported with IC_50_ = 2.6 ± 0.17 µM, which is more effective than reference drug doxorubicin (IC_50_ = 3.7 ± 0.24 µM). Also, conjugates **8c**, **10a** and **10c** exhibited potent anti-proliferative activity (IC_50_ = 6.4 ± 0.50, 7.7 ± 0.68 and 7.3 ± 0.47 µM, respectively) with about 2-fold decreased efficiency than doxorubicin. Moreover, compounds **7** and **8b** possessed moderate potency with IC_50_ values equal 19.7 ± 1.28 and 19.1 ± 1.34 µM, respectively, whereas compounds **9**, **10b**, **10d** and **10e** elicited weak growth inhibitory activity towards HCT-116 cells (IC_50_ = 28.2 ± 1.73, 36.3 ± 2.35, 28.5 ± 1.59 and 37.4 ± 2.06 µM, respectively).

Exploring anti-proliferative activities towards NSCLC A-549 cells revealed that hybrid **8a** was the most efficient counterpart (IC_50_ = 7.3 ± 0.42 µM), with about 3-fold dwindled efficiency compared to the reference compound doxorubicin (IC_50_ = 2.3 ± 0.17 µM) against A-549 cells. Moreover, compounds **8b**, **8c** and **10a** exhibited efficient anti-proliferative action towards A-549 cells with IC_50_ values equal 9.2 ± 0.78, 12.3 ± 1.05 and 17.3 ± 1.19 µM, respectively. With regards to the cytotoxicity against the breast MDA-MB-231 cell line, the data shown in Table 1 ascribed to the examined hybrids excellent to weak efficacy in inhibiting the proliferation of MDA-MB-231 cells with IC_50_ values ranging between 4.7 ± 0.28 and 42.9 ± 2.51 µM, except compounds **10b** and **10e** that failed to inhibit the growth of breast MDA-MB-231 cells up to 50 µM (IC_50_ = 54.0 ± 4.17, and 51.8 ± 3.77 µM, respectively). Superiorly, compound **8a** emerged as the most effective derivative in the current study towards MDA-MB-231 cells with IC_50_ value equals 4.7 ± 0.28 µM. Besides, compounds **8b**, **8c**, **9** and **10a** displayed good anti-proliferative activity against MDA-MB-231 cells (IC_50_ = 8.9 ± 0.61, 15.6 ± 0.98, 10.4 ± 0.80 and 11.7 ± 1.04 µM, respectively).

#### 2.2.2. Nci-USA cancer cell lines screening

Seven hybrids (**8a–c**, **9**, **10a**, **10c** and **10d**) have been selected and screened by USA-National Cancer Institute (NCI-DTP; www.dtp.nci.nih.gov) for their possible *in vitro* anti-proliferative actions towards a panel of 59 human cancer cell lines representing breast, ovarian, CNS, colon, NSCLC, leukaemia, melanoma, renal and prostate cancers, according to the NCI, Bethesda, Drug Evaluation Branch protocol^22–24^.

##### 2.2.2.1. Preliminary single (10 µM) dose screening

Antitumor activities of here reported conjugates **8a–c**, **9**, **10a**, **10c** and **10d** were first screened in an initial single dose (10 µM) screening, utilising the SRB assay to estimate cells viability and growth^21^. The obtained results were presented as percent growth inhibition (GI %) for the examined conjugates towards the different treated tumour cell lines, as presented in Table 2.

**Table 2.** *In vitro* GI % for compounds (**8a–c, 9** and **10a, c, d**), at 10 μM concentration, towards the subpanel cancer cell lines

	Compound^a^						
Subpanel / Cell line	8a	8b	8c	9	10a	10c	10d
Leukaemia							
CCRF-CEM	101	52	–	–	–	17	–
HL-60(TB)	123	12	10	18	22	–	–
K-562	83	33	18	–	30	20	–
MOLT-4	110	50	–	13	35	19	14
RPMI-8226	125	30	–	–	13	–	–
SR	89	–	19	11	50	58	33
Non-Small Cell Lung Cancer							
A549/ATCC	36	28	13	–	–	–	–
EKVX	50	40	–	38	–	15	33
HOP-62	25	21	–	–	–	–	–
HOP-92	63	–	11	14	–	–	–
NCI-H226	116	–	–	16	13	56	41
NCI-H23	52	12	–	–	12	–	–
NCI-H322M	53	–	–	–	–	–	–
NCI-H460	68	11	19	–	–	–	–
NCI-H522	86	19	–	10	31	34	17
Colon cancer							
COLO 205	116	–	–	–	30	–	–
HCC-2998	53	–	–	–	–	–	–
HCT-116	60	21	20	–	24	28	–
HCT-15	77	23	–	–	15	22	10
HT29	83	25	–	–	10	–	–
KM12	40	–	11	–	–	–	–
SW-620	64	–	–	–	–	–	–
CNS cancer							
SF-268	54	–	–	–	–	–	–
SF-295	29	–	–	–	–	–	–
SF-539	66	–	10	–	–	–	–
SNB-19	37	–	22	–	–	–	–
SNB-75	15	–	–	33	18	–	–
U251	60	13.32	28	–	10	–	–
Melanoma							
LOX IMVI	69	–	–	–	–	–	–
MALME-3M	49	–	–	–	26	–	–
M14	44	–	–	–	12	–	–
MDA-MB-435	127	–	–	–	–	–	–
SK-MEL-2	65	–	–	–	–	–	–
SK-MEL-28	41	–	–	–	–	–	–
SK-MEL-5	51	–	–	–	–	–	–
UACC-257	41	–	–	–	–	–	–
UACC-62	60	11	–	–	25	15	19
Ovarian cancer							
IGROV1	61	–	–	–	28	31	23
OVCAR-3	98	–	–	–	21	13	–
OVCAR-4	58	–	–	–	27	22	–
OVCAR-5	16	–	–	–	–	–	–
OVCAR-8	53	15	29	–	15	11	–
NCI/ADR-RES	24	15	–	–	–	–	–
SK-OV-3	12	–	–	–	–	–	–
Renal cancer							
786-0	36	–	–	–	–	–	–
A498	43	–	–	–	–	–	–
ACHN	45	15	–	–	–	11	–
RXF 393	89	–	–	–	11	–	–
SN12C	51	–	–	–	–	–	–
TK-10	–	–	–	–	–	–	–
UO-31	40	19	–	14	33	29	23
Prostate							
PC-3	82	32	–	–	22	10	–
DU-145	34	13	–	–	–	–	–
MCF7	91	29	15	10	33	31	18
Breast cancer							
MDA-MB-231	66	–	–	11	–	–	–
HS 578T	26	–	–	–	–	–	–
T-47D	61	35	–	13	56	50	30
MDA-MB-468	75	11	17	–	20	54	36
BT-549	37	–	–	–	–	–	–
Sensitive cell lines no.	58	26	14	12	27	20	12

^a^Only GI % higher than 10% are shown.

Exploring the attained GI% values (Table 2), disclosed that hybrid **8a** stood out as the most efficient anti-proliferative agent in the herein reported NCI screening exhibiting mean GI % equals to 59, with broad-spectrum action towards all human cancer cell lines in all the herein examined cancer subpanels, except towards Renal cancer TK-10 cell line.

**Figure 2.**
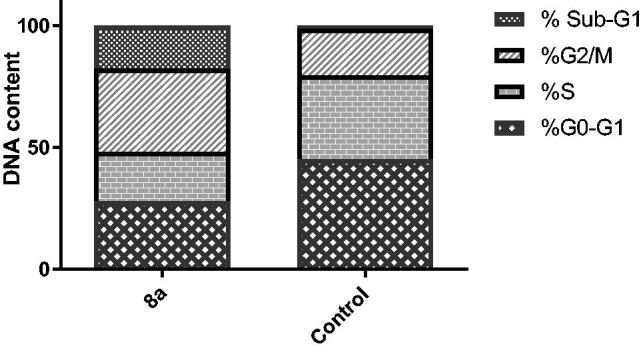
Impact of hybrid **8a** on the cell cycle progression in breast cancer T-47D cells.

Remarkably, conjugate **8a** showed excellent growth inhibition properties against Leukaemia (K-562 and SR), NSCLC (NCI-H522), Colon cancer (HT29), Ovarian cancer (OVCAR-3), Prostate cancer (PC-3), Renal cancer (RXF 393), and Breast cancer (MCF7) cell lines with percentage inhibition of 83, 89, 86, 83, 98, 89, 82 and 91%, respectively (Table 2, Figure 2). Furthermore, compound **8a** exerted good activity with growth inhibition % equal to or greater than 60 towards NSCLC (HOP-92 and NCI-H460), Colon cancer (SW-620, HCT-116 and HCT-15), CNS cancer (SF-539 and U251), Melanoma (LOX IMVI, SK-MEL-2 and UACC-62), Ovarian cancer (IGROV1) and Breast cancer (MDA-MB-231, T-47D and MDA-MB-468) cell lines with inhibition percent of 63, 68, 60, 77, 64, 66, 60, 69, 65, 60, 61, 66, 61 and 75% respectivly (Table 2, Figure 2). On the other hand, the remaining herein examined hybrids **8b**, **8c**, **9**, **10a**, **10c** and **10d** displayed moderate to weak antitumor activities (GI % range: 4–11). It is worth stressing that hybrid **8a** exerted a cytotoxic impact with GI % >100 against some tumour cell lines. Hybrid **8a** was shown to be lethal towards Leukaemia (HL-60(TB), CCRF-CEM, MOLT-4 and RPMI-8226) cells with GI % values equal to 101, 123, 110 and 125, respectively, whereas, **8a** exerted its lethal action towards Colon cancer HCT-116 and Melanoma MDA-MB-435 cells with GI % = 116 and 126, respectively (Table 2).

##### 2.2.2.2. *In vitro* full NCI panel five dose assay

The obtained data from the preliminary single dose (10 µM) screening disclosed that hybrid **8a** (NSC: D-771612/1) is the most efficient anticancer agent here in this study, displaying promising effectiveness against numerous cancer cell lines from different subpanels (Table 2, Figure 2). Subsequently, hybrid **8a** was chosen to carry out further biological screening at a (0.01–100 µM) five-dose assay. Three response parameters (GI_50_, TGI and LC_50_) were evaluated for hybrid **8a** towards each herein examined cancer cell line, and listed in Table 3. GI_50_ values reflect the level for growth inhibitory effect, whereas TGI represents cytostatic impact. In addition, LC_50_ parameter is considered to be the cytotoxicity parameter for the examined hybrid. Moreover, both full panel and subpanel mean graph midpoints (MG-MID), for full panel and individual subpanels cell lines, were calculated on the level of GI_50_ parameter, providing an average potency parameter for the tested hybrid **8a** (Table 4).

**Table 3.** Results of NCI-USA *in vitro* five-dose testing for conjugate **8a** (NSC: D- 795311/1).

Subpanel/tumour cell lines	Compound			
	8a			
	GI_50_ (µM)	TGI (µM)		LC_50_ (µM)
Leukaemia				
CCRF-CEM	1.94		>100	>100
HL60(TB)	1.31		4.80	>100
K-562	2.22		20.5	>100
MOLT-4	0.68		4.54	>100
RPMI-8226	1.33		3.96	>100
SR	0.52		8.72	>100
Non-small cell lung cancer				
A549/ATCC	7.18		39.6	>100
EKVX	2.36		>100	>100
HOP-62	4.17		25.0	>100
HOP-92	1.87		6.57	>100
NCI-H226	2.95		20.6	>100
NCI-H23	3.13		38.2	>100
NCI-H322M	3.42		>100	>100
NCI-H460	2.17		10.6	75.7
NCI-H522	1.81		6.59	>100
Colon cancer				
COLO 205	1.25		3.06	7.46
HCC-2998	2.09		5.14	19.0
HCT-116	1.75		4.17	9.96
HCT-15	2.63		42.2	>100
HT29	3.4		>100	>100
KM12	3.22		19.7	>100
SW-620	2.97		35.3	>100
CNS cancer				
SF-268	3.33		28.0	>100
SF-295	8.41		96.6	>100
SF-539	2.69		17.1	>100
SNB-19	4.24		>100	>100
SNB-75	2.31		46.0	>100
U251	3.51		17.3	>100
Melanoma				
LOX IMVI	1.26		3.06	7.41
MALME-3M	1.88		6.03	100
M14	1.92		4.70	17.2
MDA-MB-435	1.50		2.96	5.85
SK-MEL-2	2.42		8.03	>100
SK-MEL-28	4.70		36.9	>100
SK-MEL-5	2.86		13.50	39.0
UACC-257	12.30		66.60	>100
UACC-62	2.24		12.10	>100
Ovarian cancer				
IGROV1	3.38		63.10	>100
OVCAR-3	1.36		5.11	>100
OVCAR-4	0.93		25.60	>100
OVCAR-5	4.04		25.8	>100
OVCAR-8	5.41		>100	>100
NCI/ADR-RES	6.39		89.90	>100
SK-OV-3	1.17		67.10	>100
Renal cancer				
786-0	5.94		71.20	>100
A498	1.37		7.50	>100
ACHN	3.56		>100	>100
RXF 393	2.17		7.94	>100
SN12C	3.20		75.90	>100
TK-10	11.10		>100	>100
UO-31	2.28		6.18	>100
Prostate cancer				
PC-3	1.83		5.17	47.40
DU-145	3.88		40.60	>100
Breast cancer				
MCF7	1.54		15.50	>100
MDA-MB-231/ATCC	1.81		7.40	>100
HS 578T	11.3		>100	>100
BT-549	1.79		6.33	>100
T-47D	0.41		18.40	>100
MDA-MB-468	1.69		5.78	79.40

NT: not tested.

**Table 4.** Median GI_50_ (µM) values for subpanel cancer cell lines for conjugate **8a**.

Subpanel tumour cell line	8a	
	MG-MID	Selectivity index
Leukaemia	1.33	2.33
NSCL Cancer	3.22	0.96
Colon Cancer	2.47	1.25
CNS Cancer	4.08	0.75
Melanoma	3.45	0.89
Ovarian Cancer	3.24	0.95
Renal Cancer	4.23	0.73
Prostate Cancer	2.85	1.08
Breast Cancer	3.09	1.00
Full panel MG-MID	3.10	

As shown in Table 3, hybrid **8a** displayed potent anti-proliferative action at a single-digit micromolar concentration against all herein examined human cancer cell lines with GI_50_ values range: 1.17–8.41 µM, except for Melanoma (UACC-257), Renal (TK-10) and Breast (HS 578 T) cell lines which possessed GI_50_ values equal 12.30, 11.10 and 11.30 µM, respectively (Table 3). Interestingly, hybrid **8a** showed superior sub-micromolar activity towards leukaemia (MOLT-4 and SR), Ovarian cancer (OVCAR-4) and Breast cancer (T-47D) cells (GI_50_ = 0.68, 0.52, 0.93 and 0.41 µM, respectively).

In addition, hybrid **8a** showed potent cytostatic activity at single-digit micromolar concentration (TGI range: 2.96–8.03 µM) against 22 cancer cell lines belonging to all herein examined cancer subpanels, except CNS cancer subpanel (Table 3). While hybrid **8a** had no cytostatic impact (TGI > 100 µM) against leukaemia (CCRF-CEM), NSCLC (EKVX and NCI-H322M), colon cancer (HT29), CNS cancer (SNB-19), renal cancer (ACHN and TK-10), ovarian cancer (OVCAR-8), and breast cancer (HS 578 T) cells, it exhibited good to weak cytostatic activity towards the remaining cancer cell lines with TGI spanning in the interval: 10.6–96.6 µM. On the other hand, compound **8a** emerged as a non-lethal agent that possesses LC_50_ values more than 100 µM for the most of cancer cell lines herein examined, except for Colon cancer (COLO 205, HCC-2998 and HCT-116), NSCLC (NCI-H460), Prostate cancer (PC-3), Melanoma (LOX IMVI, M14, MDA-MB-435 and SK-MEL-5), and Breast cancer (MDA-MB-468) cell lines (LC_50_ = 75.7, 7.46, 19.0, 9.96, 7.41, 17.2, 5.85, 39.0, 47.40 and 79.40 µM, respectively (Table 3).

With regards to the sensitivity for diverse tumour cell lines, hybrid **8a** possessed relatively homologous growth inhibitory action for the whole of NCI panel, with efficient sub-panel GI_50_ (MG-MID) range of 1.33–4.23 µM, and promising full-panel GI_50_ (MG-MID) = 3.10 µM. leukaemia, prostate cancer, colon cancer, and breast cancer subpanels were the most vulnerable herein examined cancer subpanels to the impact of hybrid **8a** [GI_50_ (MG-MID) = 1.33, 2.47, 2.85 and 3.09 µM, respectively] (Table 4).

Furthermore, dividing the full-panel MG-MID (µM) for the examined derivative by its individual subpanel MG-MID (µM) provides an index which is regarded as a measure for its selectivity. A value between three and six refers to moderate selectivity against the corresponding cancer cell line, ratio more than six indicates high selectivity, whereas if the compound does not meet any of such criteria is considered as non-selective^25^. In this regards, hybrid **8a** was found to be a non-selective anticancer agent that exhibits broad-spectrum potency towards all cancer subpanels herein examined at the GI_50_ level, with selectivity ratios spanning in the range from 0.73 to 2.33 (Table 4).

#### 2.2.3. Cell cycle analysis

The superior sub-micromolar anti-proliferative activity of conjugate **8a** on breast cancer T-47D cells (GI_50_ = 0.41 µM, Table 3) prompted us to further investigate about the growth inhibitory mechanism of the target conjugates. Both regulation of cell cycle progression and apoptosis induction have been considered as significant strategies to control the proliferation of different cancer cells, accordingly, we primarily examined the growth inhibition mechanism of hybrid **8** in relation to cell cycle progression and regulation in human breast T-47D cancer cells.

**Figure 3.**
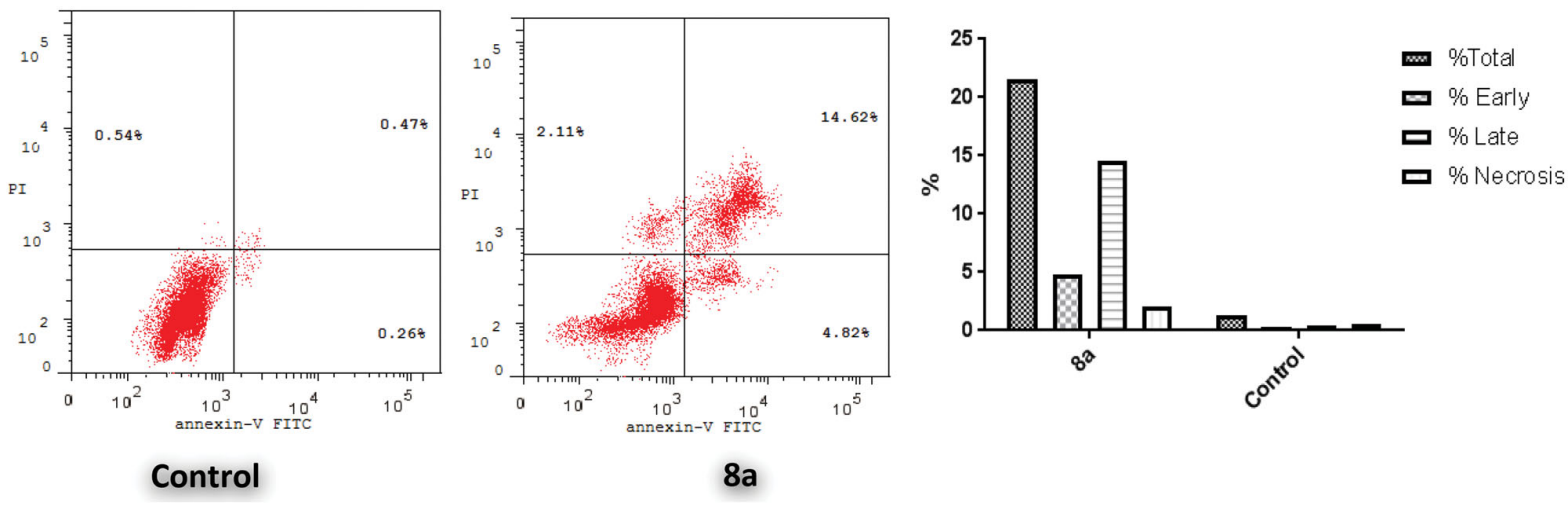
Effect of conjugate **8a** over the AV-FITC-positive staining percentages in breast T-47D cells.

The impact on cell cycle distribution was assessed by a DNA flow cytometry analysis, upon incubation of T-47D cells with conjugate **8a** at its GI_50_ concentration (0.41 µM) for 24 h (Figure 3). From the obtained results it was found that T-47D cells exposed to hybrid **8** significantly arrested at the G2/M phase of the cell cycle with an escalation in G2/M phase fraction from 19.19% (in control cells) to 34.05% (in **8a**-treated T-47D cells). Furthermore, the cell population in sub-G1 phase was drastically augmented from 1.25% (in control cells) to 17.73% (in **8a**-treated T-47D cells).

Generally the upsurge of populations in the sub-G1 phase indicates the induction of apoptotic cell death. So, a subsequent study will be conducted to reveal whether the G2/M phase cell cycle arrest afforded by conjugate **8a** was accompanied by apoptosis.

#### 2.2.4. Apoptosis assay

**Figure 4.**
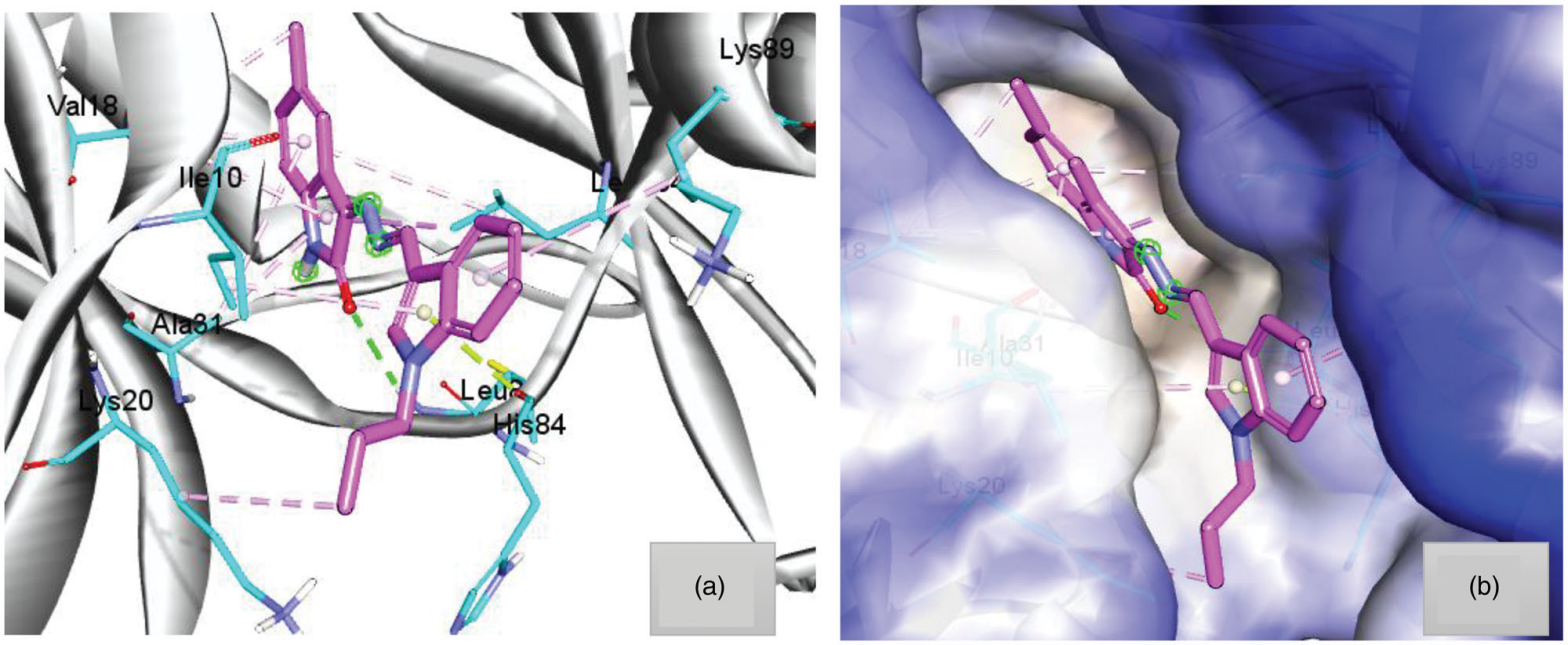
(a) Docking pose of hybrid **8a** within the active site of CDK2 (pdb code 2BHE) showing hydrogen bond with Leu83 (green dots) and hydrophobic interaction (light purple dots). (b) Hydrophobic surface of active site of CDK2 surround compound **8a**.

In an attempt to further investigate whether the antiproliferative activity for conjugate **8a** is harmonious with the apoptosis induction within T-47D cells pointed out by the increased cell population in sub-G1 phase in **8a**-treated T-47D cells (Figure 3), Annexin V-FITC/PI dual staining analysis was used for the apoptosis assay (Figure 4).

The outcomes of the Annexin V-FITC/PI assay suggested that treatment of T-47D cells with conjugate **8a** led to an early and late cellular apoptosis, which proved through the significant increase for the apoptotic cells percentage in both the early apoptotic phase (from 0.26% to 4.82%) and the late apoptotic phase (from 0.47% to 14.62%) that signifies about 26-fold increase in total apoptosis, when compared to the untreated control (Figure 4).

#### 2.2.5. Cdk2 and Bcl-2 inhibitory activity

The promising anti-proliferative impact of conjugate **8a**, in addition to its cell cycle disruption and pro-apoptotic effects, provoked a further exploration for their possible inhibitory activities against the cell cycle regulator CDK2 protein kinase and the anti-apoptotic Bcl-2 protein. The results expressed as IC_50_ values are presented in Table 5.

**Table 5.** Inhibitory activities of compound **8a** against CDK2 and Bcl-2.

Compound	IC_50_ (μM)^a^	
	CDK2	Bcl-2
**8a**	0.85 ± 0.03	0.46 ± 0.02
Roscovitine	0.1 ± 0.01	–
Venetoclax	–	0.09 ± 0.01

**^a^**IC_50_ values are the mean ± SE of three separate experiments.

Results in Table 5 showed that the tested hybrid **8a** exhibited good inhibitory action against CDK2 and Bcl-2 with IC_50_ values equal 0.85 ± 0.03 and 0.46 ± 0.02 µM, respectively.

## 3. Molecular docking studies

The docking simulation studies were conducted to investigate the behaviour of compound **8a** in the active site of both CDK2 and Bcl-2 (pdb code 2BHE^7^ and 4^26^). From several X-ray structures for CDK2 enzyme, as mentioned before in the introduction, it was observed that the active site should be filled by planar molecules^8–11^. In the docking simulations **8a** has taken the planar orientation, although it has a certain flexibility, with some little deviation at the indole moiety to fill the part of the corresponding hydrophobic pocket by hydrophobic contact with Lys89 as shown in (Figure 4(b)), whereas the *N*-propyl group interacted with the part of the terminal hydrophobic pocket by alkyl hydrophobic contact with Lys20. Moreover, the Molecular docking simulations showed an important hydrogen bond interaction with Leu83, *via* C=O of isatin, which is a key interaction with distance 2.5 Å. Also the docking poses of hybrid **8a** showed that 5-methyl group, decorated on the benzenoid part of the isatin moiety, made an anchoring hydrophobic mixed with π interactions with Val18 and Leu134 from both sides as shown in Figure 4(a).

On the other hand, the molecular docking studies explored the binding pattern of hybrid **8a** within the active site of Bcl-2 to justify its apoptotic effect. As reported, the pro-apoptotic effect of the small molecules could be done at the molecular level by mimicking the BH3 α-helix and then binding to the hydrophobic groove of anti-apoptotic Bcl-2 proteins preventing their heterodimerization, which eventually leads to apoptosis^27^. The attitude of α-helical BH3 domains complexed with Bcl-2 family besides the docking poses of other small molecules as Bcl-2 inhibitors were discussed as a guide for our docking study^28–31^.

**Figure 5.**
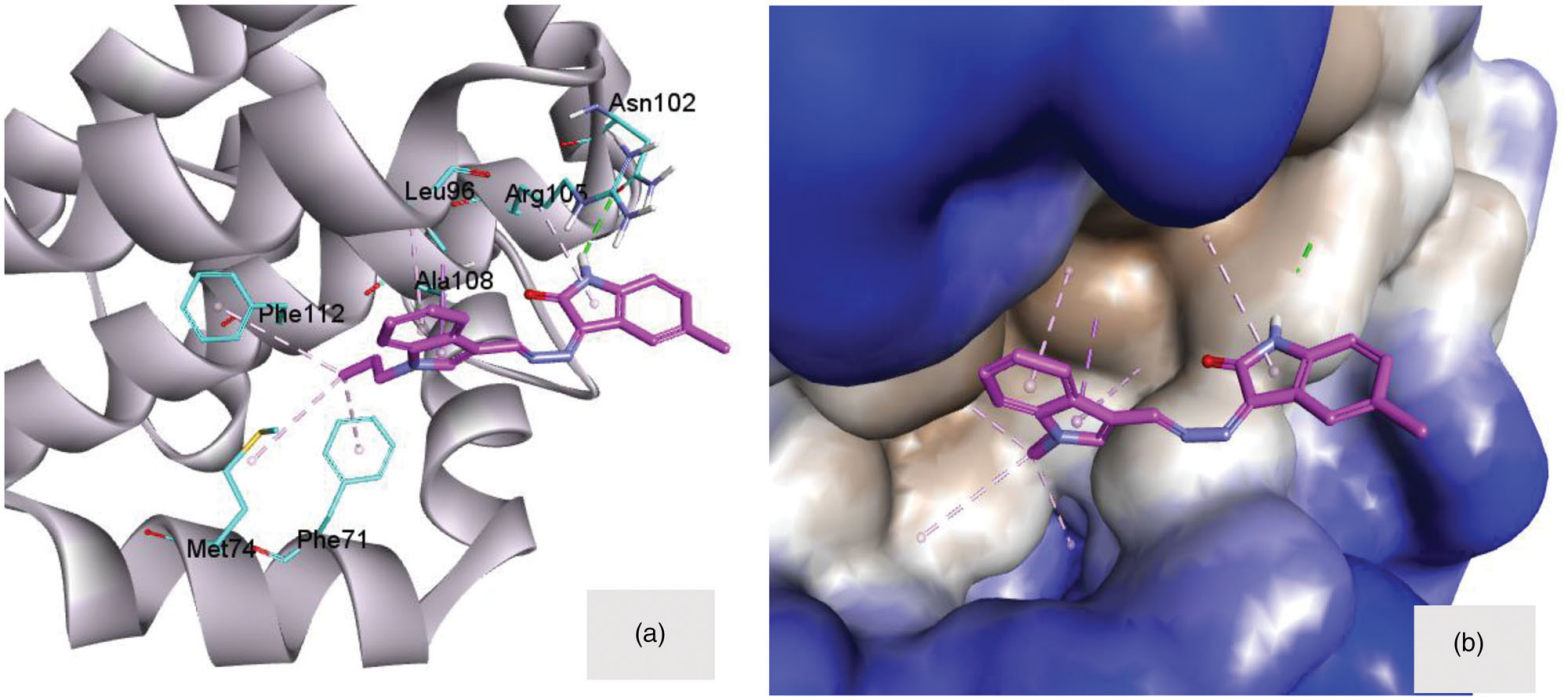
**(a)** Compound **8a** docked into the groove of Bcl-2 (4AQ3) showing the different interaction from both sides to fill this groove. **(b)** Compound **8a** embedded inside the active site of Bcl-2. The macromolecule was surrounded by hydrophobic surface to focus on the hydrophobic contacts.

Molecular docking of compound **8a**, as a small molecule inhibitor, inside the hydrophobic groove of Bcl-2 showed significant interactions at the front of this groove (Figure 5(a)). The (NH) functionality of isatin moiety can donate a hydrogen bond (2.00 Å) to the key amino acid Asn102 at the margin of the hydrophobic groove. This anchoring hydrogen bond was supported by the π-stack of isatin ring with Arg105 (Figure 5(a)). On the other side of the groove, *N*-propyl group attached to indole ring was embedded in the hydrophobic pocket formed of three amino acids; Phe112, Phe71 and Met74. The π-stack interaction of indole ring with Leu96 and Ala108 would act as a middle support for the whole compound inside the opening of the hydrophobic groove (Figure 5(b)).

## 4. Experimental

### 4.1. Chemistry

#### 4.1.1. General

Melting points were measured with a Stuart melting point apparatus, and are uncorrected. Nuclear magnetic resonance; ^1^H NMR and ^13^C NMR spectra were recorded using a Bruker NMR spectrometer (400/100 MHz), in deuterated dimethylsulphoxide (DMSO-d_6_). Chemical shifts (*δ*_H_) were reported relative to TMS as an internal standard. The coupling constant values (*J*) were given in hertz. Chemical shifts (*δ*_C_) were reported as follows: s, singlet; d, doublet; m, multiplet. HRMS spectra were performed on a Bruker MicroTOF spectrometer. Infra-red (IR) Spectra are obtained by the use of Schimadzu FT-IR 8400S spectrophotometer as KBr discs and expressed in wave number (cm^−1^). Compounds **2** and **4a–d** were prepared as reported previously^32^.

#### 4.1.2. *Synthesis of N-substituted-3-(hydrazonomethyl)-1H-indole derivatives* 5a–d

To a solution of *N*-substituted-1*H*-indole-3-carbaldehyde derivatives **4a–d** (4 mmol) in ethyl alcohol (25 ml), an excess of 99% hydrazine hydrate (1.25 ml, 25 mmol) was added. The reaction mixture was stirred at reflux temperature for 2 h, and then filtrated upon cooling. The obtained precipitate was washed with water several times, dried and recrystallized from isopropyl alcohol to furnish *N*-substituted-3-(hydrazonomethyl)-1*H*-indole derivatives **5a–d**, respectively.

#### 4.1.3. *General procedures for preparation of target compounds* 7, 8a–c, 9 *and* 10a–e

To a hot stirred solution of isatin derivatives **6a–g** (1 mmol) in absolute ethyl alcohol (5 ml) and catalytic drops of acetic acid, the appropriate *N*-substituted-3-(hydrazonomethyl)-1*H*-indole derivative **5a–d** (1 mmol) was added. The resulting mixture was stirred at reflux for 3 h, and then filtrated. The collected precipitate was dried and then recrystallized from isopropyl alcohol to afford target hybrids **7**, **8a–c**, **9** and **10a–e**, respectively.

Full characterisation for the key intermediates (**5a**, **5c** and **5d**) as well as the target conjugates (**7**, **8a–c**, **9** and **10a–e**) have been presented in the Supporting Information.

### 4.2. Biological evaluation

Experimental procedures for biological evaluations for the herein reported conjugates (**7**, **8a-c**, **9** and **10a-e**) were provided in the Supplementary material (Supplementary materials can be found at www.mdpi.com/xxx/s1).

#### 4.2.1. Antiproliferative action against A-549, MDAMB-231 and HCT-116 cancer cell lines

The three examined cancer cell lines (non-small cell lung A-549, Breast MDA-MB-231 and colon HCT-116 cells) were obtained from American Type Culture Collection (ATCC). Examination of the cytotoxic activity for the target conjugates was carried out in accordance with the SRB colorimetric assay protocol^21^, as described previously^33^.

#### 4.2.2. Nci-60 cancer cell lines screening

The NCI-USA anticancer screening was carried out following the NCI, Bethesda, Drug Evaluation Branch protocol^22–24^, utilising the SRB protein assay^21^, as reported earlier^34^^,^^35^.

#### 4.2.3. Cell cycle analysis

Flow cytometric analysis (FACS) was carried out to examine the cell cycle distributions in breast T-47D cancer cells upon treatment with conjugate **8a**, by BD FACS Calibur flow cytometer, as described earlier^36^.

#### 4.2.4. Apoptosis assay

Phosphatidylserine externalisation effect of conjugate **8a** over breast T-47D cancer cell line was investigated using Annexin-V-FITC Apoptosis Detection Kit by flow cytometry, as described earlier^37^.

#### 4.2.5. Cdk2 and bcl-2 inhibitory activity

These assays were carried out as reported earlier^38^^,^^39^.

### 4.3. Molecular docking studies

The target proteins, CDK2 (2BHE) and BCl-2 (4AQ3), were downloaded from protein databank and prepared by the default protocol in Discovery Studio 4. All other steps including preparation of small molecules, determination of the active site and docking protocol were followed as reported^40^.

## 5. Conclusions

To conclude, a novel series of *N*-alkylindole-isatin conjugates (**7**, **8a–c**, **9** and **10a-e**) was designed and synthesised, utilising the SAR outputs from the previous study. The design is based on appending four alkyl groups with different length (ethyl, n-propyl), bulkiness (isopropyl) and unsaturation (allyl) on *N*-1 of indole motif, with subsequent conjugation with different *N*-unsubstituted isatin moieties to furnish the target conjugates. As planned, the adopted strategy achieved a substantial improvement in the growth inhibitory profile for the target conjugates in comparison to the reported lead **VI**. All conjugates were screened for their potential cytotoxicity towards a panel of three different human cancer cell lines HCT-116 (Colon), A-549 (NSCLC), and MDA-MB-231 (Breast) utilising SRB assay. Conjugate **8a**, superiorly, displayed the best anti-proliferative activity against the three examined cell lines (IC_50_ = 7.3 ± 0.42, 4.7 ± 0.28 and 2.6 ± 0.17 µM, respectively). Furthermore, **8a** displayed broad spectrum anti-proliferative action with efficient subpanel GI_50_ (MG-MID) range of 1.33–4.23 µM, and promising full panel GI_50_ (MG-MID) = 3.10 µM, at the NCI five-dose assay. In particular, hybrid **8a** showed superior sub-micromolar activity towards leukaemia (MOLT-4 and SR), ovarian cancer (OVCAR-4) and breast cancer (T-47D) cell lines (GI_50_ = 0.68, 0.52, 0.93 and 0.41 µM, respectively). Conjugate **8a** was able to provoke cell cycle disturbance and apoptosis in breast T-47D cells as evidenced by the DNA flow cytometry and Annexin V-FITC/PI assays. Moreover, hybrid **8a** exhibited good inhibitory action against cell cycle regulator CDK2 protein kinase and the anti-apoptotic Bcl-2 protein (IC_50_ = 0.85 ± 0.03 and 0.46 ± 0.02 µM, respectively). The molecular docking for hybrid **8a** in CDK2 and Bcl-2 active sites unveiled that *N*-propyl group is involved in significant hydrophobic interactions. Taken together, the results suggested conjugate **8a** as a promising lead for further development and optimisation as an efficient antitumor drug.

## Supplementary Material

Supplemental Material
