# Supplementary material for: Novel [(N-alkyl-3-indolylmethylene)hydrazono]oxindoles arrest cell cycle and induce cell apoptosis by inhibiting CDK2 and Bcl-2: synthesis, biological evaluation and in silico studies
Source: J Enzyme Inhib Med Chem. 2020 Jun 11;35(1):1300–9. doi: 10.1080/14756366.2020.1773814 (PMC7717600; doi:10.1080/14756366.2020.1773814)
Supplement: Supplemental Material [file IENZ_A_1773814_SM4762.pdf]

Supporting Information

Novel [(*N*-alkyl-3-indolylmethylene)hydrazono]oxindoles arrest cell cycle and induce cell apoptosis by inhibiting CDK2 and Bcl-2: Synthesis, biological evaluation and *in silico* studies

Tarfah Al-Warhi, Mahmoud F. Abo-Ashour, Hadia Almahli, Ohoud J. Alotaibi, Mohammad M. Al-Sanea, Ghada H. Al-Ansary, Hanaa Y. Ahmed, Mahmoud M. Elaasser, Wagdy M. Eldehna*, Hatem A. Abdel-Aziz

1. Representative NMR and HRMS spectra. 2-29

2. Growth percent graph for compound **8a** in the single-dose NCI assay. 30

3. Five-dose NCI assay results for compound **8a**. 31-34

4. Experimental procedures for the biological Evaluation. 35-37

4. Characterisation of intermediates (**5a,c,d**) and the target conjugates (**7**, **8a-c**, **9** and **10a-e**). 35-37

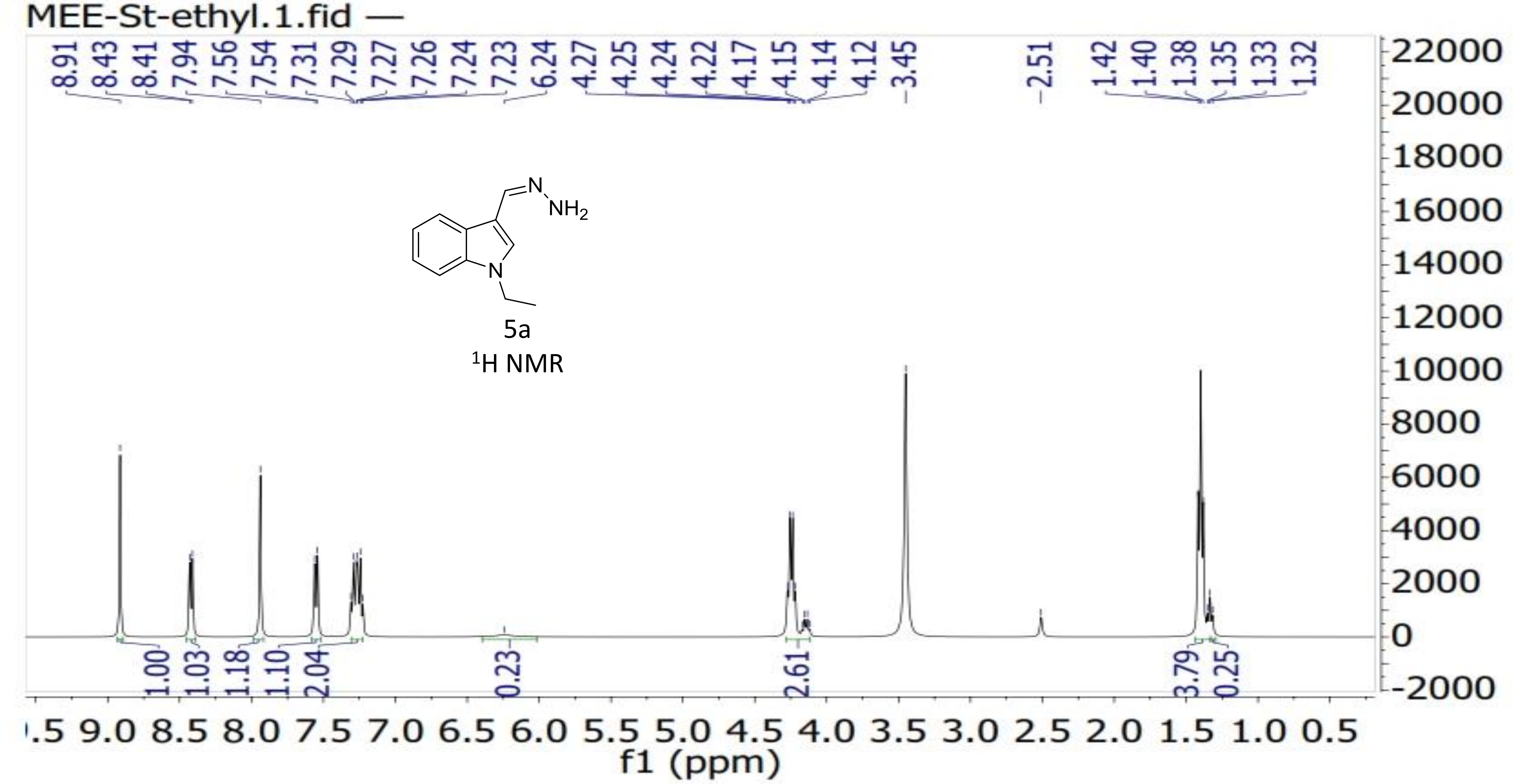
MEE-St-ethyl.1.fid —
8.91 8.43 8.41 7.94 7.56 7.54 7.31 7.29 7.27 7.26 7.24 7.23 6.24 4.27 4.25 4.24 4.22 4.17 4.15 4.14 4.12 3.45 2.51 1.42 1.40 1.38 1.35 1.33 1.32
N
NH2
N
5a
1H NMR
1.00 1.03 1.18 1.10 2.04 0.23 2.61 3.79 0.25
22000 20000 18000 16000 14000 12000 10000 8000 6000 4000 2000 0 -2000
.5 9.0 8.5 8.0 7.5 7.0 6.5 6.0 5.5 5.0 4.5 4.0 3.5 3.0 2.5 2.0 1.5 1.0 0.5
f1 (ppm)

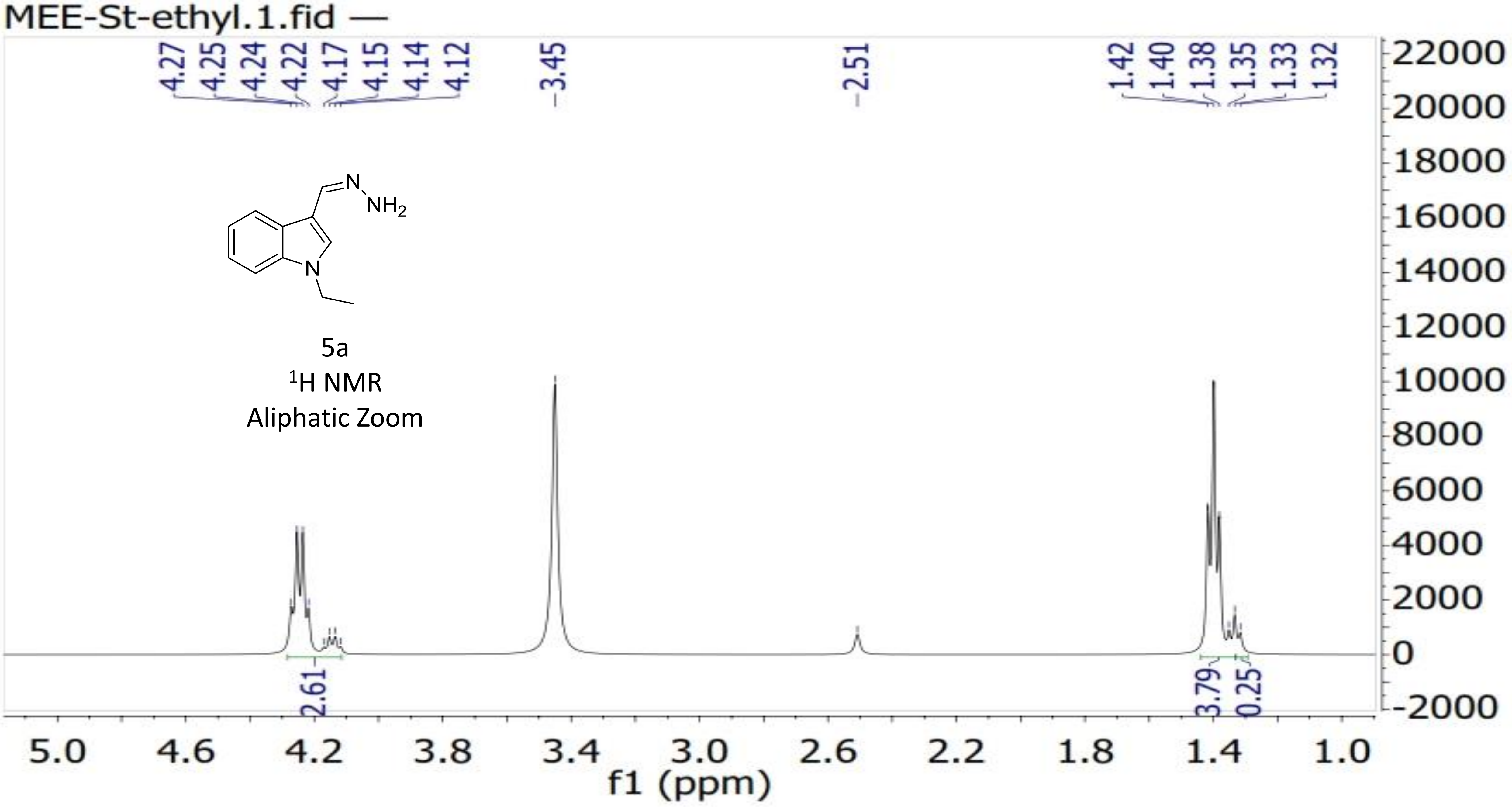
MEE-St-ethyl.1.fid —
4.27 4.25 4.24 4.22 4.17 4.15 4.14 4.12
3.45
2.51
1.42 1.40 1.38 1.35 1.33 1.32
5a
¹H NMR
Aliphatic Zoom
2.61
3.79
0.25
22000
20000
18000
16000
14000
12000
10000
8000
6000
4000
2000
0
-2000
5.0 4.6 4.2 3.8 3.4 3.0 2.6 2.2 1.8 1.4 1.0
f1 (ppm)

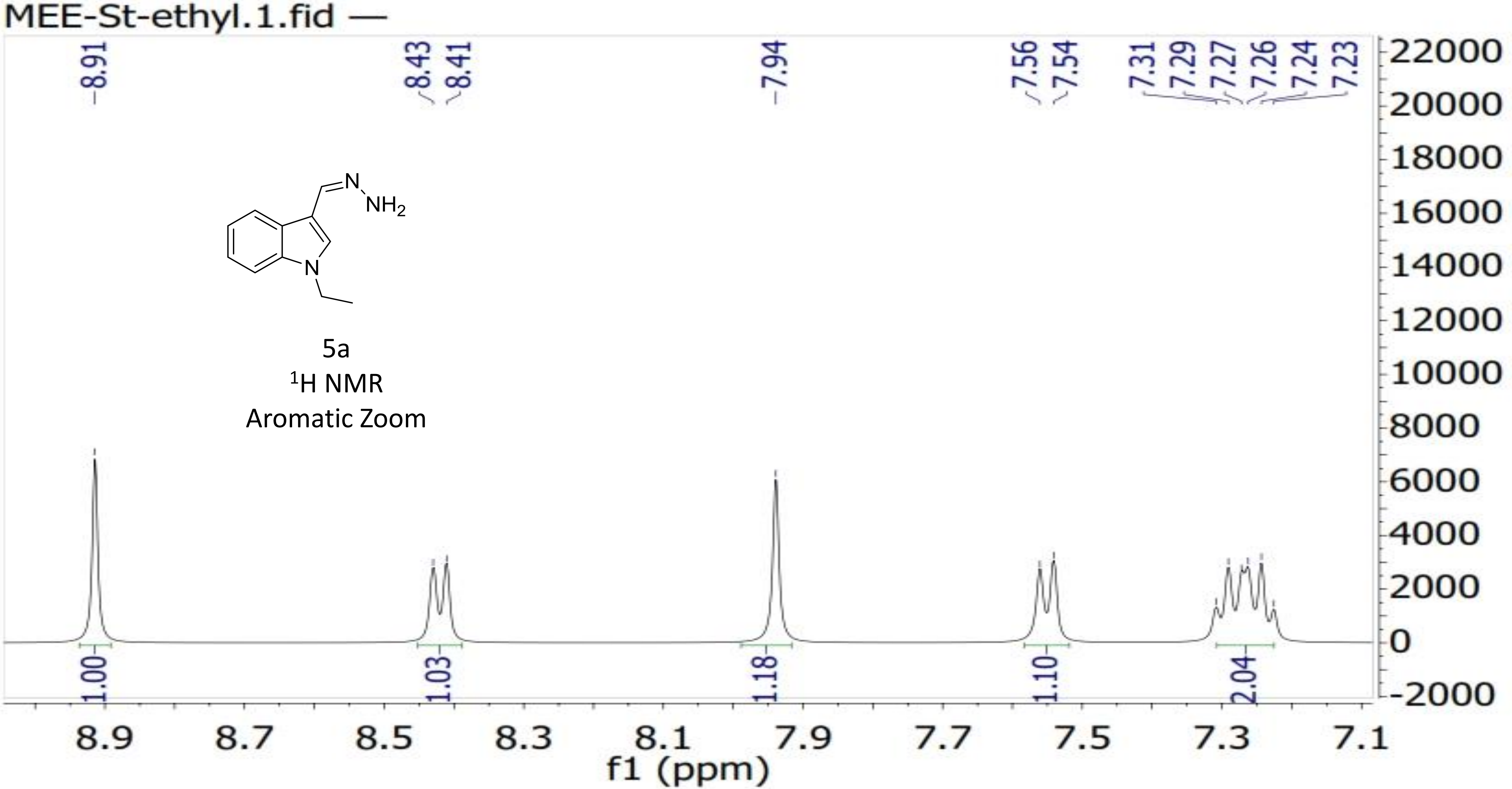
MEE-St-ethyl.1.fid —
8.91
8.43
8.41
7.94
7.56
7.54
7.31
7.29
7.27
7.26
7.24
7.23
N
NH2
N
5a
1H NMR
Aromatic Zoom
1.00
1.03
1.18
1.10
2.04
22000
20000
18000
16000
14000
12000
10000
8000
6000
4000
2000
0
-2000
8.9
8.7
8.5
8.3
8.1
7.9
7.7
7.5
7.3
7.1
f1 (ppm)

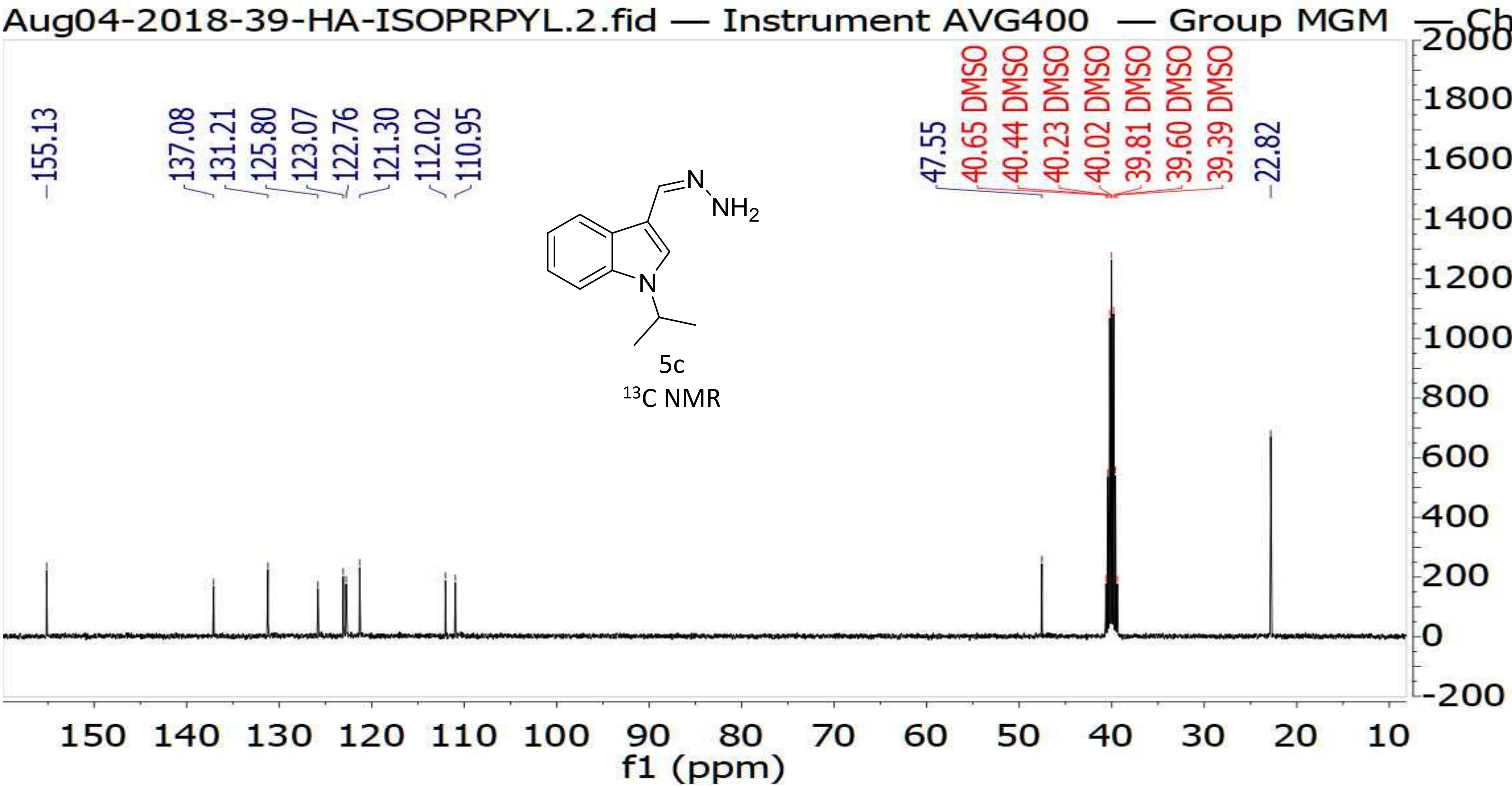

Aug04-2018-39-HA-ISOPRPYL.2.fid — Instrument AVG400 — Group MGM — Ch
155.13
137.08
131.21
125.80
123.07
122.76
121.30
112.02
110.95
47.55
40.65 DMSO
40.44 DMSO
40.23 DMSO
40.02 DMSO
39.81 DMSO
39.60 DMSO
39.39 DMSO
22.82
N
NH2
N
5c
13C NMR
f1 (ppm)

Aug04-2018-54-HA-ALLYL.2.fid — Instrument AVG400 — Group MGM — Chemis

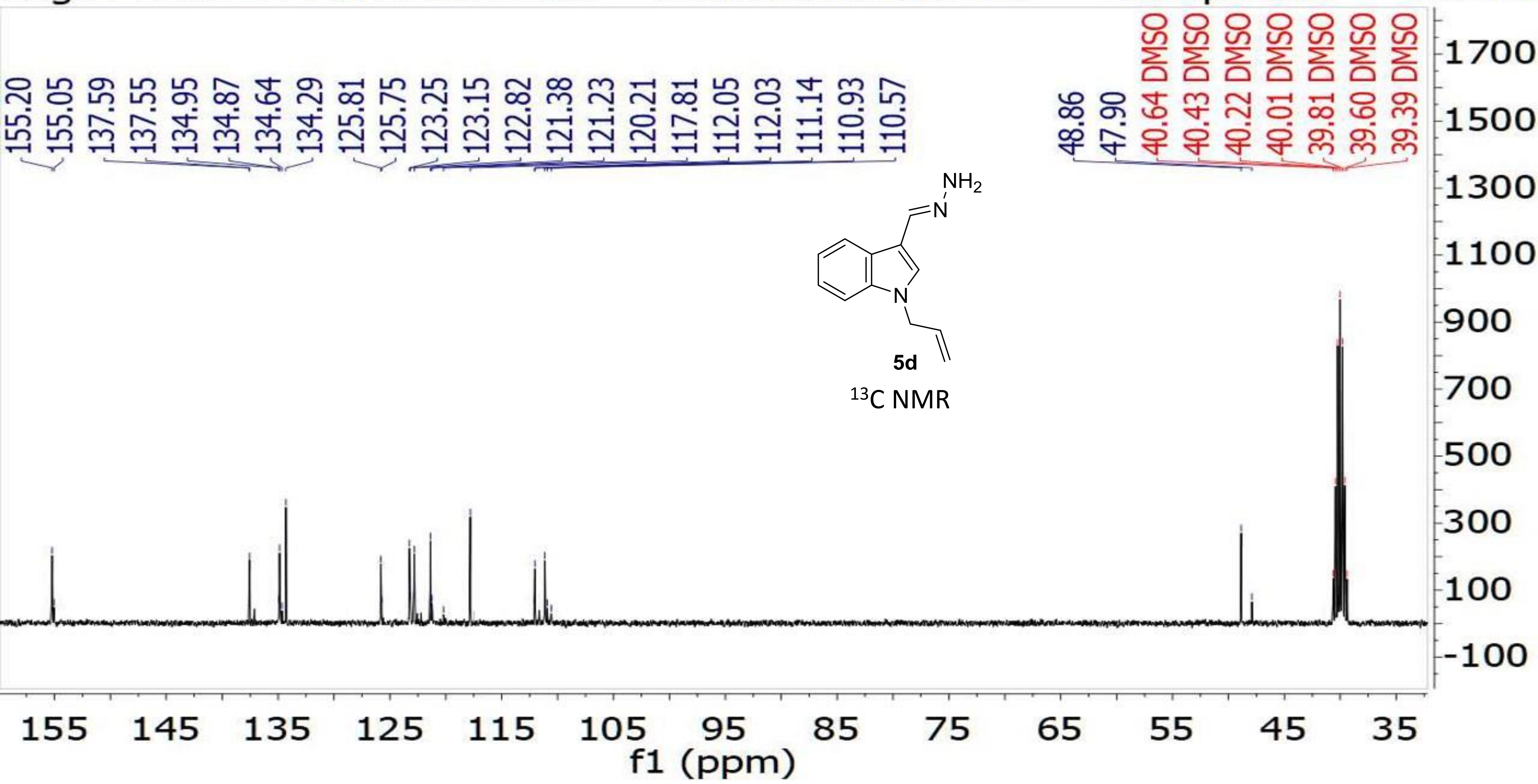

NL: 1.12E6
ESI71805 #14-27 RT: 0.17-0.31 AV: 7 NL: 2.66E+007
T: FTMS {1,1} + p ESI Full ms [80.00-1600.00]

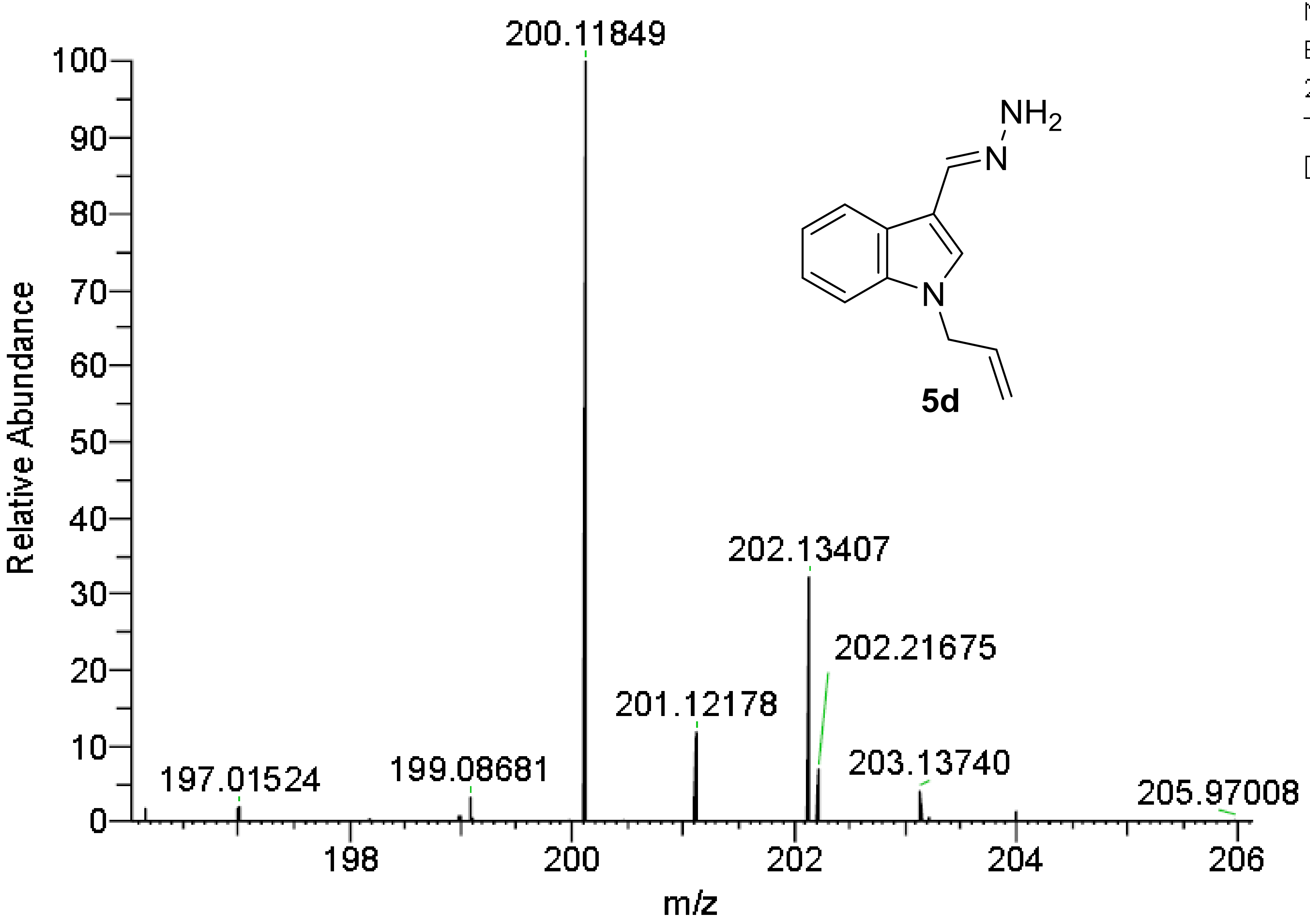

Measured Spectrum

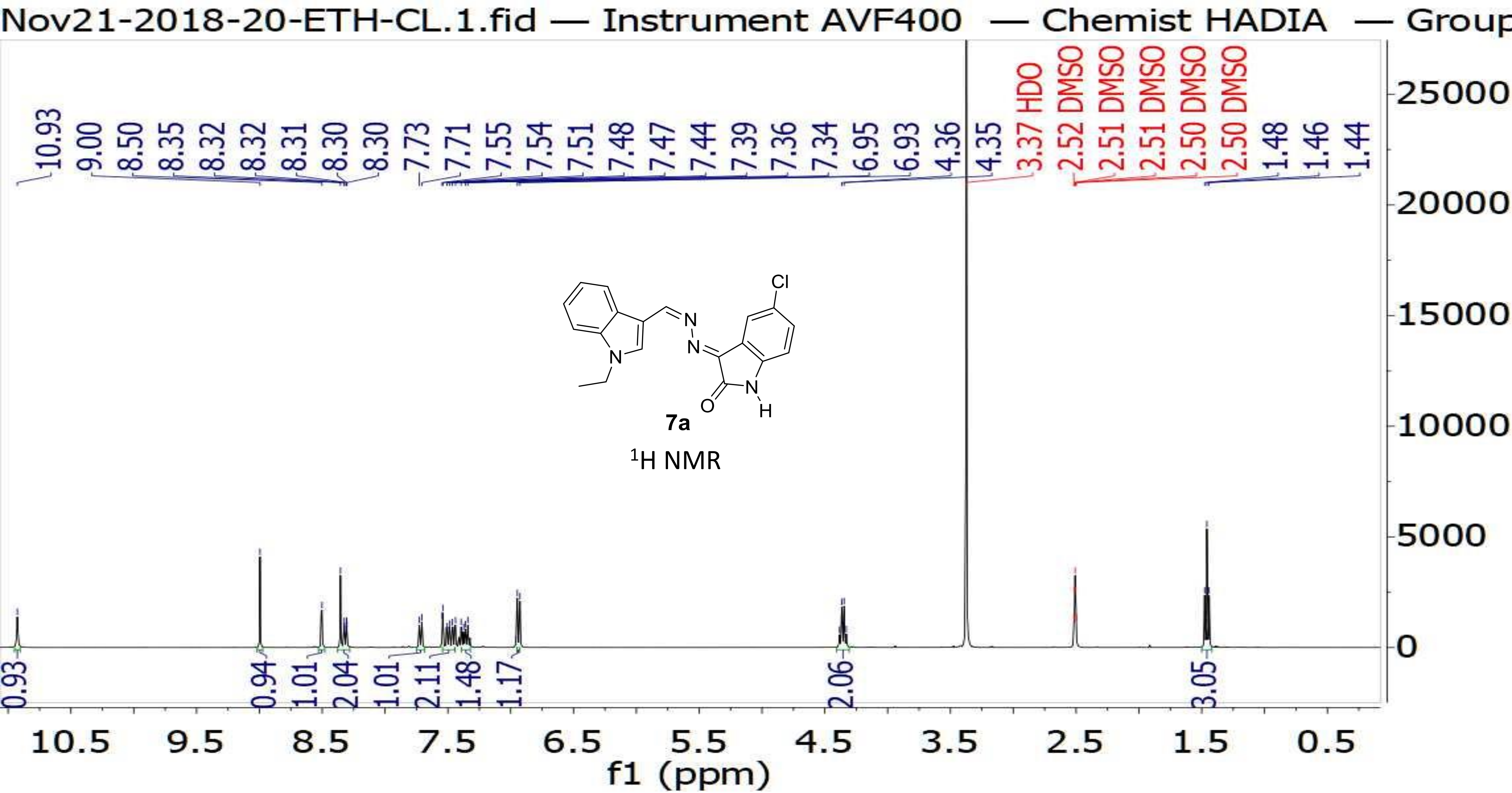
Nov21-2018-20-ETH-CL.1.fid — Instrument AVF400 — Chemist HADIA — Group
10.93 9.00 8.50 8.35 8.32 8.32 8.31 8.30 8.30 7.73 7.71 7.55 7.54 7.51 7.48 7.47 7.44 7.39 7.36 7.34 6.95 6.93 4.36 4.35
3.37 HDO 2.52 DMSO 2.51 DMSO 2.51 DMSO 2.50 DMSO 2.50 DMSO
1.48 1.46 1.44
Cl
N
N
N
N
O
H
7a
1H NMR
0.93 0.94 1.01 2.04 1.01 2.11 1.48 1.17 2.06 3.05
25000 20000 15000 10000 5000 0
10.5 9.5 8.5 7.5 6.5 5.5 4.5 3.5 2.5 1.5 0.5
f1 (ppm)

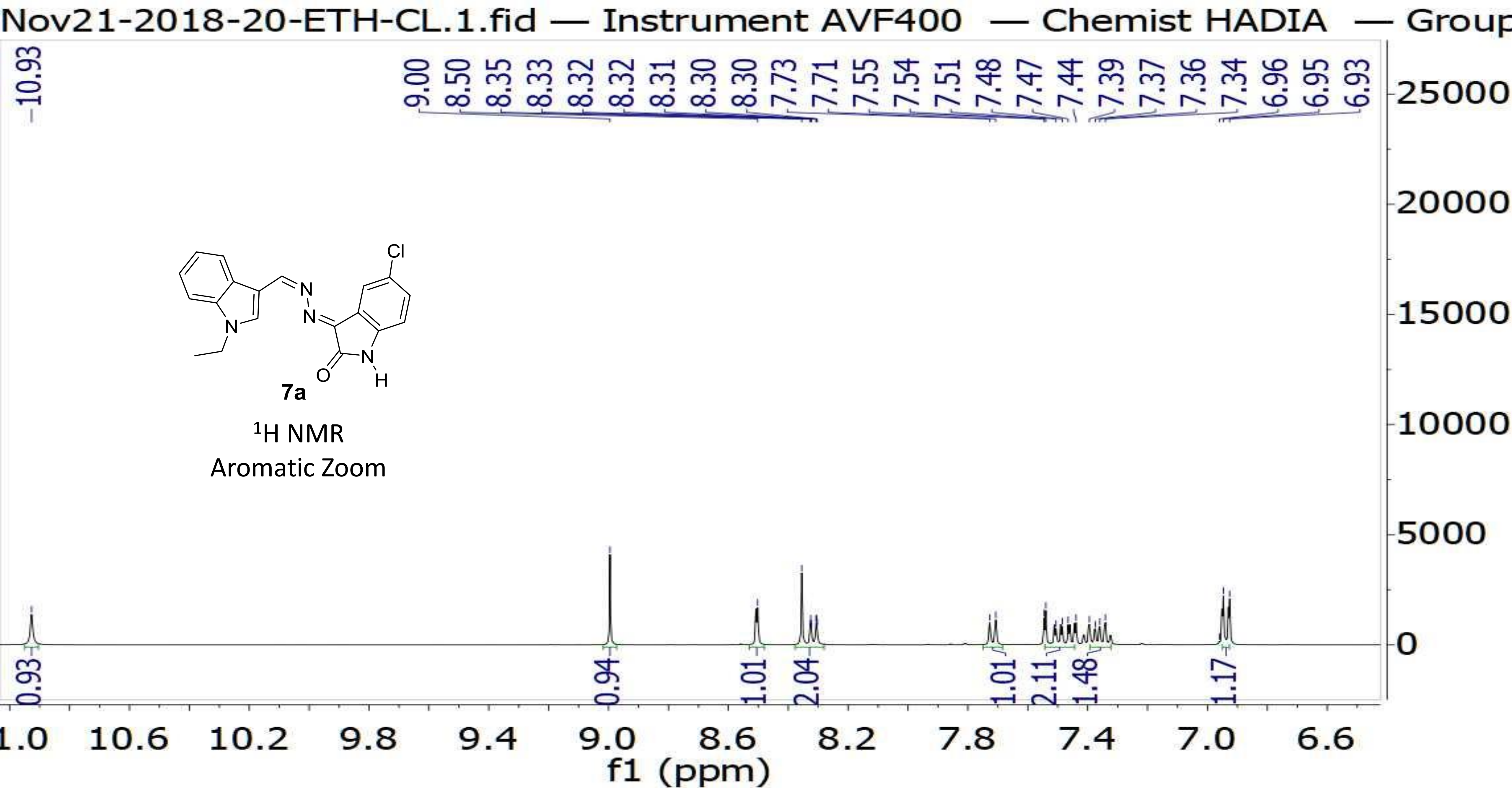
Nov21-2018-20-ETH-CL.1.fid — Instrument AVF400 — Chemist HADIA — Group
10.93
9.00 8.50 8.35 8.33 8.32 8.32 8.31 8.30 8.30 7.73 7.71 7.55 7.54 7.51 7.48 7.47 7.44 7.39 7.37 7.36 7.34 6.96 6.95 6.93
Cl
N
N
N
N
O
H
7a
1H NMR
Aromatic Zoom
25000
20000
15000
10000
5000
0
0.93 0.94 1.01 2.04 1.01 2.11 1.48 1.17
1.0 10.6 10.2 9.8 9.4 9.0 8.6 8.2 7.8 7.4 7.0 6.6
f1 (ppm)

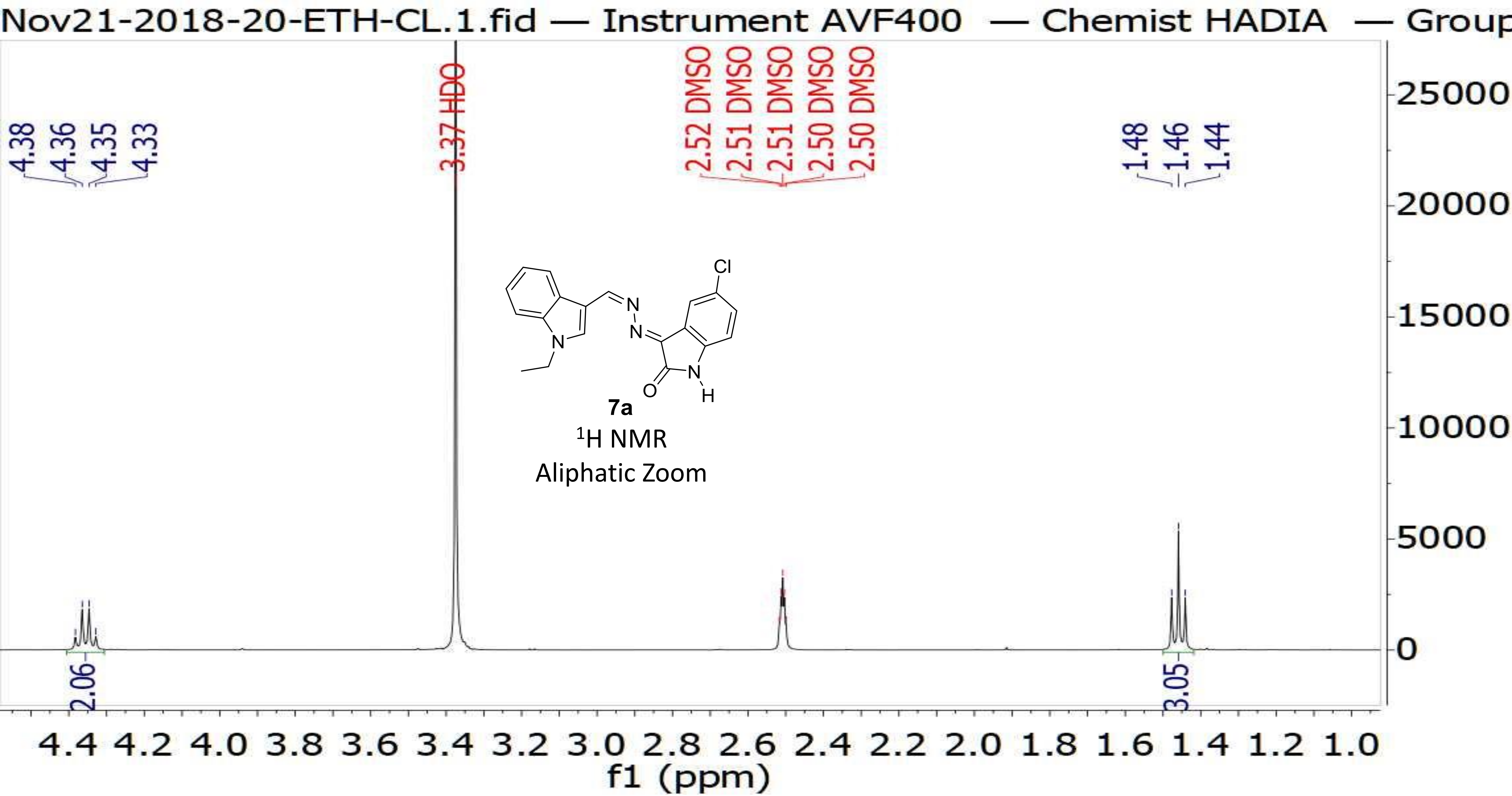

Nov21-2018-20-ETH-CL.1.fid — Instrument AVF400 — Chemist HADIA — Group
4.38
4.36
4.35
4.33
3.37 HDO
2.52 DMSO
2.51 DMSO
2.51 DMSO
2.50 DMSO
2.50 DMSO
1.48
1.46
1.44
Cl
N
N
N
N
O
H
7a
1H NMR
Aliphatic Zoom
25000
20000
15000
10000
5000
0
2.06
3.05
4.4 4.2 4.0 3.8 3.6 3.4 3.2 3.0 2.8 2.6 2.4 2.2 2.0 1.8 1.6 1.4 1.2 1.0
f1 (ppm)

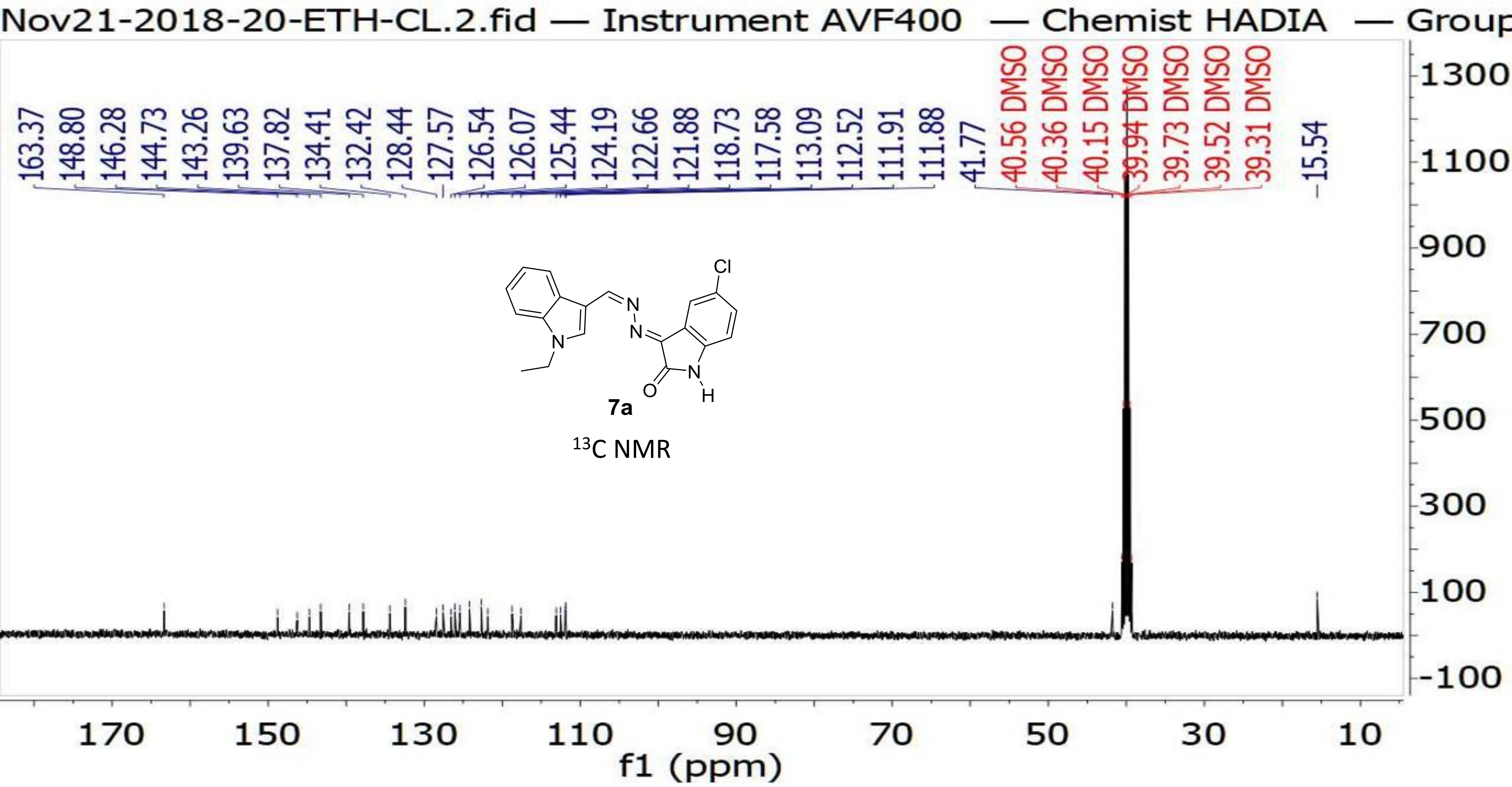
Nov21-2018-20-ETH-CL.2.fid — Instrument AVF400 — Chemist HADIA — Grou
163.37
148.80
146.28
144.73
143.26
139.63
137.82
134.41
132.42
128.44
127.57
126.54
126.07
125.44
124.19
122.66
121.88
118.73
117.58
113.09
112.52
111.91
111.88
41.77
40.56 DMSO
40.36 DMSO
40.15 DMSO
39.94 DMSO
39.73 DMSO
39.52 DMSO
39.31 DMSO
15.54
Cl
N
N
N
N
O
H
7a
13C NMR
1300
1100
900
700
500
300
100
-100
170
150
130
110
90
70
50
30
10
f1 (ppm)

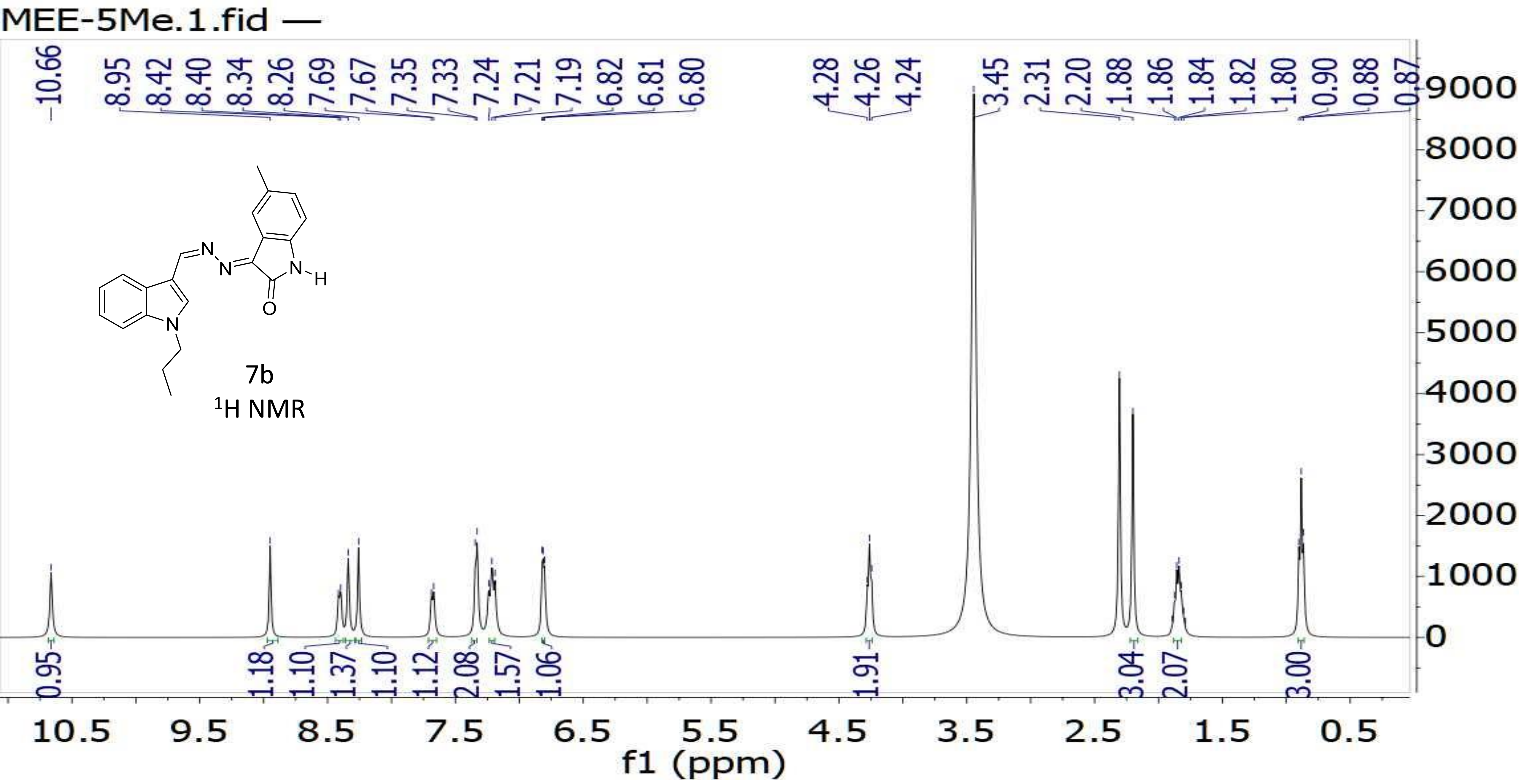
MEE-5Me.1.fid —
10.66
8.95 8.42 8.40 8.34 8.26 7.69 7.67 7.35 7.33 7.24 7.21 7.19 6.82 6.81 6.80
4.28 4.26 4.24
3.45
2.31 2.20 1.88 1.86 1.84 1.82 1.80 0.90 0.88 0.87
N
N
N
H
O
N
7b
1H NMR
9000
8000
7000
6000
5000
4000
3000
2000
1000
0
0.95 1.18 1.10 1.37 1.10 1.12 2.08 1.57 1.06 1.91 3.04 2.07 3.00
10.5 9.5 8.5 7.5 6.5 5.5 4.5 3.5 2.5 1.5 0.5
f1 (ppm)

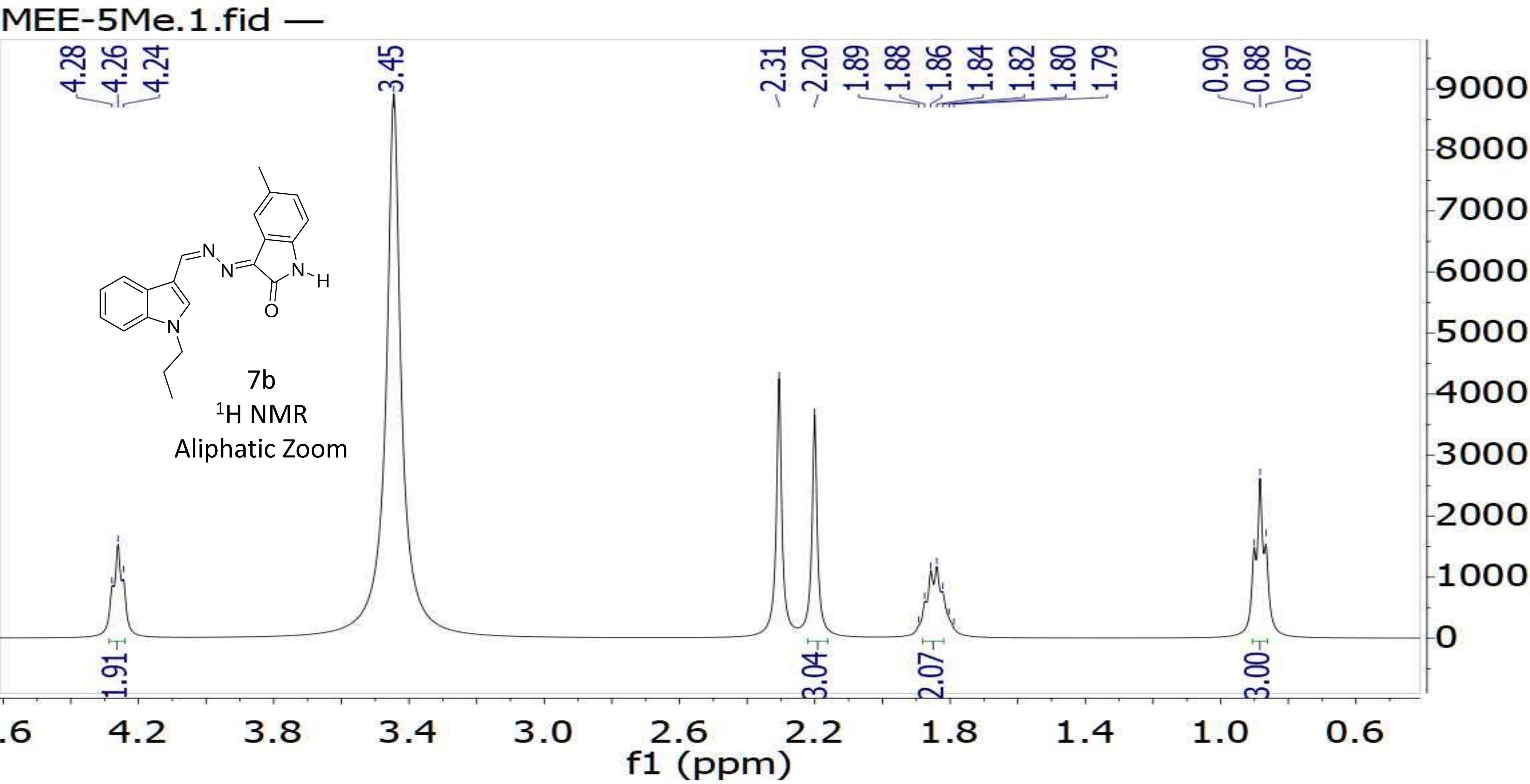
MEE-5Me.1.fid
4.28 4.26 4.24
3.45
2.31 2.20 1.89 1.88 1.86 1.84 1.82 1.80 1.79
0.90 0.88 0.87
7b
1H NMR
Aliphatic Zoom
1.91
3.04
2.07
3.00
9000 8000 7000 6000 5000 4000 3000 2000 1000 0
.6 4.2 3.8 3.4 3.0 2.6 2.2 1.8 1.4 1.0 0.6
f1 (ppm)

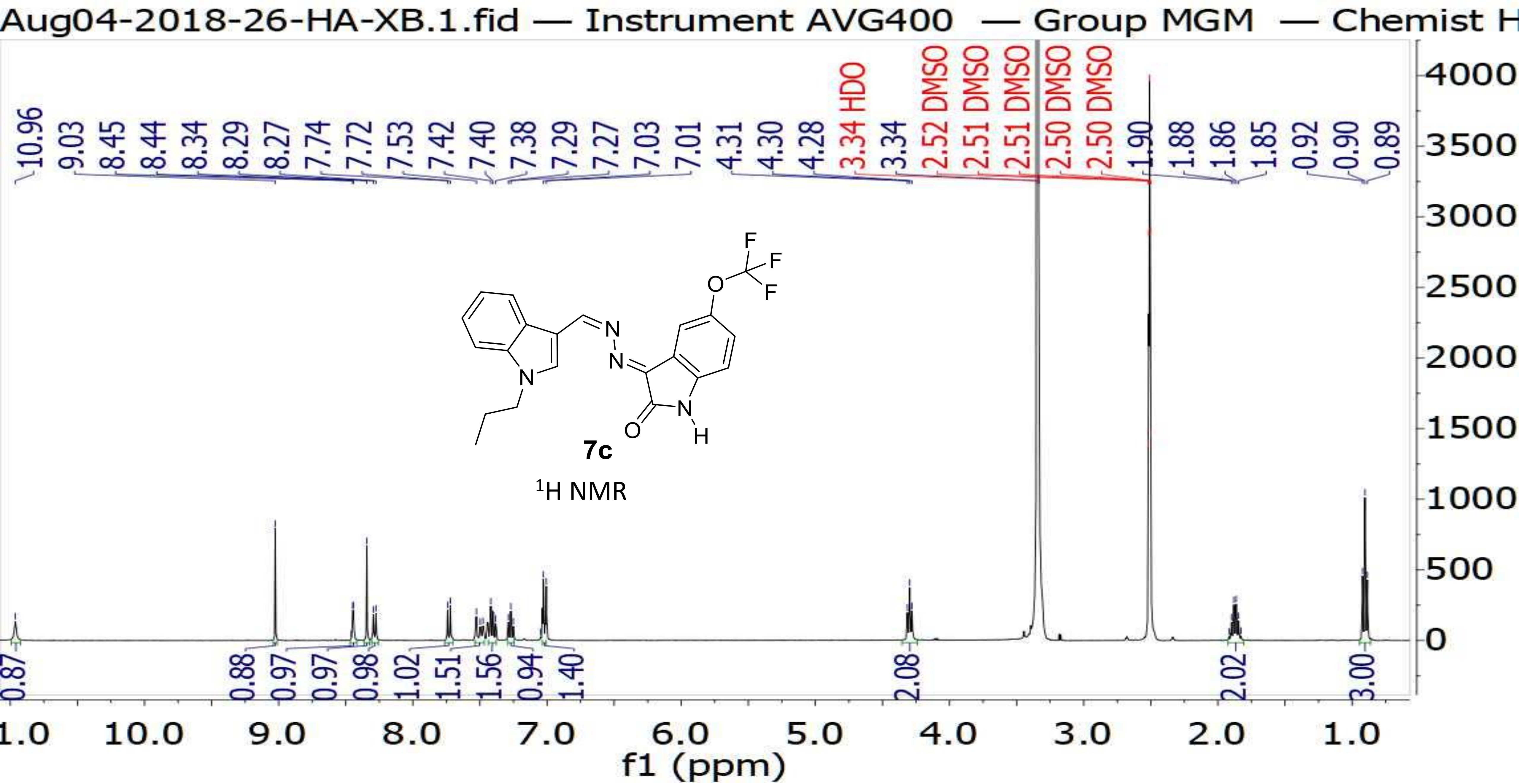
Aug04-2018-26-HA-XB.1.fid — Instrument AVG400 — Group MGM — Chemist H
10.96 9.03 8.45 8.44 8.34 8.29 8.27 7.74 7.72 7.53 7.42 7.40 7.38 7.29 7.27 7.03 7.01 4.31 4.30 4.28
3.34 HDO 3.34 2.52 DMSO 2.51 DMSO 2.51 DMSO 2.50 DMSO 2.50 DMSO
1.90 1.88 1.86 1.85 0.92 0.90 0.89
F
F
F
O
N
N
N
N
O
H
7c
^{1}H NMR
0.87 0.88 0.97 0.97 0.98 1.02 1.51 1.56 0.94 1.40 2.08 2.02 3.00
4000 3500 3000 2500 2000 1500 1000 500 0
1.0 10.0 9.0 8.0 7.0 6.0 5.0 4.0 3.0 2.0 1.0
f1 (ppm)

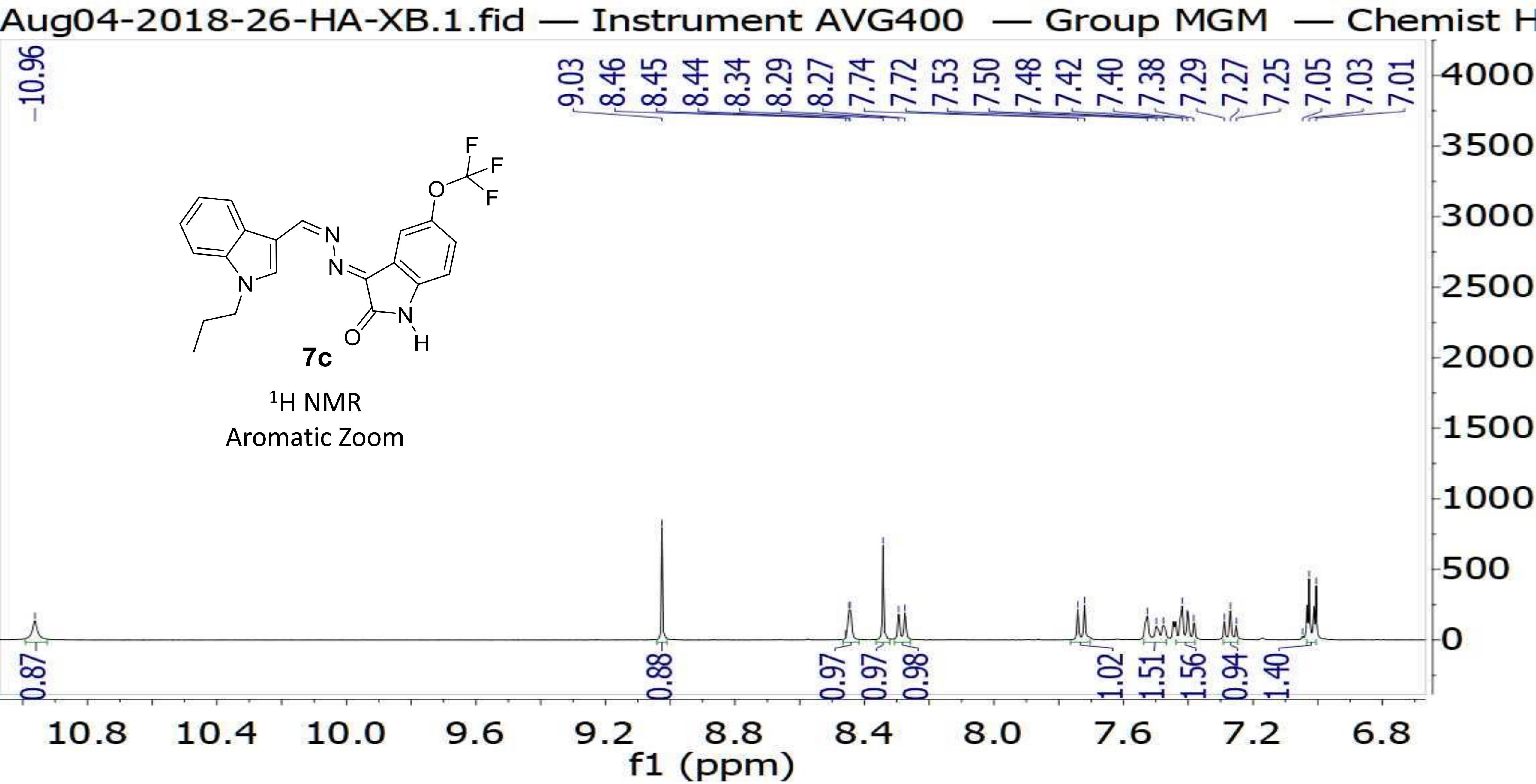
Aug04-2018-26-HA-XB.1.fid — Instrument AVG400 — Group MGM — Chemist H
10.96
9.03 8.46 8.45 8.44 8.34 8.29 8.27 7.74 7.72 7.53 7.50 7.48 7.42 7.40 7.38 7.29 7.27 7.25 7.05 7.03 7.01
F
F
F
O
N
N
N
N
O
H
7c
¹H NMR
Aromatic Zoom
4000
3500
3000
2500
2000
1500
1000
500
0
0.87
0.88
0.97
0.97
0.98
1.02
1.51
1.56
0.94
1.40
10.8 10.4 10.0 9.6 9.2 8.8 8.4 8.0 7.6 7.2 6.8
f1 (ppm)

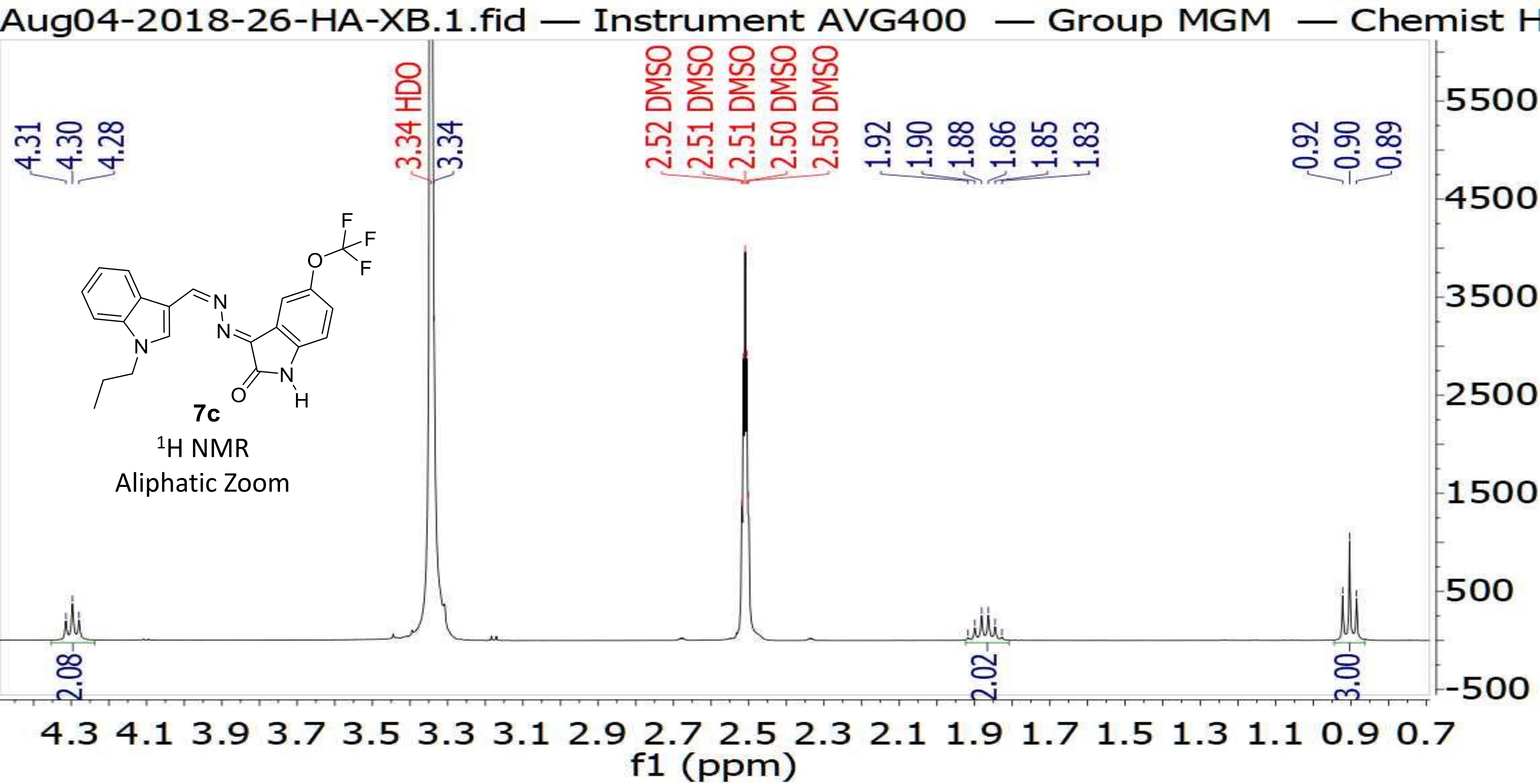

Aug04-2018-26-HA-XB.1.fid — Instrument AVG400 — Group MGM — Chemist H
4.31 4.30 4.28
3.34 HDO
3.34
2.52 DMSO 2.51 DMSO 2.51 DMSO 2.50 DMSO 2.50 DMSO
1.92 1.90 1.88 1.86 1.85 1.83
0.92 0.90 0.89
2.08
2.02
3.00
7c
1H NMR
Aliphatic Zoom
5500
4500
3500
2500
1500
500
-500
4.3 4.1 3.9 3.7 3.5 3.3 3.1 2.9 2.7 2.5 2.3 2.1 1.9 1.7 1.5 1.3 1.1 0.9 0.7
f1 (ppm)

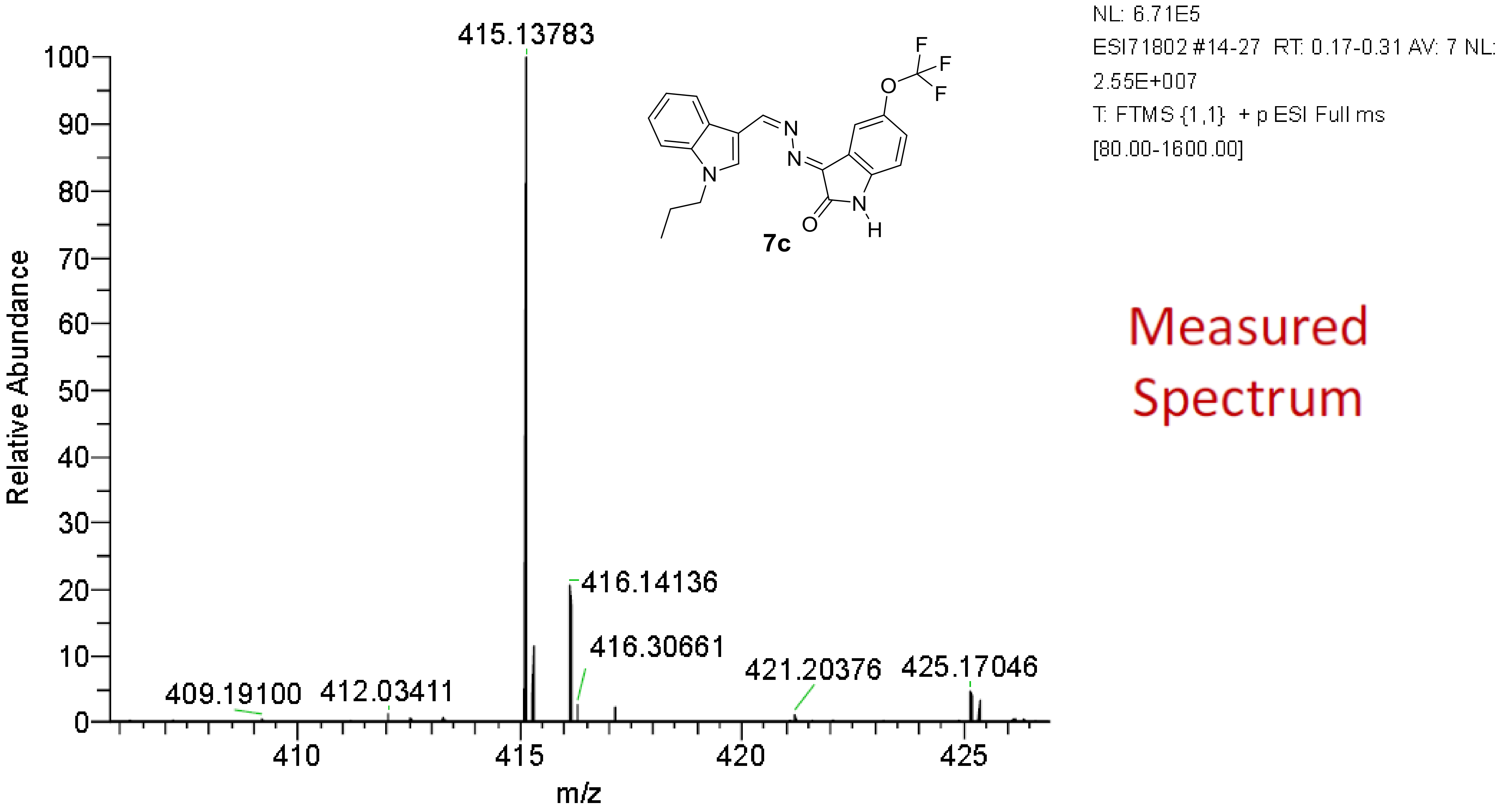

415.13783
100
90
80
70
60
50
40
30
20
10
0
Relative Abundance
416.14136
416.30661
409.19100
412.03411
421.20376
425.17046
410
415
420
425
m/z
N
N
N
O
F
F
F
N
H
O
7c
NL: 6.71E5
ESI71802 #14-27 RT: 0.17-0.31 AV: 7 NL: 2.55E+007
T: FTMS {1,1} + p ESI Full ms [80.00-1600.00]
Measured Spectrum

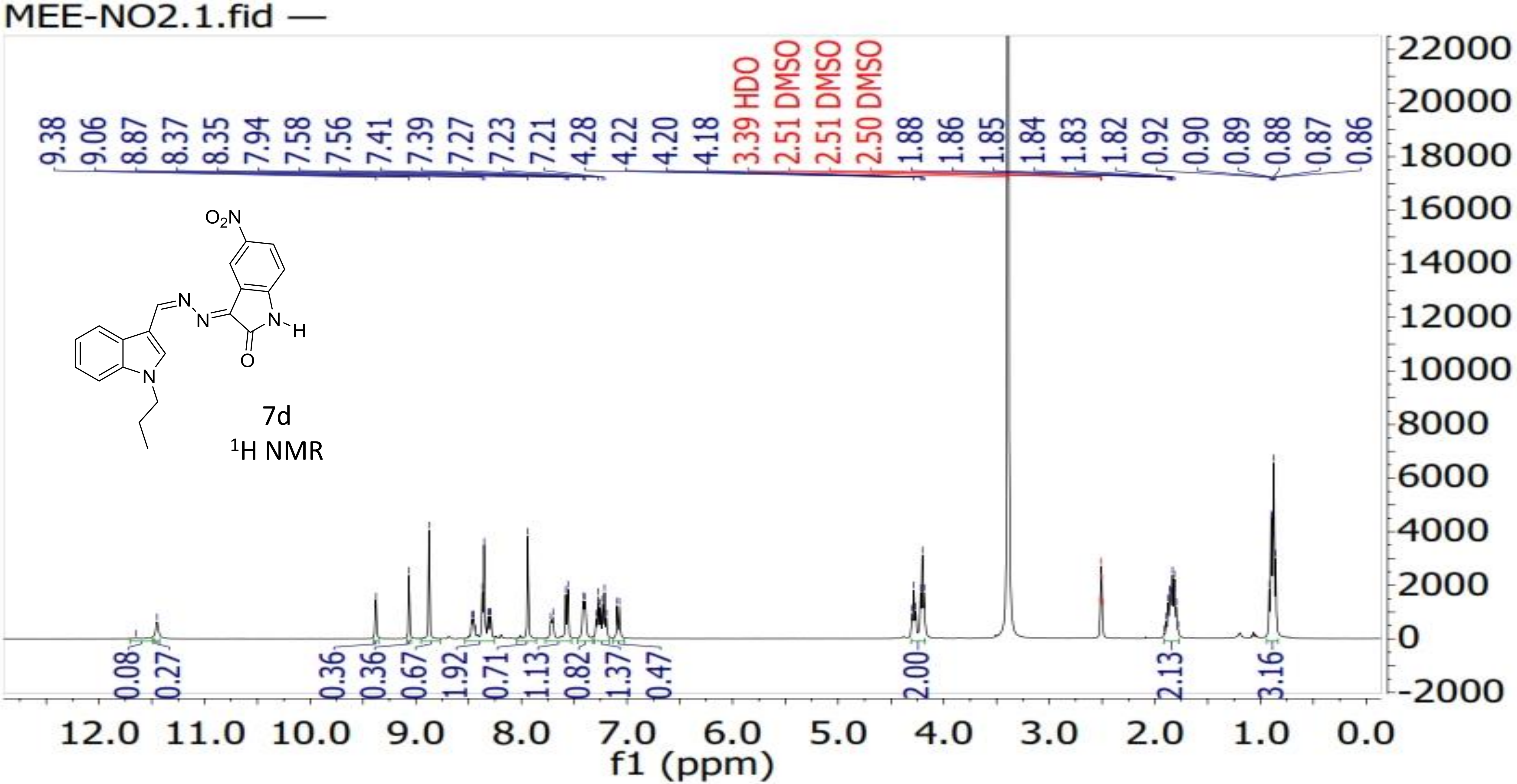
MEE-NO2.1.fid
9.38 9.06 8.87 8.37 8.35 7.94 7.58 7.56 7.41 7.39 7.27 7.23 7.21 4.28 4.22 4.20 4.18
3.39 HDO 2.51 DMSO 2.51 DMSO 2.50 DMSO
1.88 1.86 1.85 1.84 1.83 1.82 0.92 0.90 0.89 0.88 0.87 0.86
O_2N
N
N
N
H
O
N
7d
^1H NMR
0.08 0.27 0.36 0.36 0.67 1.92 0.71 1.13 0.82 1.37 0.47 2.00 2.13 3.16
f1 (ppm)

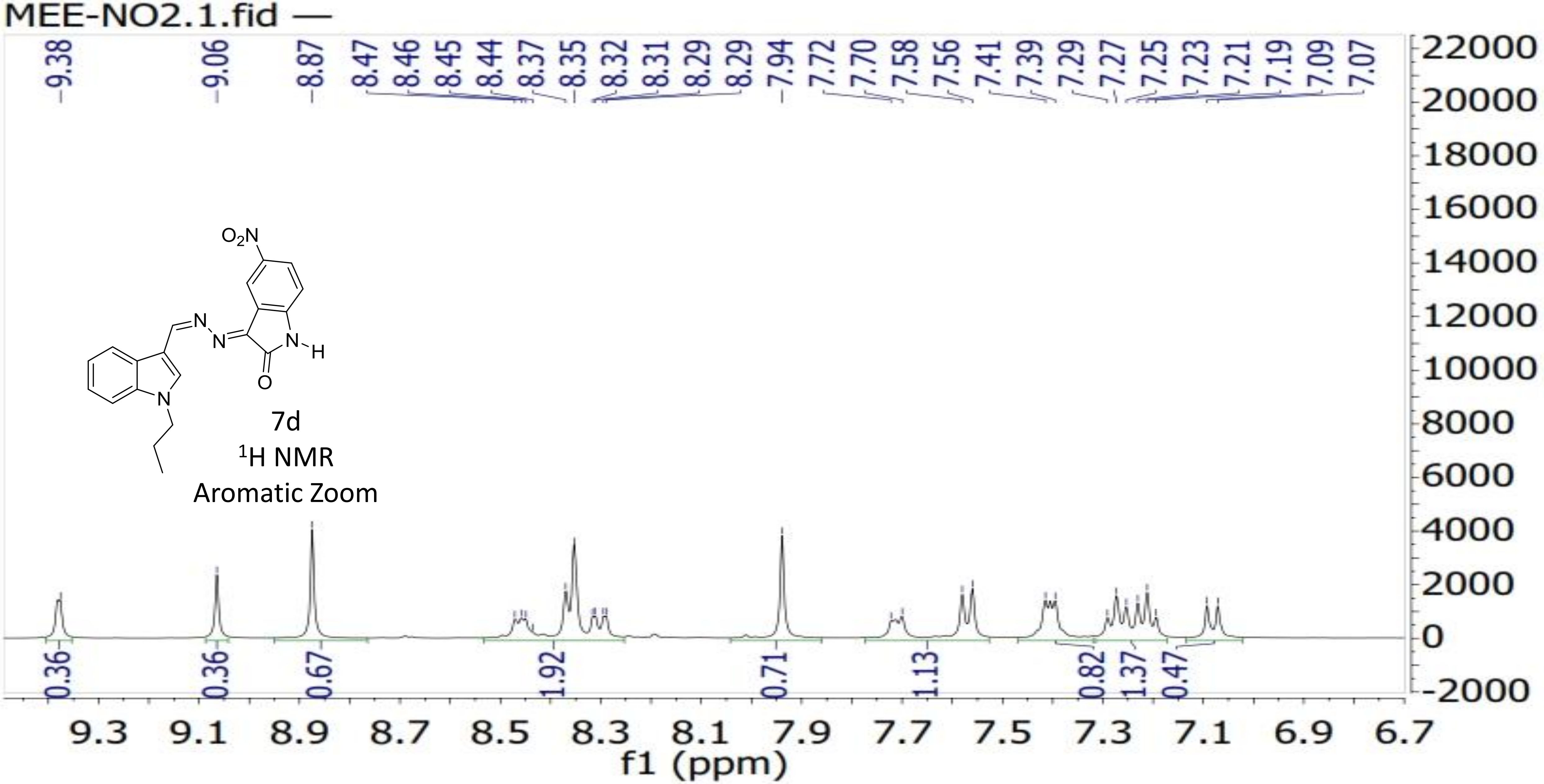
MEE-NO2.1.fid
9.38
9.06
8.87
8.47
8.46
8.45
8.44
8.37
8.35
8.32
8.31
8.29
8.29
7.94
7.72
7.70
7.58
7.56
7.41
7.39
7.29
7.27
7.25
7.23
7.21
7.19
7.09
7.07
O_2N
N
N
N
H
O
N
7d
^{1}H NMR
Aromatic Zoom
0.36
0.36
0.67
1.92
0.71
1.13
0.82
1.37
0.47
f1 (ppm)

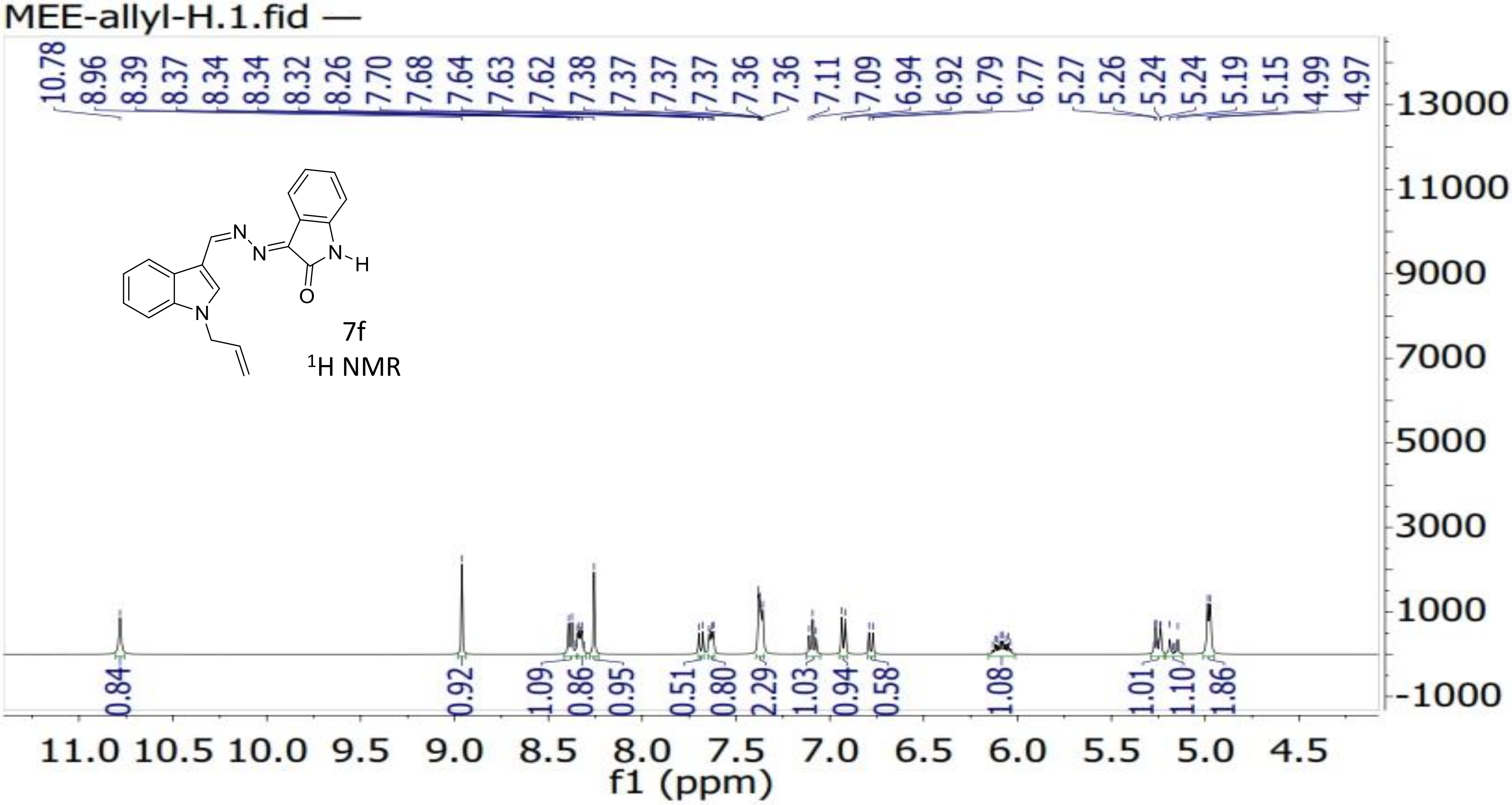
MEE-allyl-H.1.fid —
10.78 8.96 8.39 8.37 8.34 8.34 8.32 8.26 7.70 7.68 7.64 7.63 7.62 7.38 7.37 7.37 7.37 7.36 7.36 7.11 7.09 6.94 6.92 6.79 6.77 5.27 5.26 5.24 5.24 5.19 5.15 4.99 4.97
N
N
N
H
O
N
7f
^{1}H NMR
0.84 0.92 1.09 0.86 0.95 0.51 0.80 2.29 1.03 0.94 0.58 1.08 1.01 1.10 1.86
13000 11000 9000 7000 5000 3000 1000 -1000
11.0 10.5 10.0 9.5 9.0 8.5 8.0 7.5 7.0 6.5 6.0 5.5 5.0 4.5
f1 (ppm)

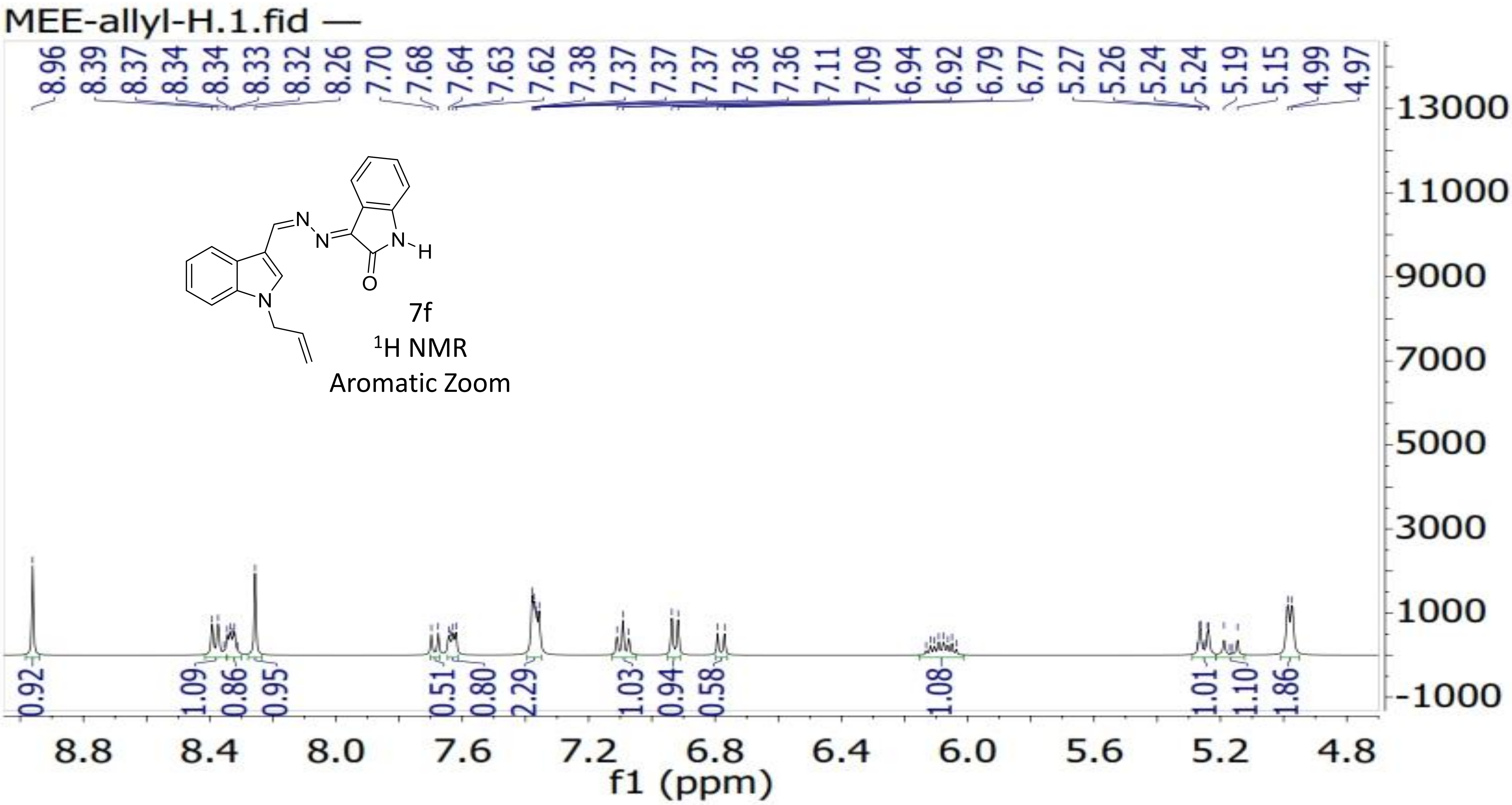
MEE-allyl-H.1.fid —
8.96 8.39 8.37 8.34 8.34 8.33 8.32 8.26 7.70 7.68 7.64 7.63 7.62 7.38 7.37 7.37 7.37 7.36 7.36 7.11 7.09 6.94 6.92 6.79 6.77 5.27 5.26 5.24 5.24 5.19 5.15 4.99 4.97
N
N
N
H
O
N
7f
1H NMR
Aromatic Zoom
0.92 1.09 0.86 0.95 0.51 0.80 2.29 1.03 0.94 0.58 1.08 1.01 1.10 1.86
8.8 8.4 8.0 7.6 7.2 6.8 6.4 6.0 5.6 5.2 4.8
f1 (ppm)
13000
11000
9000
7000
5000
3000
1000
-1000

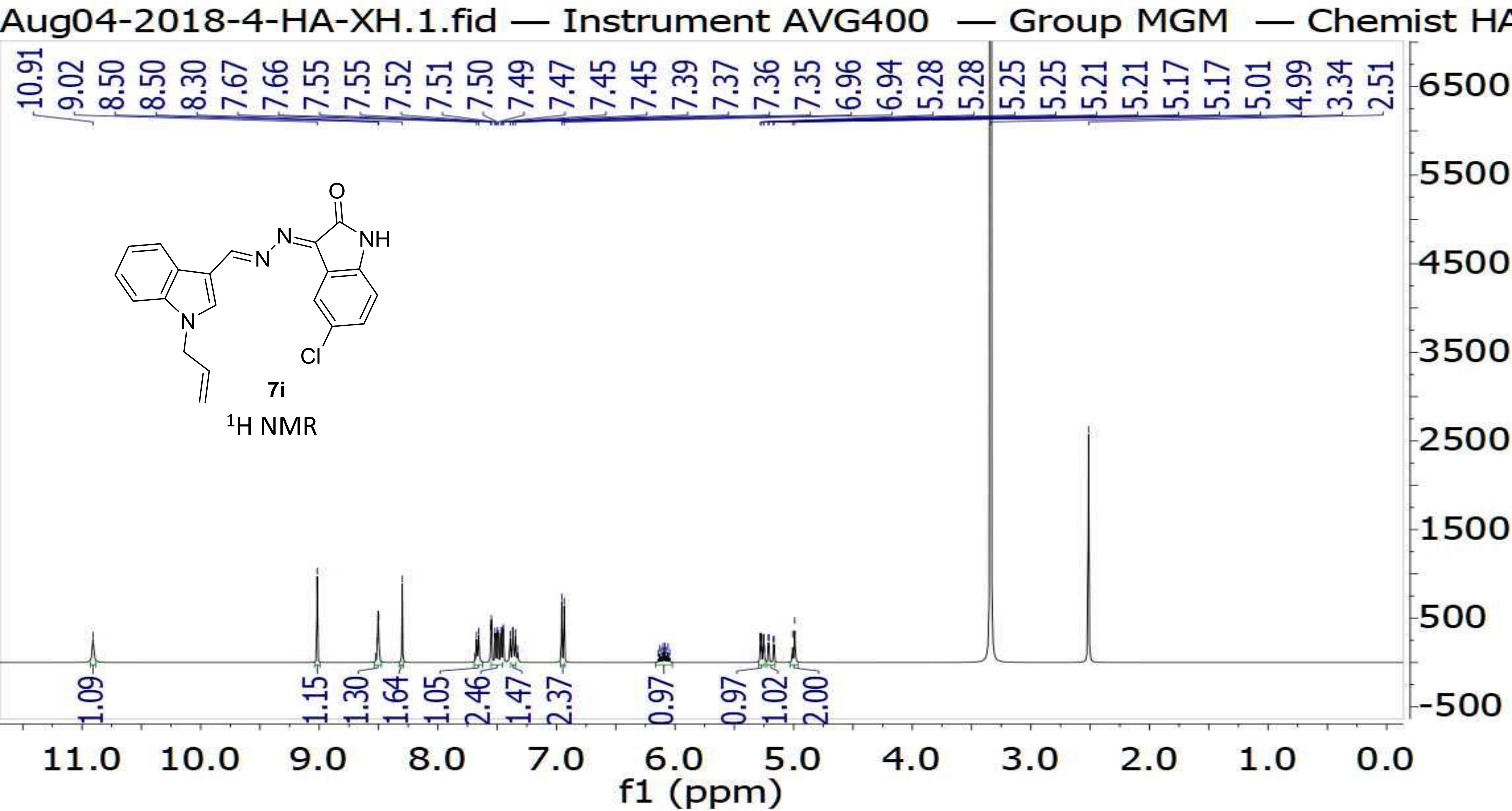

Aug04-2018-4-HA-XH.1.fid — Instrument AVG400 — Group MGM — Chemist HA
10.91 9.02 8.50 8.50 8.30 7.67 7.66 7.55 7.55 7.52 7.51 7.50 7.49 7.47 7.45 7.45 7.39 7.37 7.36 7.35 6.96 6.94 5.28 5.28 5.25 5.25 5.21 5.21 5.17 5.17 5.01 4.99 3.34 2.51
O
NH
N
N
N
Cl
7i
1H NMR
1.09 1.15 1.30 1.64 1.05 2.46 1.47 2.37 0.97 0.97 1.02 2.00
6500
5500
4500
3500
2500
1500
500
-500
11.0 10.0 9.0 8.0 7.0 6.0 5.0 4.0 3.0 2.0 1.0 0.0
f1 (ppm)

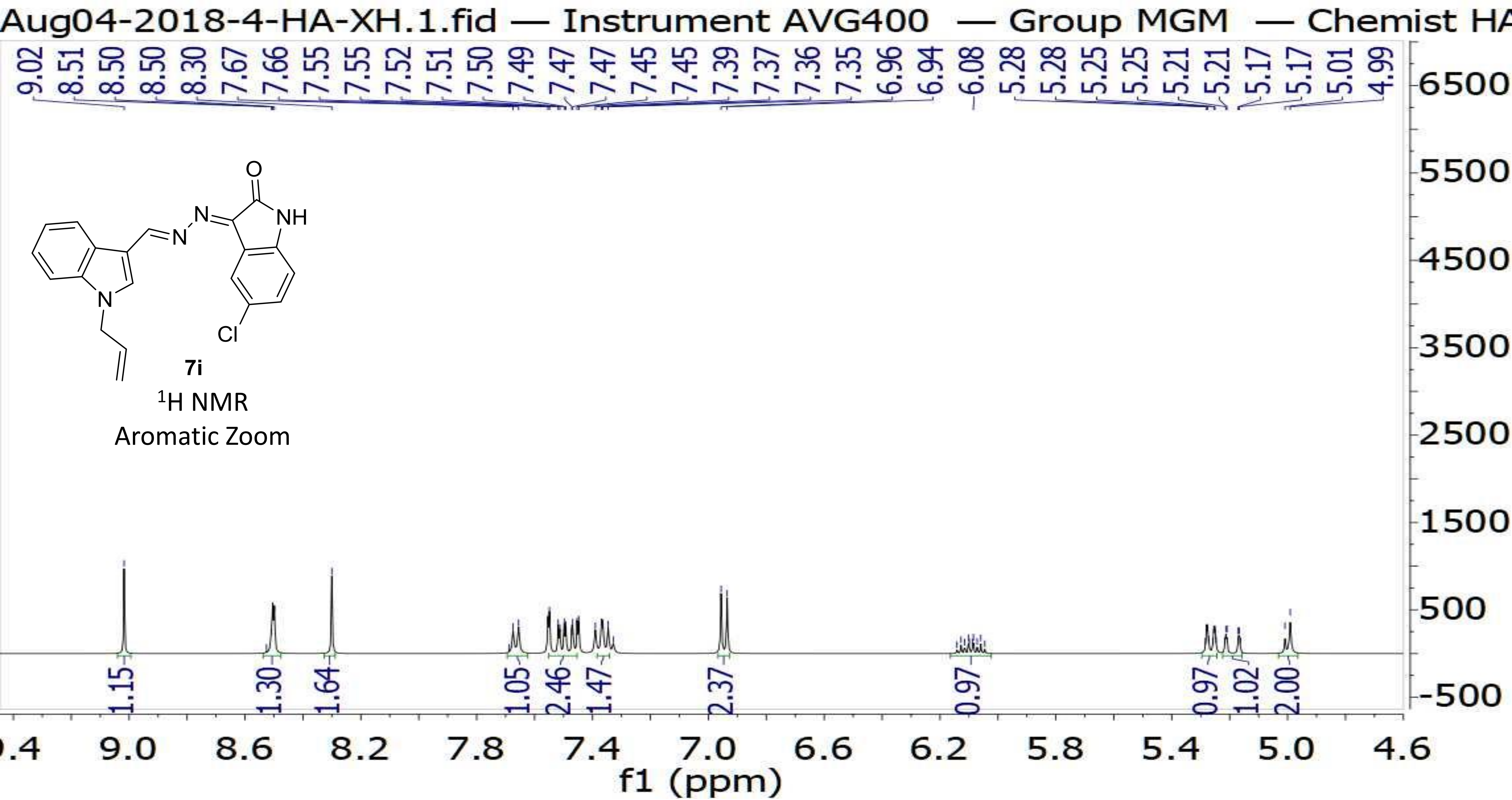
Aug04-2018-4-HA-XH.1.fid — Instrument AVG400 — Group MGM — Chemist HA
9.02 8.51 8.50 8.50 8.30 7.67 7.66 7.55 7.55 7.52 7.51 7.50 7.49 7.47 7.47 7.45 7.45 7.39 7.37 7.36 7.35 6.96 6.94 6.08 5.28 5.28 5.25 5.25 5.21 5.21 5.17 5.17 5.01 4.99
O
N
NH
N
N
Cl
7i
^{1}H NMR
Aromatic Zoom
1.15 1.30 1.64 1.05 2.46 1.47 2.37 0.97 0.97 1.02 2.00
6500
5500
4500
3500
2500
1500
500
-500
9.0 8.6 8.2 7.8 7.4 7.0 6.6 6.2 5.8 5.4 5.0 4.6
f1 (ppm)

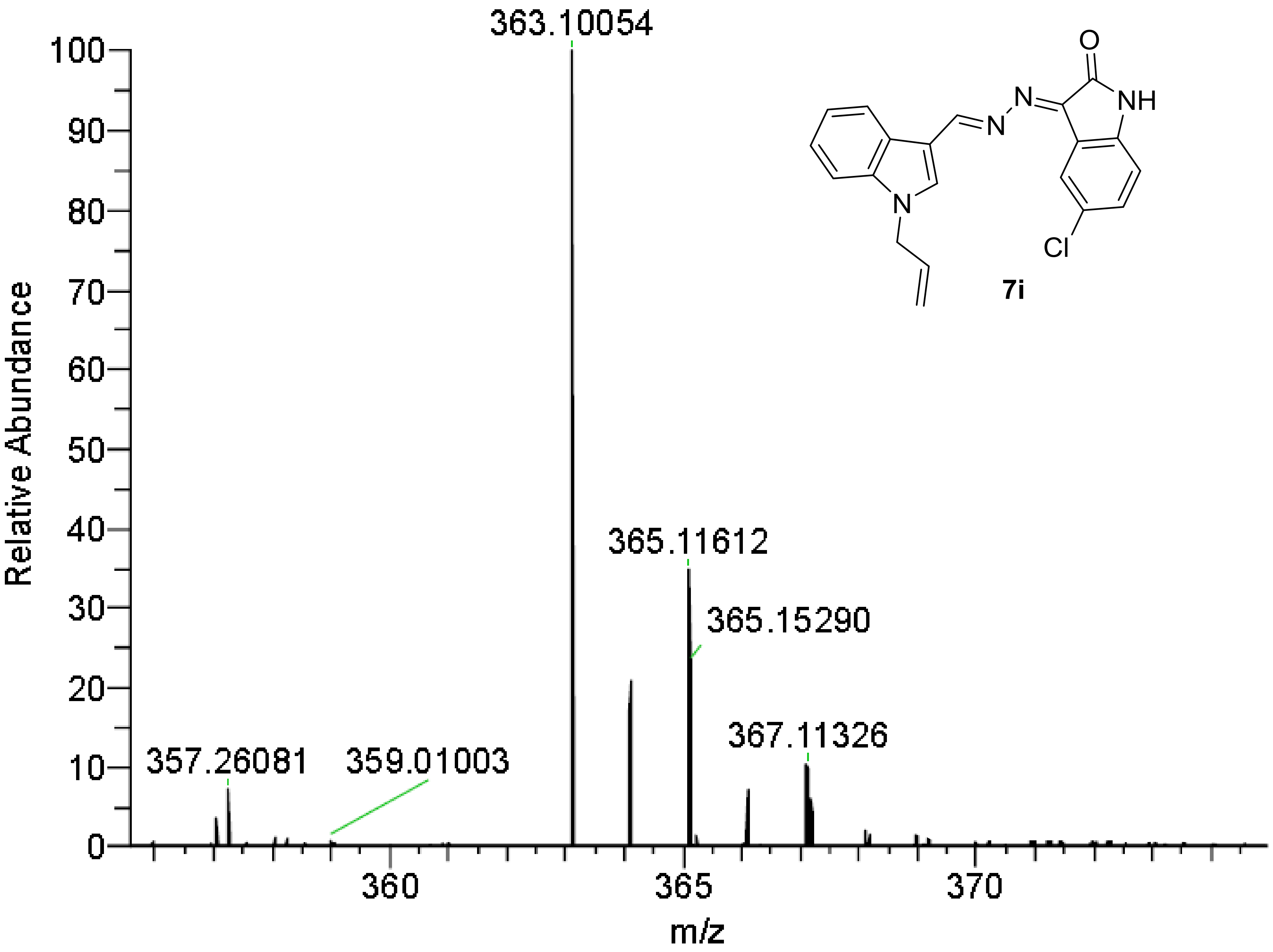

NL: 7.58E5
ESI71804 #14-27 RT: 0.17-0.31 AV: 7 NL: 2.72E+007
T: FTMS {1,1} + p ESI Full ms [80.00-1600.00]

Measured Spectrum

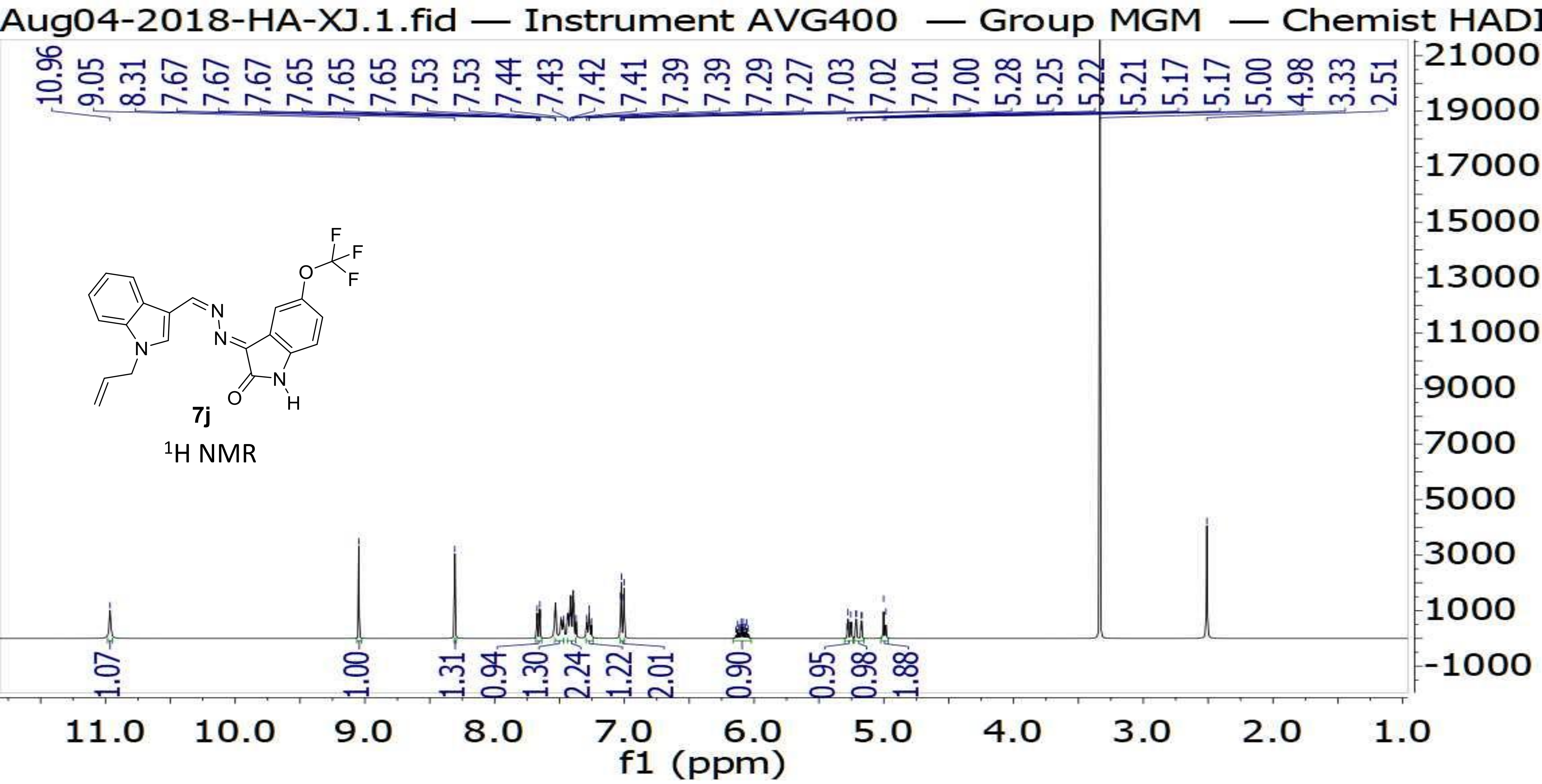

Aug04-2018-HA-XJ.1.fid — Instrument AVG400 — Group MGM — Chemist HADI
10.96 9.05 8.31 7.67 7.67 7.67 7.65 7.65 7.65 7.53 7.53 7.44 7.43 7.42 7.41 7.39 7.39 7.29 7.27 7.03 7.02 7.01 7.00 5.28 5.25 5.22 5.21 5.17 5.17 5.00 4.98 3.33 2.51
F
F
F
O
N
N
N
N
H
O
7j
1H NMR
1.07 1.00 1.31 0.94 1.30 2.24 1.22 2.01 0.90 0.95 0.98 1.88
21000 19000 17000 15000 13000 11000 9000 7000 5000 3000 1000 -1000
11.0 10.0 9.0 8.0 7.0 6.0 5.0 4.0 3.0 2.0 1.0
f1 (ppm)

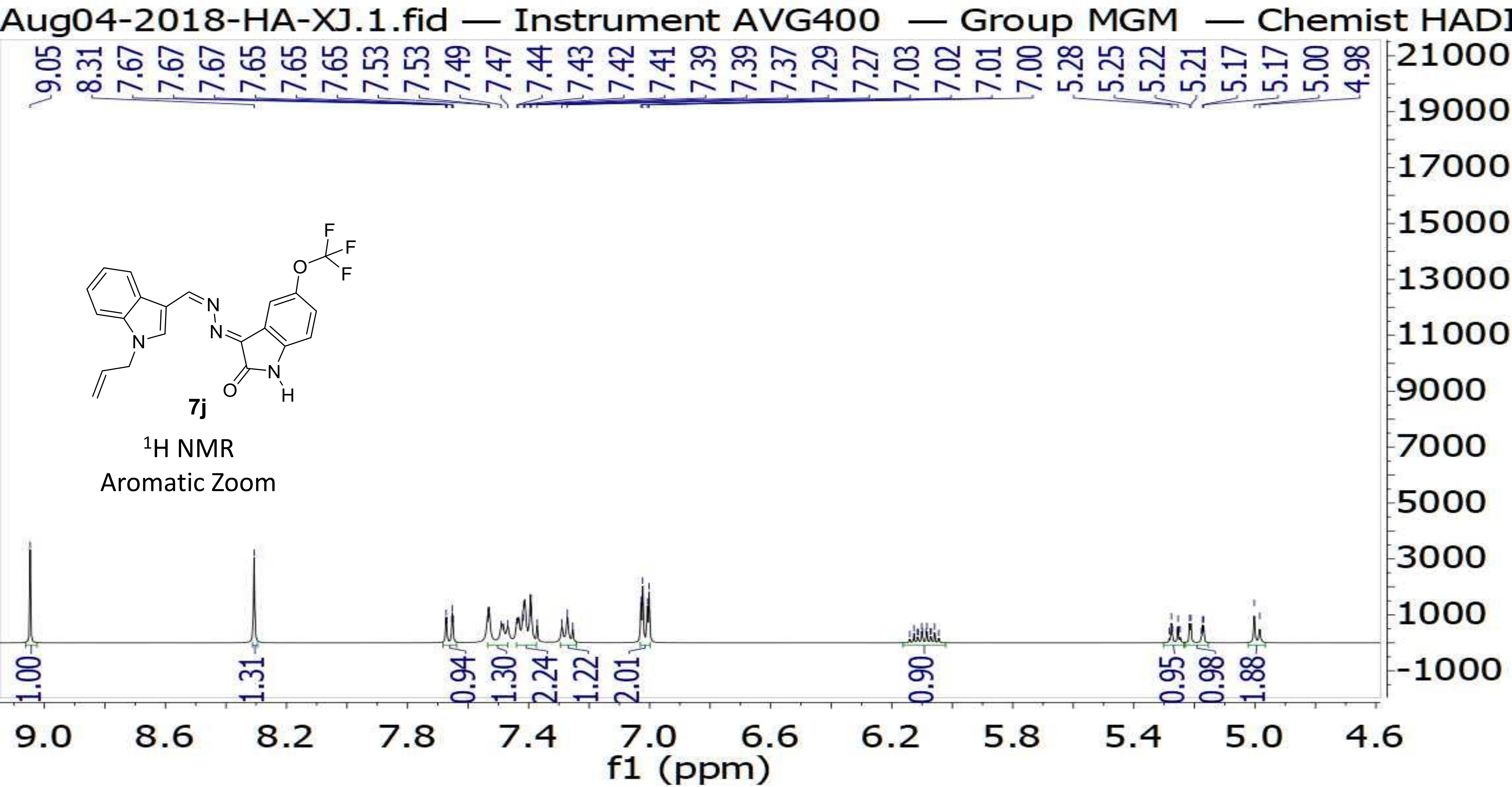
Aug04-2018-HA-XJ.1.fid — Instrument AVG400 — Group MGM — Chemist HADI
9.05 8.31 7.67 7.67 7.67 7.65 7.65 7.65 7.53 7.53 7.49 7.47 7.44 7.43 7.42 7.41 7.39 7.39 7.37 7.29 7.27 7.03 7.02 7.01 7.00 5.28 5.25 5.22 5.21 5.17 5.17 5.00 4.98
F
F
F
O
N
N
N
O
N
H
7j
1H NMR
Aromatic Zoom
1.00 1.31 0.94 1.30 2.24 1.22 2.01 0.90 0.95 0.98 1.88
21000 19000 17000 15000 13000 11000 9000 7000 5000 3000 1000 -1000
9.0 8.6 8.2 7.8 7.4 7.0 6.6 6.2 5.8 5.4 5.0 4.6
f1 (ppm)

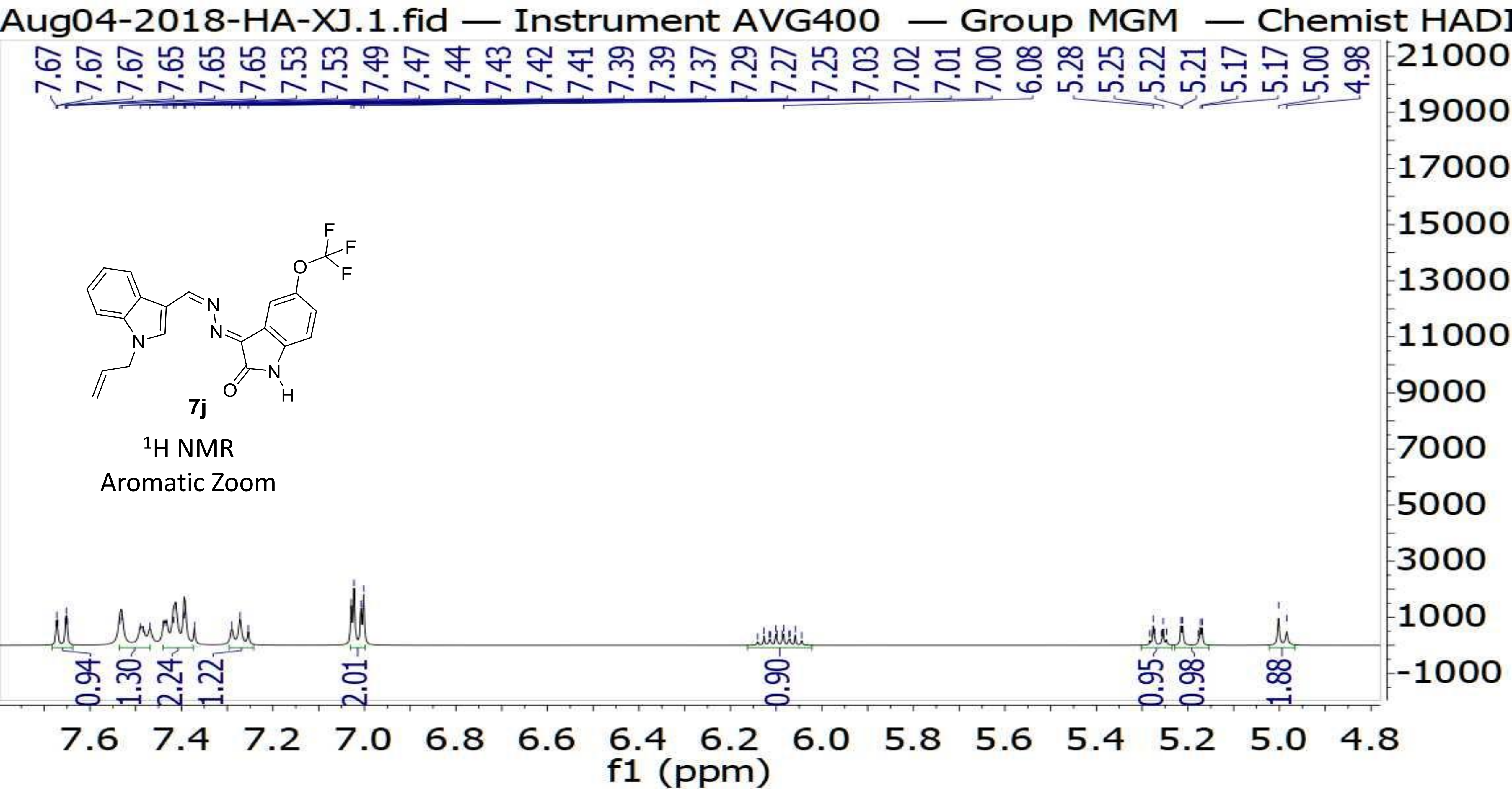
Aug04-2018-HA-XJ.1.fid — Instrument AVG400 — Group MGM — Chemist HADI
7.67 7.67 7.67 7.65 7.65 7.65 7.53 7.53 7.49 7.47 7.44 7.43 7.42 7.41 7.39 7.39 7.37 7.29 7.27 7.25 7.03 7.02 7.01 7.00 6.08 5.28 5.25 5.22 5.21 5.17 5.17 5.00 4.98
F
F
F
O
N
N
N
O
N
H
7j
1H NMR
Aromatic Zoom
0.94 1.30 2.24 1.22 2.01 0.90 0.95 0.98 1.88
21000 19000 17000 15000 13000 11000 9000 7000 5000 3000 1000 -1000
7.6 7.4 7.2 7.0 6.8 6.6 6.4 6.2 6.0 5.8 5.6 5.4 5.2 5.0 4.8
f1 (ppm)

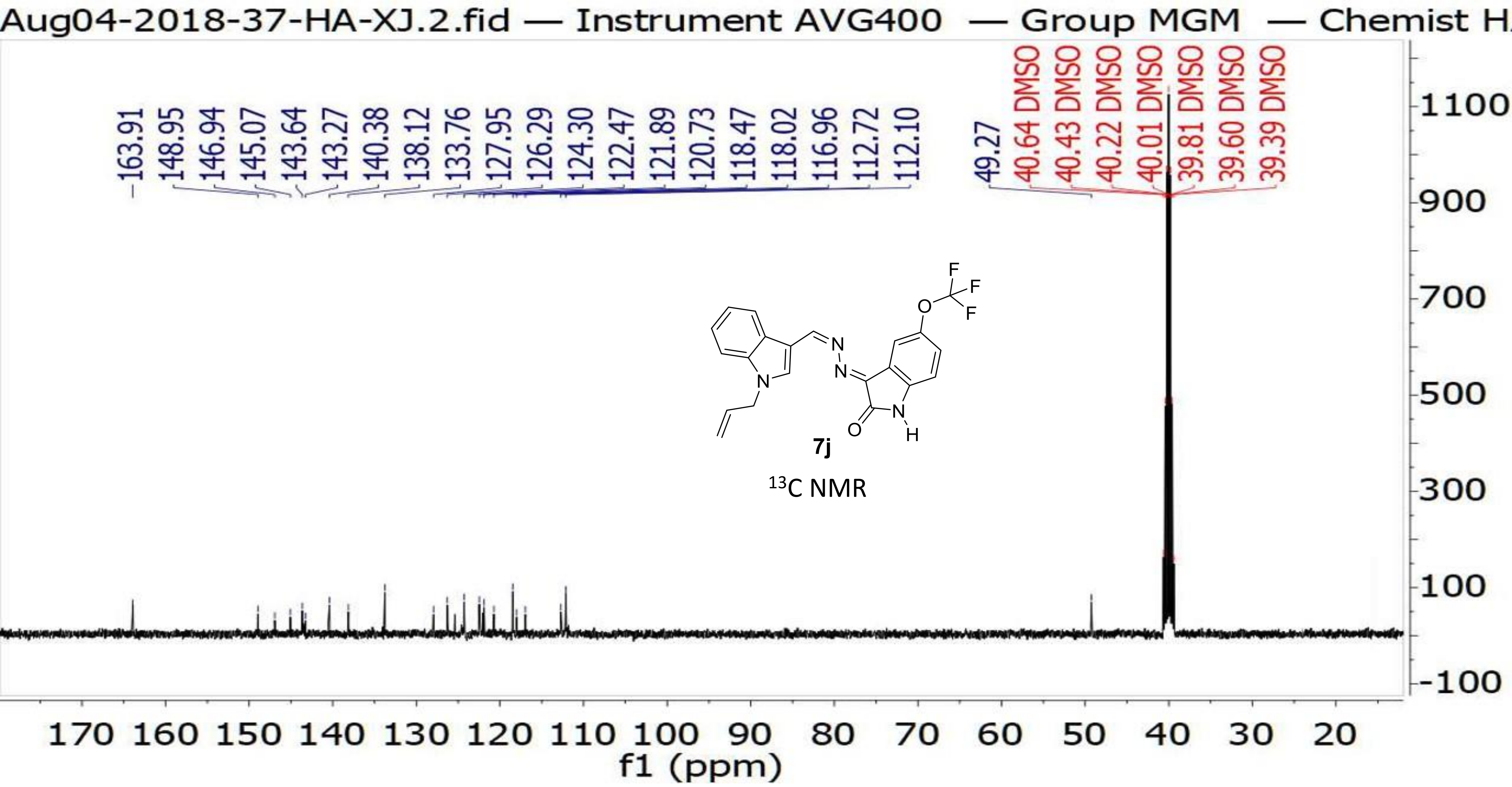
Aug04-2018-37-HA-XJ.2.fid — Instrument AVG400 — Group MGM — Chemist H.
163.91
148.95
146.94
145.07
143.64
143.27
140.38
138.12
133.76
127.95
126.29
124.30
122.47
121.89
120.73
118.47
118.02
116.96
112.72
112.10
49.27
40.64 DMSO
40.43 DMSO
40.22 DMSO
40.01 DMSO
39.81 DMSO
39.60 DMSO
39.39 DMSO
F
F
F
O
N
N
N
N
O
H
7j
13C NMR
f1 (ppm)

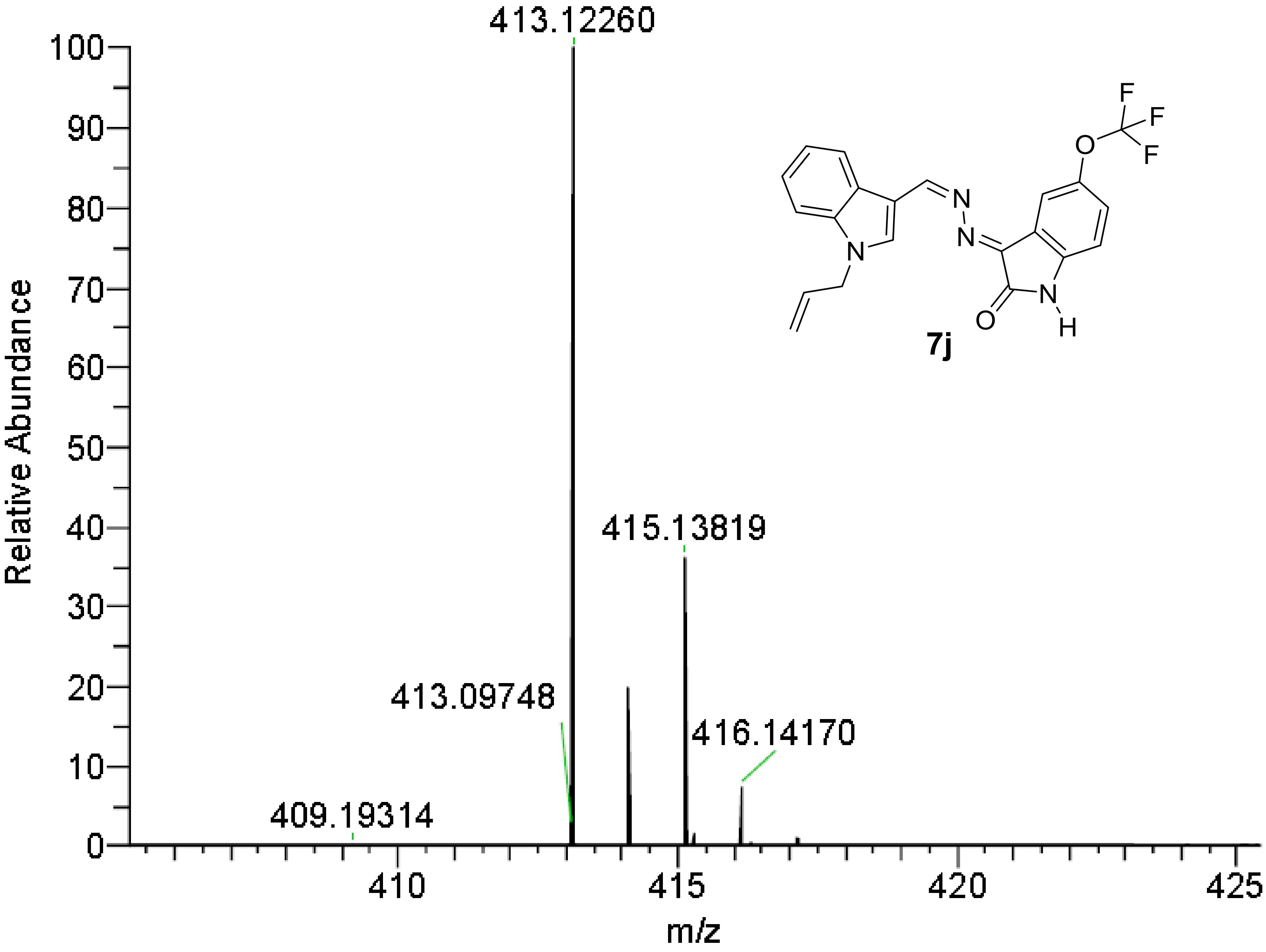

7j

NL: 2.54E6
ESI71803 #14-28 RT: 0.17-0.3 AV: 7 NL: 2.15E+007
T: FTMS {1,1} + p ESI Full ms [80.00-1600.00]

Measured Spectrum

Developmental Therapeutics Program

One Dose Mean Graph

**NSC:** D-795311 / 1	**Conc:** 1.00E-5 Molar	**Test Date:** Jan 17, 2017
**Experiment ID:** 1701OS42		**Report Date:** Feb 18, 2017

Panel/Cell Line	Growth Percent
**Leukemia**	
CCRF-CEM	-0.65
HL-60(TB)	-22.61
K-562	17.61
MOLT-4	-9.90
RPMI-8226	-24.86
SR	11.60
**Non-Small Cell Lung Cancer**	
A549/ATCC	63.93
EKVX	50.45
HOP-62	75.35
HOP-92	37.42
NCI-H226	92.04
NCI-H23	48.03
NCI-H322M	47.76
NCI-H460	32.08
NCI-H522	13.81
**Colon Cancer**	
COLO 205	-16.20
HCC-2998	46.95
HCT-116	39.85
HCT-15	23.04
HT29	17.08
KM12	59.92
SW-620	36.69
**CNS Cancer**	
SF-268	46.59
SF-295	71.12
SF-539	33.94
SNB-19	63.34
SNB-75	85.63
U251	39.96
**Melanoma**	
LOX IMVI	4.42
MALME-3M	51.68
M14	56.29
MDA-MB-435	-26.64
SK-MEL-2	35.06
SK-MEL-28	59.40
SK-MEL-5	49.07
UACC-257	58.80
UACC-62	40.39
**Ovarian Cancer**	
IGROV1	39.38
OVCAR-3	2.10
OVCAR-4	41.83
OVCAR-5	84.60
OVCAR-8	47.49
NCI/ADR-RES	76.32
SK-OV-3	88.36
**Renal Cancer**	
786-0	64.53
A498	57.21
ACHN	55.74
RXF 393	11.38
SN12C	49.93
TK-10	106.72
UO-31	60.00
**Prostate Cancer**	
PC-3	17.96
DU-145	66.43
**Breast Cancer**	
MCF7	8.92
MDA-MB-231/ATCC	34.35
HS 578T	74.02
BT-549	63.53
T-47D	39.59
MDA-MB-468	25.19
Mean	41.08
Delta	67.72
Range	133.36

Mean Growth Percent - Growth Percent

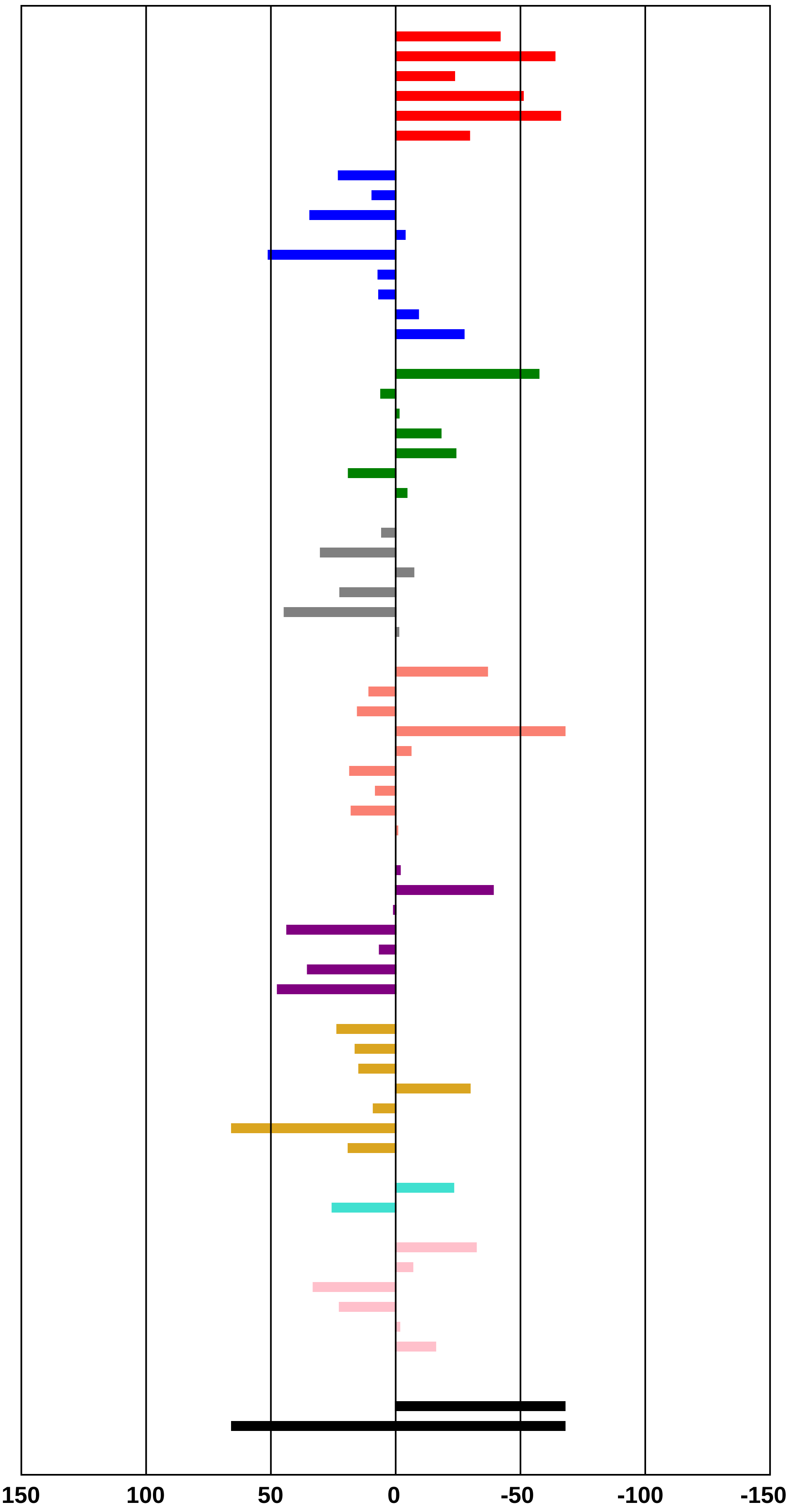

National Cancer Institute Developmental Therapeutics Program
Dose Response Curves

NSC: D - 795311 / 1	SSPL: 0YVT	EXP. ID: 1702NS56
Report Date: March 20, 2017	Test Date: February 13, 2017	

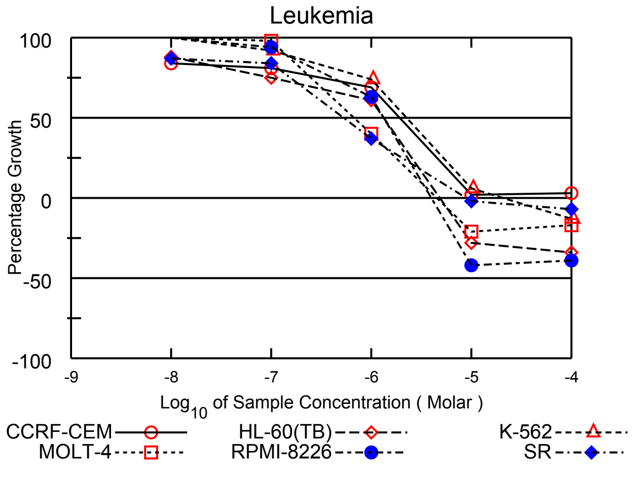

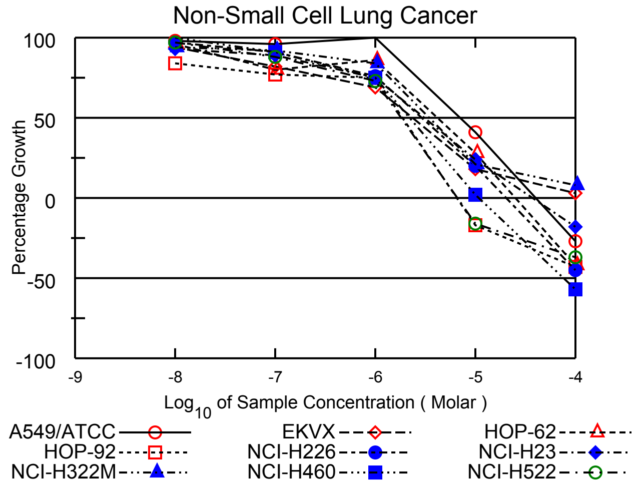

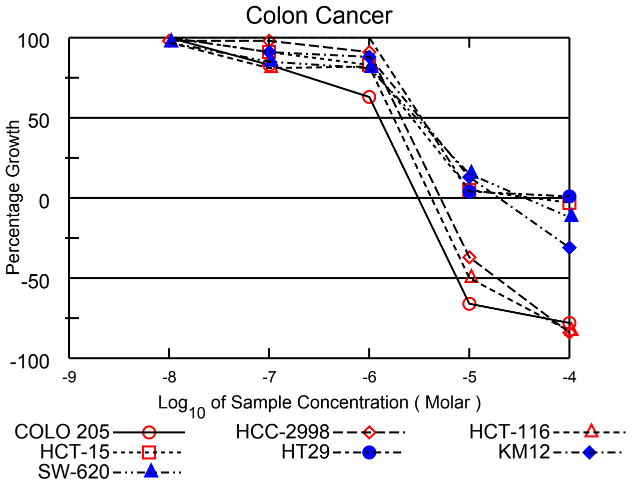

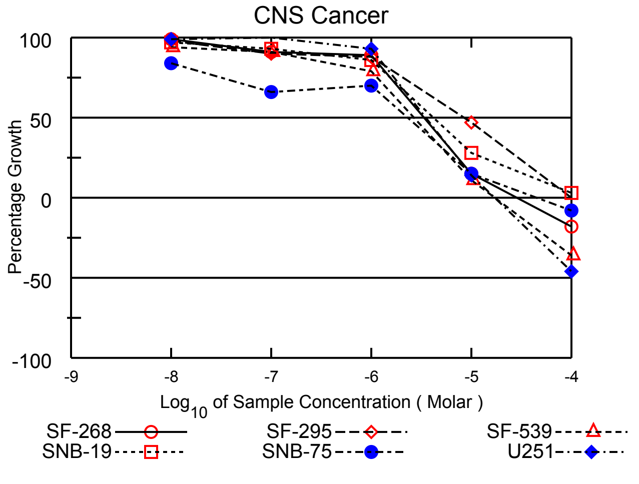

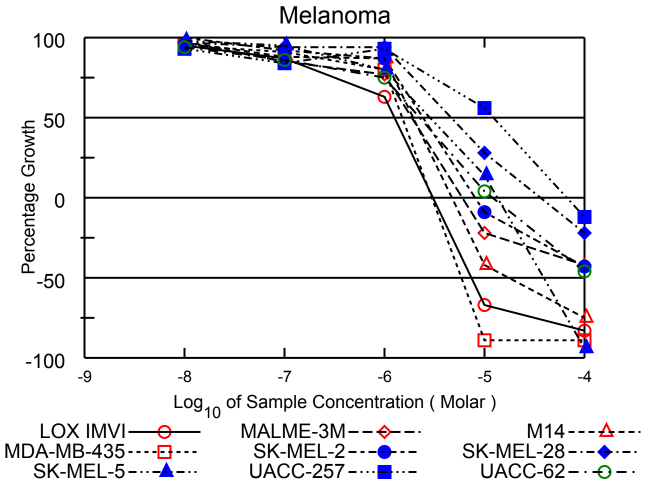

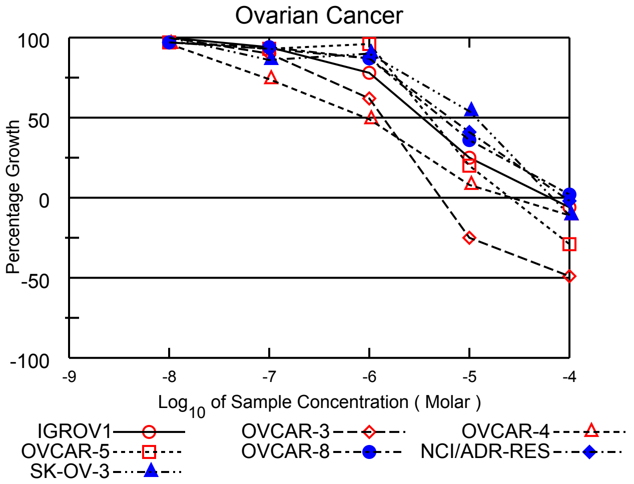

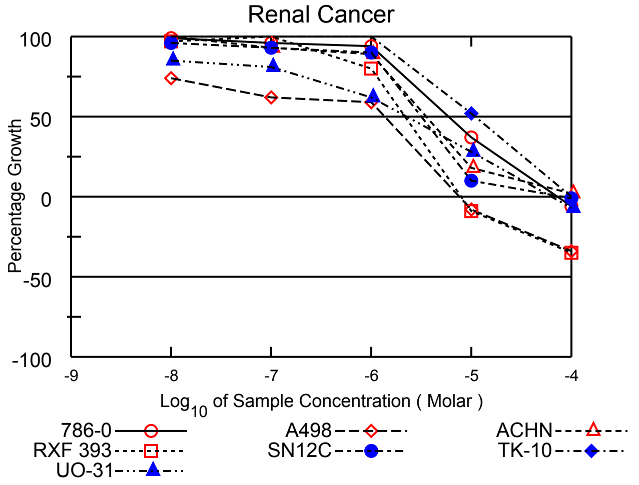

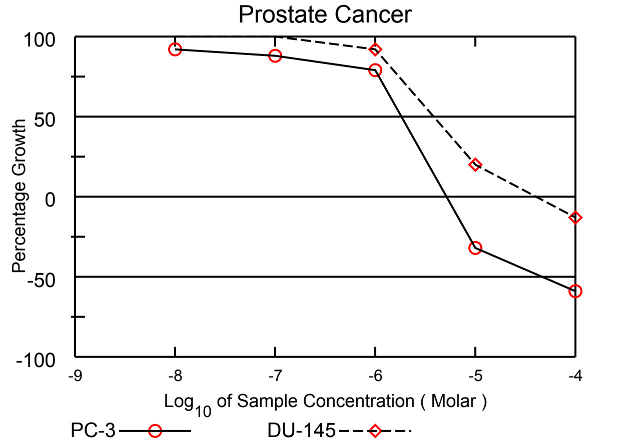

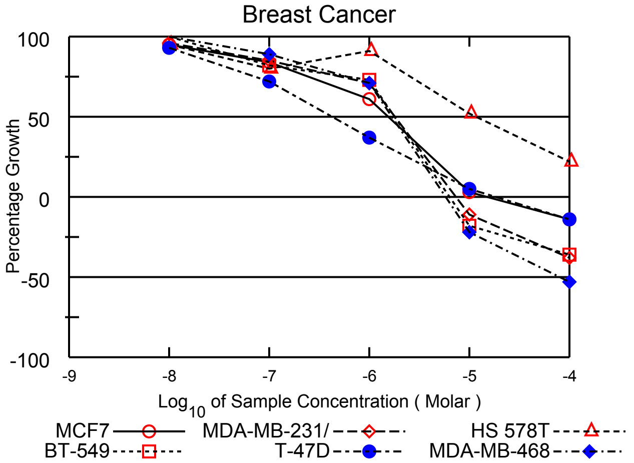

National Cancer Institute Developmental Therapeutics Program
In-Vitro Testing Results

NSC : D - 795311 / 1	Experiment ID : 1702NS56	Test Type : 08	Units : Molar
Report Date : March 20, 2017	Test Date : February 13, 2017	QNS :	MC :
COMI : Prop-Me	Stain Reagent : SRB Dual-Pass Related	SSPL : 0YVT	

Log10 Concentration

	Time		Mean Optical Densities					Percent Growth							
Panel/Cell Line	Zero	Ctrl	-8.0	-7.0	-6.0	-5.0	-4.0	-8.0	-7.0	-6.0	-5.0	-4.0	GI50	TGI	LC50
Leukemia															
CCRF-CEM	0.504	2.705	2.354	2.280	2.031	0.551	0.564	84	81	69	2	3	1.94E-6	> 1.00E-4	> 1.00E-4
HL-60(TB)	0.668	2.267	2.078	1.875	1.636	0.479	0.442	88	75	61	-28	-34	1.31E-6	4.80E-6	> 1.00E-4
K-562	0.211	1.584	1.582	1.481	1.221	0.289	0.185	100	92	74	6	-13	2.22E-6	2.05E-5	> 1.00E-4
MOLT-4	0.683	2.487	2.504	2.445	1.413	0.539	0.567	101	98	40	-21	-17	6.82E-7	4.54E-6	> 1.00E-4
RPMI-8226	0.610	2.131	2.147	2.044	1.568	0.352	0.374	101	94	63	-42	-39	1.33E-6	3.96E-6	> 1.00E-4
SR	0.236	0.707	0.645	0.630	0.409	0.231	0.221	87	84	37	-2	-7	5.21E-7	8.72E-6	> 1.00E-4
Non-Small Cell Lung Cancer															
A549/ATCC	0.570	2.212	2.174	2.144	2.291	1.240	0.414	98	96	105	41	-27	7.18E-6	3.96E-5	> 1.00E-4
EKVX	0.660	2.099	2.011	1.836	1.656	0.913	0.703	94	82	69	18	3	2.36E-6	> 1.00E-4	> 1.00E-4
HOP-62	0.685	1.425	1.393	1.274	1.323	0.891	0.397	96	80	86	28	-42	4.17E-6	2.50E-5	> 1.00E-4
HOP-92	1.070	1.700	1.601	1.554	1.542	0.891	0.608	84	77	75	-17	-43	1.87E-6	6.57E-6	> 1.00E-4
NCI-H226	0.980	2.464	2.418	2.332	2.111	1.283	0.543	97	91	76	20	-45	2.95E-6	2.06E-5	> 1.00E-4
NCI-H23	0.590	1.794	1.714	1.665	1.496	0.884	0.487	93	89	75	24	-18	3.13E-6	3.82E-5	> 1.00E-4
NCI-H322M	0.868	1.921	1.859	1.832	1.748	1.086	0.947	94	92	84	21	8	3.42E-6	> 1.00E-4	> 1.00E-4
NCI-H460	0.268	2.685	2.718	2.475	2.073	0.305	0.115	101	91	75	2	-57	2.17E-6	1.06E-5	7.57E-5
NCI-H522	1.072	2.422	2.375	2.262	2.058	0.899	0.671	97	88	73	-16	-37	1.81E-6	6.59E-6	> 1.00E-4
Colon Cancer															
COLO 205	0.429	1.122	1.148	1.007	0.864	0.144	0.097	104	83	63	-66	-78	1.25E-6	3.06E-6	7.46E-6
HCC-2998	0.490	1.692	1.672	1.672	1.585	0.309	0.081	98	98	91	-37	-84	2.09E-6	5.14E-6	1.90E-5
HCT-116	0.226	1.957	1.902	1.633	1.645	0.113	0.038	97	81	82	-50	-83	1.75E-6	4.17E-6	9.96E-6
HCT-15	0.466	2.733	2.747	2.520	2.341	0.572	0.453	101	91	83	5	-3	2.63E-6	4.22E-5	> 1.00E-4
HT29	0.332	1.747	1.942	1.839	1.771	0.394	0.351	114	107	102	4	1	3.40E-6	> 1.00E-4	> 1.00E-4
KM12	0.427	2.174	2.201	2.016	1.970	0.653	0.295	102	91	88	13	-31	3.22E-6	1.97E-5	> 1.00E-4
SW-620	0.359	2.293	2.229	1.995	1.933	0.650	0.315	97	85	81	15	-12	2.97E-6	3.53E-5	> 1.00E-4
CNS Cancer															
SF-268	0.687	2.032	2.025	1.908	1.878	0.886	0.562	99	91	89	15	-18	3.33E-6	2.80E-5	> 1.00E-4
SF-295	0.827	2.650	2.611	2.467	2.428	1.683	0.821	98	90	88	47	.	8.41E-6	9.66E-5	> 1.00E-4
SF-539	1.072	2.757	2.655	2.604	2.409	1.257	0.684	94	91	79	11	-36	2.69E-6	1.71E-5	> 1.00E-4
SNB-19	0.424	1.474	1.438	1.404	1.330	0.723	0.453	97	93	86	28	3	4.24E-6	> 1.00E-4	> 1.00E-4
SNB-75	0.917	1.745	1.611	1.464	1.495	1.044	0.846	84	66	70	15	-8	2.31E-6	4.60E-5	> 1.00E-4
U251	0.476	1.883	1.876	1.878	1.782	0.678	0.259	99	100	93	14	-46	3.51E-6	1.73E-5	> 1.00E-4
Melanoma															
LOX IMVI	0.263	1.556	1.494	1.387	1.079	0.087	0.046	95	87	63	-67	-83	1.26E-6	3.06E-6	7.41E-6
MALME-3M	0.758	1.163	1.152	1.101	1.071	0.593	0.437	97	85	77	-22	-42	1.88E-6	6.03E-6	> 1.00E-4
M14	0.396	1.549	1.510	1.412	1.395	0.229	0.099	97	88	87	-42	-75	1.92E-6	4.70E-6	1.72E-5
MDA-MB-435	0.386	2.087	1.996	1.932	1.740	0.041	0.042	95	91	80	-89	-89	1.50E-6	2.96E-6	5.85E-6
SK-MEL-2	1.239	2.493	2.520	2.387	2.326	1.126	0.705	102	92	87	-9	-43	2.42E-6	8.03E-6	> 1.00E-4
SK-MEL-28	0.626	1.875	1.843	1.803	1.801	0.982	0.490	97	94	94	28	-22	4.70E-6	3.69E-5	> 1.00E-4
SK-MEL-5	0.615	2.983	2.933	2.859	2.512	0.949	0.035	98	95	80	14	-94	2.86E-6	1.35E-5	3.90E-5
UACC-257	1.416	2.586	2.507	2.404	2.503	2.073	1.246	93	84	93	56	-12	1.23E-5	6.66E-5	> 1.00E-4
UACC-62	0.893	2.838	2.723	2.558	2.345	0.975	0.480	94	86	75	4	-46	2.24E-6	1.21E-5	> 1.00E-4
Ovarian Cancer															
IGROV1	0.661	2.072	2.107	1.991	1.756	1.020	0.619	103	94	78	25	-6	3.38E-6	6.31E-5	> 1.00E-4
OVCAR-3	0.432	1.481	1.558	1.380	1.079	0.323	0.221	107	90	62	-25	-49	1.36E-6	5.11E-6	> 1.00E-4
OVCAR-4	0.767	1.624	1.587	1.401	1.190	0.835	0.680	96	74	49	8	-11	9.35E-7	2.56E-5	> 1.00E-4
OVCAR-5	0.665	1.872	1.836	1.784	1.826	0.907	0.475	97	93	96	20	-29	4.04E-6	2.58E-5	> 1.00E-4
OVCAR-8	0.682	2.479	2.422	2.365	2.254	1.336	0.721	97	94	87	36	2	5.41E-6	> 1.00E-4	> 1.00E-4
NCI/ADR-RES	0.579	2.027	1.976	1.931	1.838	1.174	0.568	97	93	87	41	-2	6.39E-6	8.99E-5	> 1.00E-4
SK-OV-3	0.895	1.897	1.904	1.756	1.799	1.440	0.793	101	86	90	54	-11	1.17E-5	6.71E-5	> 1.00E-4
Renal Cancer															
786-0	0.707	2.492	2.473	2.414	2.378	1.372	0.662	99	96	94	37	-6	5.94E-6	7.12E-5	> 1.00E-4
A498	1.494	2.251	2.055	1.964	1.942	1.368	0.992	74	62	59	-8	-34	1.37E-6	7.50E-6	> 1.00E-4
ACHN	0.430	1.874	1.921	1.771	1.719	0.690	0.463	103	93	89	18	2	3.56E-6	> 1.00E-4	> 1.00E-4
RXF 393	0.919	1.528	1.510	1.532	1.405	0.838	0.600	97	101	80	-9	-35	2.17E-6	7.94E-6	> 1.00E-4
SN12C	0.422	1.597	1.553	1.513	1.483	0.545	0.416	96	93	90	10	-1	3.20E-6	7.59E-5	> 1.00E-4
TK-10	0.882	1.906	2.006	2.101	2.163	1.418	0.887	110	119	125	52	.	1.11E-5	> 1.00E-4	> 1.00E-4
UO-31	0.771	1.790	1.639	1.596	1.408	1.052	0.715	85	81	62	28	-7	2.28E-6	6.18E-5	> 1.00E-4
Prostate Cancer															
PC-3	0.394	1.376	1.296	1.261	1.169	0.269	0.163	92	88	79	-32	-59	1.83E-6	5.17E-6	4.74E-5
DU-145	0.465	1.781	1.830	1.795	1.682	0.733	0.404	104	101	92	20	-13	3.88E-6	4.06E-5	> 1.00E-4
Breast Cancer															
MCF7	0.548	2.600	2.494	2.269	1.797	0.614	0.473	95	84	61	3	-14	1.54E-6	1.55E-5	> 1.00E-4
MDA-MB-231/ATCC	0.642	1.427	1.399	1.308	1.200	0.574	0.397	96	85	71	-11	-38	1.81E-6	7.40E-6	> 1.00E-4
HS 578T	1.159	2.155	2.105	1.959	2.064	1.672	1.374	95	80	91	52	22	1.13E-5	> 1.00E-4	> 1.00E-4
BT-549	1.264	2.058	2.064	1.914	1.843	1.036	0.809	101	82	73	-18	-36	1.79E-6	6.33E-6	> 1.00E-4
T-47D	0.865	1.530	1.481	1.343	1.109	0.899	0.744	93	72	37	5	-14	4.16E-7	1.84E-5	> 1.00E-4
MDA-MB-468	0.732	1.367	1.392	1.298	1.186	0.569	0.344	104	89	71	-22	-53	1.69E-6	5.78E-6	7.94E-5

National Cancer Institute Developmental Therapeutics Program Mean Graphs	NSC : D - 795311/1	Units :Molar	SSPL :0YVT	EXP. ID :1702NS56
	Report Date :March 20, 2017		Test Date :February 13, 2017	

Panel/Cell Line	Log_{10}GI50	Log_{10}TGI	Log_{10}LC50
**Leukemia**			
CCRF-CEM	-5.71	> -4.00	> -4.00
HL-60(TB)	-5.88	-5.32	> -4.00
K-562	-5.65	-4.69	> -4.00
MOLT-4	-6.17	-5.34	> -4.00
RPMI-8226	-5.88	-5.40	> -4.00
SR	-6.28	-5.06	> -4.00
**Non-Small Cell Lung Cancer**			
A549/ATCC	-5.14	-4.40	> -4.00
EKVX	-5.63	> -4.00	> -4.00
HOP-62	-5.38	-4.60	> -4.00
HOP-92	-5.73	-5.18	> -4.00
NCI-H226	-5.53	-4.69	> -4.00
NCI-H23	-5.50	-4.42	> -4.00
NCI-H322M	-5.47	> -4.00	> -4.00
NCI-H460	-5.66	-4.97	-4.12
NCI-H522	-5.74	-5.18	> -4.00
**Colon Cancer**			
COLO 205	-5.90	-5.51	-5.13
HCC-2998	-5.68	-5.29	-4.72
HCT-116	-5.76	-5.38	-5.00
HCT-15	-5.58	-4.37	> -4.00
HT29	-5.47	> -4.00	> -4.00
KM12	-5.49	-4.71	> -4.00
SW-620	-5.53	-4.45	> -4.00
**CNS Cancer**			
SF-268	-5.48	-4.55	> -4.00
SF-295	-5.08	-4.02	> -4.00
SF-539	-5.57	-4.77	> -4.00
SNB-19	-5.37	> -4.00	> -4.00
SNB-75	-5.64	-4.34	> -4.00
U251	-5.45	-4.76	> -4.00
**Melanoma**			
LOX IMVI	-5.90	-5.51	-5.13
MALME-3M	-5.73	-5.22	> -4.00
M14	-5.72	-5.33	-4.77
MDA-MB-435	-5.82	-5.53	-5.23
SK-MEL-2	-5.62	-5.10	> -4.00
SK-MEL-28	-5.33	-4.43	> -4.00
SK-MEL-5	-5.54	-4.87	-4.41
UACC-257	-4.91	-4.18	> -4.00
UACC-62	-5.65	-4.92	> -4.00
**Ovarian Cancer**			
IGROV1	-5.47	-4.20	> -4.00
OVCAR-3	-5.87	-5.29	> -4.00
OVCAR-4	-6.03	-4.59	> -4.00
OVCAR-5	-5.39	-4.59	> -4.00
OVCAR-8	-5.27	> -4.00	> -4.00
NCI/ADR-RES	-5.19	-4.05	> -4.00
SK-OV-3	-4.93	-4.17	> -4.00
**Renal Cancer**			
786-0	-5.23	-4.15	> -4.00
A498	-5.86	-5.12	> -4.00
ACHN	-5.45	> -4.00	> -4.00
RXF 393	-5.66	-5.10	> -4.00
SN12C	-5.50	-4.12	> -4.00
TK-10	-4.96	> -4.00	> -4.00
UO-31	-5.64	-4.21	> -4.00
**Prostate Cancer**			
PC-3	-5.74	-5.29	-4.32
DU-145	-5.41	-4.39	> -4.00
**Breast Cancer**			
MCF7	-5.81	-4.81	> -4.00
MDA-MB-231/ATCC	-5.74	-5.13	> -4.00
HS 578T	-4.95	> -4.00	> -4.00
BT-549	-5.75	-5.20	> -4.00
T-47D	-6.38	-4.74	> -4.00
MDA-MB-468	-5.77	-5.24	-4.10
_MID	-5.59	-4.69	-4.12
Delta	0.79	0.84	1.11
Range	1.47	1.53	1.23

GI50

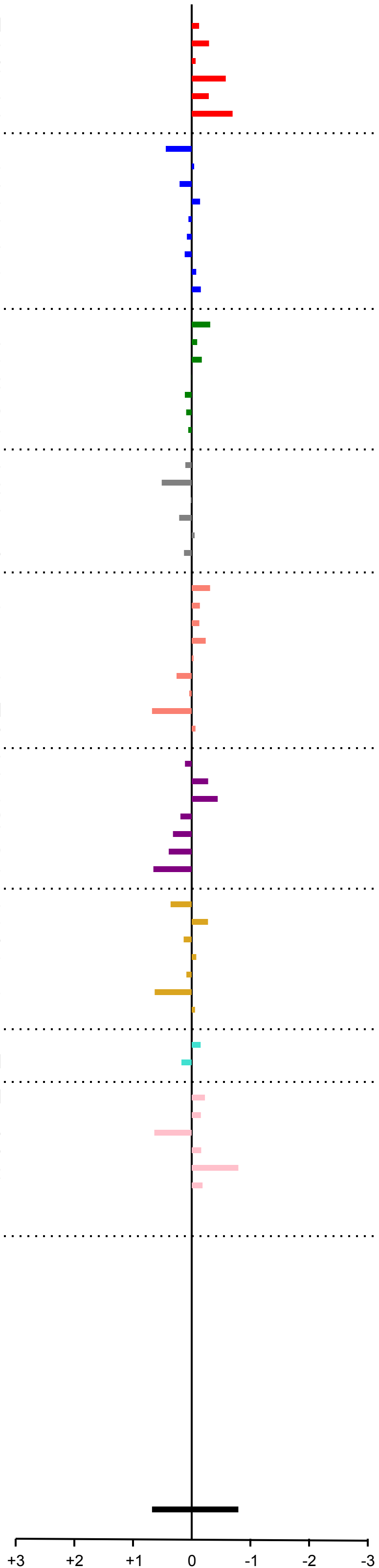

TGI

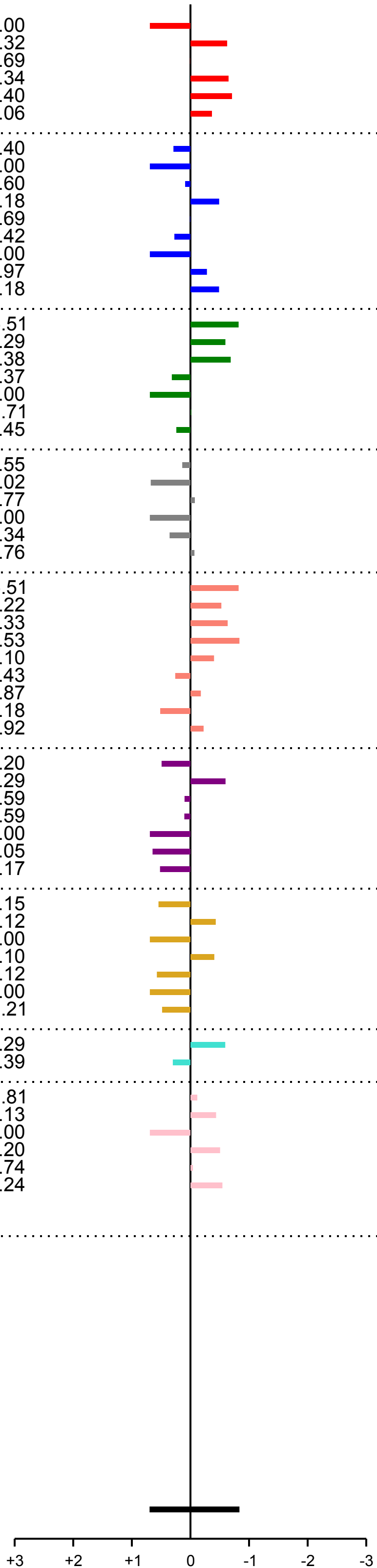

LC50

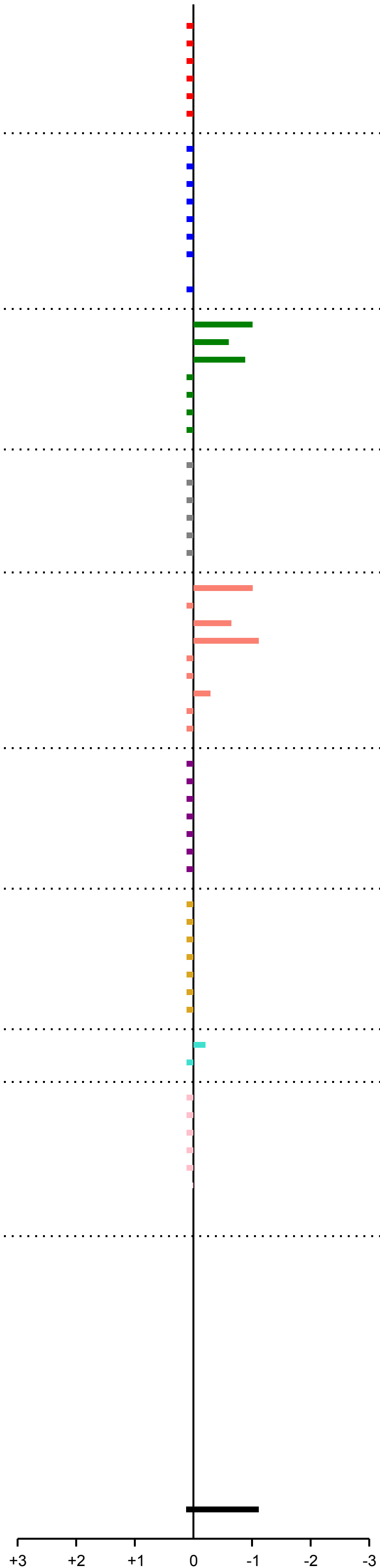

National Cancer Institute Developmental Therapeutics Program<br>Dose Response Curves	NSC : D - 795311/1	SSPL:0YVT	EXP. ID:1702NS56
	Report Date:March 20, 2017	Test Date:February 13, 2017	

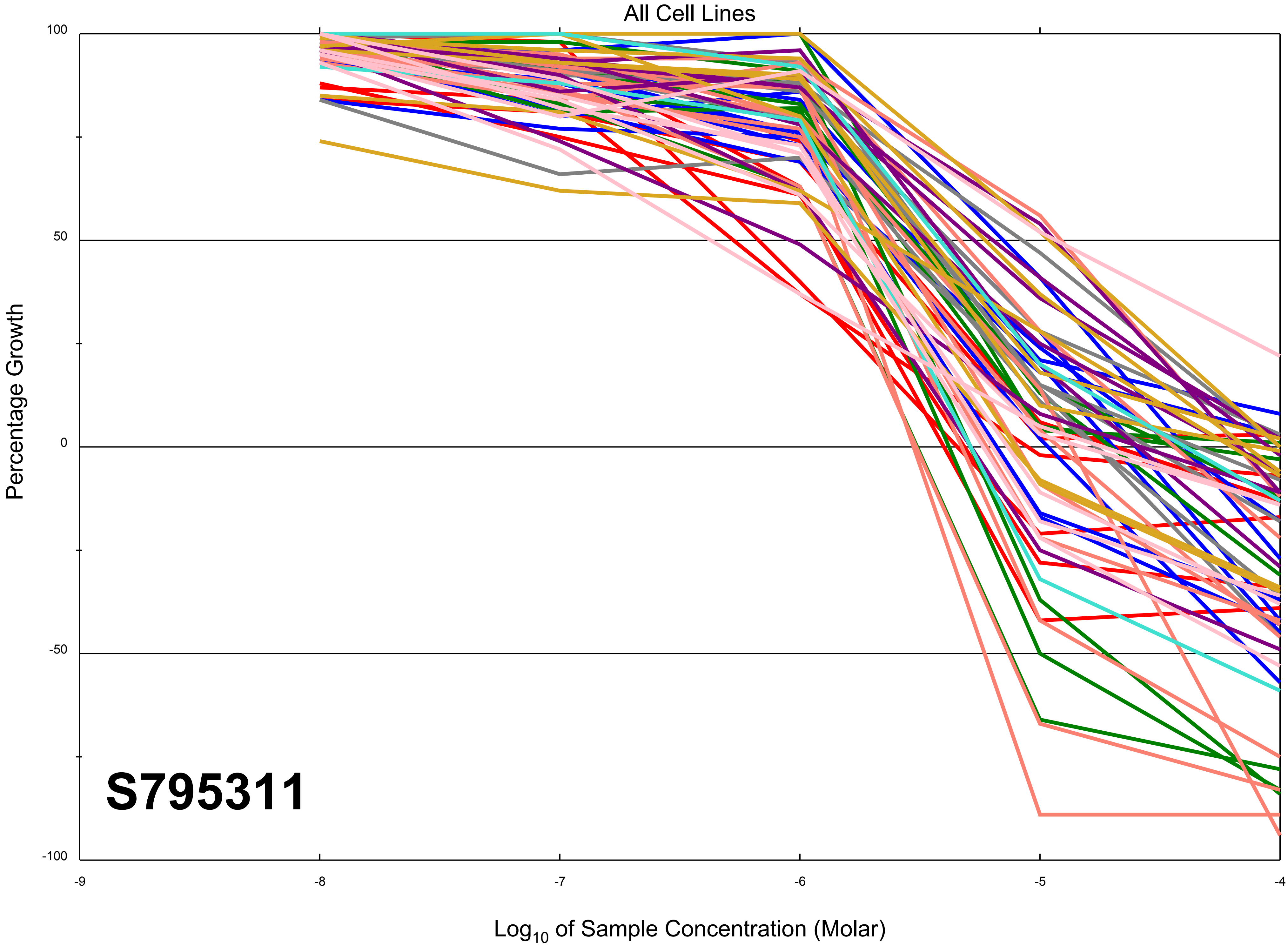

Biological Evaluations

In vitro anti-proliferative activity assay

The three examined cancer cell lines (non-small cell lung A-549, Breast MDA-MB-231 and colon HCT-116 cancer cells) were obtained from American Type Culture Collection (ATCC). The cells were maintained in Dulbecco's modified Eagle's medium (DMEM) supplemented with 10% heat inactivated fetal calf serum (GIBCO), penicillin (100 U/ml) and streptomycin (100 µg/ml) at 37 ^oC in humidified atmosphere containing 5% CO_2. Cells at a concentration of 0.50 x 10^6 were grown in a 25 cm^2 flask in 5 ml of culture medium.

The anti-proliferative activity of the tested compounds was measured *in vitro* using the Sulfo-Rhodamine-B stain (SRB) assay. Briefly, Cells were inoculated in 96-well microtiter plate ($5X10^4$ cells/ well) for 24 h before treatment with the tested compounds to allow attachment of cell to the wall of the plate. Tested compounds were dissolved in DMSO at 1 mg/ml immediately before use and diluted to the appropriate volume just before addition to the cell culture. Different concentrations of tested compounds, doxorubicin and sorafenib were added to the cells (three wells were prepared for each individual dose). Cells were incubated with the compounds for 48 h at 37°C and in atmosphere of 5% CO_2. After 48 h cells were fixed, washed, and stained for 30 min with 0.4% (w/v) SRB dissolved in 1% acetic acid. Unbound dye was removed by four washes with 1% acetic acid, and attached stain was recovered with Tris-EDTA buffer. Color intensity was measured in an ELISA reader. The relation between percent of surviving fraction and drug concentration is plotted to get the survival curve for each cell line. The concentration required for 50% inhibition of cell viability (IC_{50}) was calculated and the results are given in Table 1. The results were compared to the effect of the reference drug doxorubicin.

NCI-in vitro antitumor activity towards 59 cancer cell lines

The cytotoxicity assays were performed at National Cancer Institute (NCI), Bethesda, USA (against 59 cell lines). The human tumor cell lines of the cancer screening panel were grown in RPMI 1640 medium containing 5% fetal bovine serum and 2 mM L-glutamine. For a typical screening experiment, cells were inoculated into 96 well microtiter plates in 100 µ at plating densities ranging from 5000 to 40,000 cells/well depending on the doubling time of individual cell lines. After cell inoculation, the microtiter plates were incubated at 37 ^oC, 5% CO_2, 95% air and 100% relative humidity for 24 h prior to addition of experimental drugs. After 24 h, two

plates of each cell line were fixed *in situ* with trichloroacetic acid (TCA), to represent a measurement of the cell population for each cell line at the time of drug addition (T_z). Experimental drugs were solubilized in dimethyl sulfoxide at 400-fold the desired final maximum test concentration and stored frozen prior to use. At the time of drug addition, an aliquot of frozen concentrate was thawed and diluted to twice the desired final maximum test concentration with complete medium containing 50 µg/ml gentamicin. Additional four, 10-fold or ½ log serial dilutions were made to provide a total of five drug concentrations plus control. Aliquots of 100 µl of these different drug dilutions were added to the appropriate microtiter wells already containing 100 µl of medium, resulting in the required final drug concentrations. Triplicate wells were prepared for each individual dose. Following drug addition, the plates were incubated for an additional 48 h at 37 ^oC, 5% CO_2, 95% air, and 100% relative humidity. For adherent cells, the assay was terminated by the addition of cold TCA. Cells were fixed *in situ* by the gentle addition of 50 µl of cold 50% (w/v) TCA (final concentration, 10% TCA) and incubated for 60 min at 4 ^oC. The supernatant was discarded, and the plates were washed five times with tap water and air dried. Sulforhodamine B (SRB) solution (100 µl) at 0.4% (w/v) in 1% acetic acid was added to each well, and plates were incubated for 10 min at room temperature. After staining, unbound dye was removed by washing five times with 1% acetic acid and the plates were air dried. Bound stain was subsequently solubilized with 10 mM trizma base, and the absorbance was read on an automated plate reader at a wavelength of 515 nm. For suspension cells, the methodology was the same except that the assay was terminated by fixing settled cells at the bottom of the wells by gently adding 50 µl of 80% TCA (final concentration, 16% TCA). Using the seven absorbance measurements [time zero (*Tz*), control growth (*C*), and test growth in the presence of drug at the five concentration levels (T_i)], the percentage growth was calculated at each of the drug concentration levels. Percentage growth inhibition was calculated as:

$[(T_i - T_z) / (C - T_z)] \times 100$ for concentrations for which $T_i \geq T_z$

$[(T_i - T_z) / T_z] \times 100$ for concentrations for which $T_i < T_z$

Three dose response parameters were calculated for each experimental compound: Growth inhibition of 50% (GI_{50}) was calculated when $[(T_i - T_z) / (C - T_z)] \times 100 = 50$. The compound concentration resulting in total growth inhibition (TGI) was calculated when $T_i = T_z$. The LC_{50}

indicating a net loss of cells following treatment was calculated when $[(T_i - T_z) / T_z] \times 100 = -50$.

4.2.4. Cell Cycle Analysis

T-47D cells were treated with conjugate **8a** for 24 h (at its IC_{50} concentration), and then cells were washed twice with ice-cold phosphate buffered saline (PBS). Subsequently, the treated cells were collected by centrifugation, fixed in ice-cold 70% (*v*/*v*) ethanol, washed with PBS, re-suspended with 100 µg/mL RNase, stained with 40 µg/mL PI, and analyzed by flow cytometry using FACS Calibur (Becton Dickinson, BD, Franklin Lakes, NJ, USA). The cell cycle distributions were calculated using CellQuest software 5.1 (Becton Dickinson).

Annexin V-FITC Apoptosis Assay

Phosphatidylserine externalization was assayed using Annexin V-FITC/PI apoptosis detection kit (BD Biosciences, San Jose, CA, USA) according to the manufacturer's instructions. T-47D cells were cultured to a monolayer then treated with conjugate **8a** at its IC_{50} concentration. Cells were then harvested via trypsinization, and rinsed twice in PBS followed by binding buffer. Moreover, cells were re-suspended in 100 µL of binding buffer with the addition of 1 µL of FITC-Annexin V (Becton Dickinson BD Pharmingen™, Heidelberg, Germany) followed by an incubation period of 30 min at 4 °C. Cells were then rinsed in binding buffer and resuspended in 150 µL of binding buffer with the addition of 1 µL of DAPI (1 µg/µL in PBS) (Invitrogen, Life Technologies, Darmstadt, Germany). Cells were then analyzed using the flow cytometer BD FACS Canto II (BD Biosciences, San Jose, CA, USA) and the results were interpreted with FlowJo7.6.4 software (Tree Star, Ashland, OR, USA).

CDK2 inhibition assay

The kinase was assayed with histone H1 in the presence of 15 µM ATP, 0.05 µCi [γ-^{33}P]ATP and of the test compound in a final volume of 10 µL, all in a reaction buffer (60 mM HEPES-NaOH, pH 7.5, 3 mM $MgCl_2$, 3 mM $MnCl_2$, 3 µM Na-orthovanadate, 1.2 mM DTT, 2.5 µg / 50 µl $PEG_{20.000}$). The reactions were stopped by adding 5 µL of 3 % aq. H_3PO_4. Aliquots were spotted onto P-81 phosphocellulose (Whatman), washed 3× with 0.5 % aq. H_3PO_4 and finally air-dried. Kinase inhibition was quantified using a FLA-7000 digital image analyzer (Fujifilm).

5.1.2.1. 1-Ethyl-3-(hydrazonomethyl)-1H-indole (**5a**)

Yield 64 %, MP: 139-140 °C; ^{1}H NMR δ *ppm*: 1.32, 1.38 (t, 3H, *J* = 8 Hz, *N*-CH_2-C<u>H</u>$_3$), 4.12, 4.22 (t, 2H, *J* = 8 Hz, *N*-C$\underline{H_2}$-CH_3), 6.24 (brs, 2H, NH_2), 7.23-7.31 (m, 2H, H-5 and H-6 of indol), 7.54 (d, 1H, *J* = 8 Hz, H-7 of indol), 7.94 (s, 1H, H-2 of indol), 8.41 (d, 1H, *J* = 8 Hz, H-4 of indol), 8.91 (s, 1H, -C$\underline{H}$=N-); IR: 3378 (NH_2); Anal. calcd. For $C_{11}H_{13}N_3$: C, 70.56; H, 7.00; N, 22.44; Found C, 70.32; H, 6.97; N, 22.61.

5.1.2.2. 3-(Hydrazonomethyl)-1-isopropyl-1H-indole (**5c**)

Yield 57 %, MP: 186-188°C; ^{1}H NMR δ *ppm*: 1.51 (d, 6H, -CH(C$\underline{H_3}$)$_2$), 4.77-4.85 (m, 1H, *N*-CH), 7.06 (brs, 2H, NH_2), 7.20-7.30 (m, 2H, H-5 and H-6 of indol), 7.60 (d, 1H, *J* = 8 Hz, H-7 of indol), 8.07 (s, 1H, H-2 of indol), 8.36 (d, 1H, *J* = 8 Hz, H-4 of indol), 8.89 (s, 1H, -C$\underline{H}$=N-); ^{13}C NMR δ *ppm*: 22.82 (-CH($\underline{C}H_3$)$_2$), 47.55 (*N*-CH),, 110.95, 112.02, 121.30, 122.76, 123.07, 125.80, 131.21, 137.08, 155.13; IR: 3342 (NH_2); Anal. calcd. For $C_{12}H_{15}N_3$: C, 71.61; H, 7.51; N, 20.88; Found C, 71.87; H, 7.50; N, 20.79.

5.1.2.3. 1-Allyl-3-(hydrazonomethyl)-1H-indole (**5d**)

Yield 73 %, MP: 177-180°C; ^{1}H NMR δ *ppm*: 4.90 (d, 2H, *J*= 8 Hz, *N*-CH_2), 5.10-5.11 (m, 1H, oliefinic H), 5.20-5.24 (m, 1H, oliefinic H), 6.01-6.11 (m, 1H, oliefinic H), 7.06 (brs, 2H, NH_2), 7.20-7.30 (m, 2H, Ar-H), 7.52-7.59 (m, 1H, Ar-H), 7.92-7.96 (m, 1H, Ar-H), 8.37-8.40 (m, 1H, Ar-H), 8.89-8.91 (m, 1H, Ar-H); ^{13}C NMR δ *ppm*: 47.90, 48.86 (N-CH2), 110.57, 110.93, 111.14, 112.03, 112.05, 117.81, 120.21, 121.23, 121.38, 122.82, 123.15, 123.25, 125.75, 125.81, 134.29, 134.64, 134.87, 134.95, 137.55, 137.59, 155.05, 155.20; IR: 3362 (NH_2); HRMS (ESI) for $C_{12}H_{14}N_3$, calcd 200.11822, found 200.11849 $[M+H]^+$; Anal. calcd. For $C_{12}H_{13}N_3$: C, 72.33; H, 6.58; N, 21.09; Found C, 72.20; H, 6.55; N, 21.15.

5.1.3.1. 5-Chloro-3-(((1-ethyl-1H-indol-3-yl)methylene)hydrazono)indolin-2-one (**7**)

Yield 78 %, MP: 202-203 °C; ^{1}H NMR δ *ppm*: 1.44 (t, 3H, *J* = 8 Hz, -CH_2-C$\underline{H_3}$), 4.33 (q, 2H, *J* = 8 Hz, CH_2), 6.93-6.96 (m, 1H, Ar-H), 7.34-7.39 (m, 1H, Ar-H), 7.45-7.55 (m, 2H, Ar-H), 7.71 (d, 1H, *J* = 8 Hz, Ar-H), 8.30-8.35 (m, 2H, Ar-H), 8.50 (s, 1H, Ar-H), 9.00 (s, 1H, Ar-H), 10.93 (s, 1H, NH); ^{13}C NMR δ *ppm*: 15.54 (CH_3), 41.77 (CH_2), 111.88, 111.91, 112.52, 113.09,

117.58, 118.73, 121.88, 122.66, 124.19, 125.44, 126.07, 126.54, 127.57, 128.44, 132.42, 134.41, 137.82, 139.63, 143.26, 144.73, 146.28, 148.80, 163.37 (C=O); IR: 3401 (NH), 1698 (C=O); Anal. calcd. For $C_{19}H_{15}ClN_4O$: C, 65.05; H, 4.31; N, 15.97; Found C, 65.18; H, 4.30; N, 16.05.

5.1.3.2. 5-Methyl-3-(((1-propyl-1H-indol-3-yl)methylene)hydrazono)indolin-2-one (**8a**)

Yield 74 %, MP: 195-197 °C; ^1H NMR δ *ppm*: 0.87 (t, 3H, *J* = 8 Hz, -$CH_2C\underline{H_3}$), 1.79-189 (m, 2H, -$C\underline{H_2}CH_3$), 2.20 (s, 3H, CH_3 of isatin), 4.22 (t, 2H, *J* = 8 Hz, *N*-CH_2), 6.80 (m, 1H, Ar-H), 7.19 (t, 1H, *J* = 8 Hz, Ar-H), 7.33 (d, 2H, *J* = 8 Hz, Ar-H), 7.67 (d, 1H, *J* = 8 Hz, Ar-H), 8.26 (s, 1H, Ar-H), 8.34 (s, 1H, Ar-H), 8.40 (d, 1H, *J* = 8 Hz, Ar-H), 8.95 (s, 1H, Ar-H), 10.66 (s, 1H, NH); IR: 3375 (NH), 1701 (C=O); Anal. calcd. For $C_{21}H_{20}N_4O$: C, 73.23; H, 5.85; N, 16.27; Found C, 72.97; H, 5.88; N, 16.32.

5.1.3.3. 3-(((1-propyl-1H-indol-3-yl)methylene)hydrazono)-5-(trifluoromethoxy)indolin-2-one (**8b**)

Yield 77 %, MP: 189-190 °C; ^1H NMR δ *ppm*: 0.89 (t, 3H, *J* = 8 Hz, -$CH_2C\underline{H_3}$), 1.83-192 (m, 2H, -$C\underline{H_2}CH_3$), 4.28 (t, 2H, *J* = 8 Hz, *N*-CH_2), 7.01-7.05 (m, 1H, Ar-H), 7.25 (t, 1H, *J* = 8 Hz, Ar-H), 7.38 (t, 1H, *J* = 8 Hz, Ar-H), 7.48 (t, 1H, *J* = 8 Hz, Ar-H), 7.72 (d, 1H, *J* = 8 Hz, Ar-H), 8.27 (d, 1H, *J* = 8 Hz, Ar-H), 8.34 (s, 1H, Ar-H), 8.44-4.46 (m, 1H, Ar-H), 9.03 (s, 1H, Ar-H), 10.96 (s, 1H, NH); ^{13}C NMR δ *ppm*: 11.51 (-CH_3), 23.23 (CH_3-$\underline{C}H_2$), 48.37 (*N*-CH_2), 111.78, 114.15, 116.06, 119.48, 120.68, 122.40, 123.91, 125.35, 126.26, 127.78, 128.72, 131.61, 133.46, 133.98, 136.11, 140.60, 143.58, 163.98 (C=O); IR: 3391 (NH), 1699 (C=O); HRMS (ESI) for $C_{21}H_{18}F_3N_4O_2$, calcd 415.13764, found 415.13783 $[M+H]^+$; Anal. calcd. For $C_{21}H_{17}F_3N_4O_2$: C, 60.87; H, 4.14; N, 13.52; Found C, 60.69; H, 4.11; N, 13.50.

5.1.3.4. 5-Nitro-3-(((1-propyl-1H-indol-3-yl)methylene)hydrazono)indolin-2-one (**8c**)

Yield 83 %, MP: 247-250°C; ^1H NMR δ *ppm*: 0.86-.92 (m, 3H, -$CH_2C\underline{H_3}$), 1.78-191 (m, 2H, -$C\underline{H_2}CH_3$), 4.18,426 (t, 2H, *J* = 8 Hz, *N*-CH_2), 7.07 (d, 0.5H, *J* = 8 Hz, Ar-H), 7.19-7.29 (m, 1.5H, Ar-H), 7.39 (d, 1H, *J* = 8 Hz, Ar-H), 7.56, 7.70 (d, 1H, *J* = 8 Hz, Ar-H), 7.94 (s, 1H, Ar-H), 8.29-8.47 (m, 2H, Ar-H), 8.87 (s, 1H, Ar-H), 9.06, 9.36 (s, 1H, Ar-H), 11.45, 11.65 (s, 1H, NH); IR: 3396 (NH), 1710 (C=O); Anal. calcd. For $C_{20}H_{17}N_5O_3$: C, 63.99; H, 4.56; N, 18.66; Found C, 64.21; H, 4.54; N, 18.60.

5.1.3.5. 3-(((1-Isopropyl-1H-indol-3-yl)methylene)hydrazono)indolin-2-one (**9**)

Yield 78 %, MP: 260-261°C; ^{1}H NMR δ *ppm*: 1.53 (brs, 6H, -CH(C<u>H</u>$_3$)$_2$), 4.85 (brs, 1H, *N*-CH), 6.87-6.97 (m, 2H, Ar-H), 7.07-7.23 (m, 2H, Ar-H), 7.30-7.46 (m, 2H, Ar-H), 7.70-7.95 (m, 1H, Ar-H), 7.36-7.46 (m, 1H, Ar-H), 8.74-8.94 (m, 1H, Ar-H), 9.55 (s, 1H, Ar-H), 10.55 (s, 1H, NH); IR: 3391 (NH), 1700 (C=O); Anal. calcd. For $C_{20}H_{18}N_4O$: C, 72.71; H, 5.49; N, 16.96; Found C, 72.60; H, 5.48; N, 17.02.

5.1.3.6. 3-(((1-Allyl-1H-indol-3-yl)methylene)hydrazono)indolin-2-one (**10a**)

Yield 80 %, MP: 220-222 °C; ^{1}H NMR δ *ppm*: 4.97 (d, 2H, *J* = 8 Hz, *N*-CH$_2$), 5.15-5.19 (m, 1H, Ar-H), 5.24-5.27 (m, 1H, Ar-H), 6.04-6.13 (m, 1H, Ar-H), 6.77 (d, 0.5H, *J* = 8 Hz, Ar-H), 6.92 (d, 1H, *J* = 8 Hz, Ar-H), 7.07 (t, 1H, *J* = 8 Hz, Ar-H), 7.36-7.38 (m, 2H, Ar-H), 7.62-7.64 (m, 1H, Ar-H), 7.68 (d, 0.5H, *J* = 8 Hz, Ar-H), 8.26 (s, 1H, Ar-H), 8.31-8.35 (m, 1H, Ar-H), 8.37 (d, 1H, *J* = 8 Hz, Ar-H), 8.96 (s, 1H, Ar-H), 10.78 (s, 1H, NH); IR: 3410 (NH), 1701 (C=O); Anal. calcd. For $C_{20}H_{16}N_4O$: C, 73.15; H, 4.91; N, 17.06; Found C, 73.27; H, 4.95; N, 16.95.

5.1.3.7. 3-(((1-Allyl-1H-indol-3-yl)methylene)hydrazono)-5-fluoroindolin-2-one (**10b**)

Yield 77 %, MP: 235-237 °C; ^{1}H NMR δ *ppm*: 4.99-5.03 (m, 2H, *N*-CH$_2$), 5.15-5.27 (m, 2H, Ar-H), 6.05-6.12 (m, 1H, Ar-H), 6.91-6.94 (m, 1H, Ar-H), 7.31-7.36 (m, 3H, Ar-H), 8.15-8.19 (m, 1H, Ar-H), 8.28-8.34 (m, 2H, Ar-H), 8.98-9.03 (m, 2H, Ar-H), 10.91 (s, 1H, NH); ^{13}C NMR δ *ppm*: 47.79 (CH$_2$), 112.58, 114.55, 115.61, 115.87, 116.79, 117.71, 118.39, 121.33, 121.56, 122.76, 124.16, 125.52, 127.86, 130.11, 133.85, 142.32, 146.46, 159.21, 164.01 (C=O); IR: 3394 (NH), 1697 (C=O); HRMS (ESI) for $C_{20}H_{16}FN_4O$, calcd 347.13027, found 347.13019 $[M+H]^+$; Anal. calcd. For $C_{20}H_{15}FN_4O$: C, 69.35; H, 4.37, N, 16.18; Found C, 69.51; H, 4.39, N, 16.25.

5.1.3.8. 3-(((1-Allyl-1H-indol-3-yl)methylene)hydrazono)-7-fluoroindolin-2-one (**10c**)

Yield 82 %, MP: 208-210 °C; ^{1}H NMR δ *ppm*: 4.98 (d, 2H, *J* = 8 Hz, *N*-CH$_2$), 5.15-5.21(m, 1H, Ar-H), 5.24-5.28 (m, 1H, Ar-H), 6.04-6.14 (m, 1H, Ar-H), 7.03-7.09 (m, 2H, Ar-H), 7.37-7.40 (m, 5H, Ar-H), 8.30 (s, 1H, Ar-H), 8.99 (s, 1H, Ar-H), 11.55 (s, 1H, NH); ^{13}C NMR δ *ppm*: 49.52 (CH$_2$), 112.04, 118.39, 118.79, 121.68, 121.86, 121.95, 122.90, 123.85, 123.91, 124.08, 124.90, 130.38, 132.63, 132.77, 133.84, 137.99, 141.29, 144.75, 163.58 (C=O); IR: 3380 (NH),

1702 (C=O); Anal. calcd. For $C_{20}H_{15}FN_4O$: C, 69.35; H, 4.37, N, 16.18; Found C, 69.21; H, 4.32, N, 16.15.

5.1.3.9. 3-(((1-Allyl-1H-indol-3-yl)methylene)hydrazono)-5-chloroindolin-2-one (**10d**)

Yield 75 %, MP: 267-269 °C; ^{1}H NMR δ *ppm*: 4.99 (d, 2H, *J* = 8 Hz, *N*-CH_2), 5.16-5.22 (m, 1H, Ar-H), 5.25-5.28 (m, 1H, Ar-H), 6.05-6.14 (m, 1H, Ar-H), 6.94 (d, 2H, *J* = 8 Hz, Ar-H), 7.33-7.39 (m, 1H, Ar-H), 7.45-7.55 (m, 2H, Ar-H), 7.66-7.69 (m, 1H, Ar-H), 8.30(s, 1H, Ar-H), 8.50-8.53 (m, 1H, Ar-H), 9.02 (s, 1H, Ar-H), 10.91 (s, 1H, NH); ^{13}C NMR δ *ppm*: 49.26 (CH_2), 112.11, 112.53, 113.12, 117.59, 118.45, 121.86, 122.70, 124.24, 126.10, 127.62, 128.44, 131.35, 132.50, 133.80, 137.73, 140.02, 142.13, 143.35, 163.17 (C=O); IR: 3401 (NH), 1699 (C=O); HRMS (ESI) for $C_{20}H_{16}ClN_4O$, calcd 363.10072, found 363.10054 $[M+H]^+$; Anal. calcd. For $C_{20}H_{15}ClN_4O$: C, 66.21; H, 4.17, N, 15.44; Found C, 66.34; H, 4.13, N, 15.39.

5.1.3.10. 3-(((1-Allyl-1H-indol-3-yl)methylene)hydrazono)-5-(trifluoromethoxy)indolin-2-one (**10e**)

Yield 80 %, MP: 228-229 °C; ^{1}H NMR δ *ppm*: 4.98 (d, 2H, *J* = 8 Hz, *N*-CH_2), 5.17-5.22 (m, 1H, Ar-H), 5.25-5.28 (m, 1H, Ar-H), 6.04-6.14 (m, 1H, Ar-H), 7.0-7.03 (m, 2H, Ar-H), 7.25-7.29 (m, 1H, Ar-H), 7.37-7.7.44 (m, 2H, Ar-H), 7.47-7.53 (m, 1H, Ar-H), 7.65-7.67 (m, 1H, Ar-H), 8.31(s, 1H, Ar-H), 9.05 (s, 1H, Ar-H), 10.98 (s, 1H, NH); ^{13}C NMR (DMSO-d_6) δ *ppm*: 49.27 (CH_2), 112.10, 112.72, 116.96, 118.02, 118.47, 120.73, 121.89, 122.47, 124.30, 126.29, 127.95, 133.76, 138.12, 140.38, 143.27, 143.64, 145.07, 146.94, 148.95, 163.91 (C=O); IR: 3398 (NH), 1711 (C=O); HRMS (ESI) for $C_{21}H_{16}F_3N_4O_2$, calcd 413.12199, found 413.12260 $[M+H]^+$; Anal. calcd. For $C_{21}H_{15}F_3N_4O_2$: C, 61.17; H, 3.67; N, 13.59; Found C, 61.01; H, 3.63; N, 13.55.
